# Conserved mRNA-granule component Scd6 targets Dhh1 to repress translation initiation and activates Dcp2-mediated mRNA decay *in vivo*

**DOI:** 10.1371/journal.pgen.1007806

**Published:** 2018-12-07

**Authors:** Quira Zeidan, Feng He, Fan Zhang, Hongen Zhang, Allan Jacobson, Alan G. Hinnebusch

**Affiliations:** 1 Eunice Kennedy Shriver National Institute of Child Health and Development, National Institutes of Health, Bethesda, MD, United States of America; 2 Department of Microbiology and Physiological Systems, University of Massachusetts Medical School, Worcester, MA, United States of America; Case Western Reserve University, UNITED STATES

## Abstract

Scd6 protein family members are evolutionarily conserved components of translationally silent mRNA granules. Yeast Scd6 interacts with Dcp2 and Dhh1, respectively a subunit and a regulator of the mRNA decapping enzyme, and also associates with translation initiation factor eIF4G to inhibit translation in cell extracts. However, the role of Scd6 in mRNA turnover and translational repression in vivo is unclear. We demonstrate that tethering Scd6 to a *GFP* reporter mRNA reduces mRNA abundance via Dcp2 and suppresses reporter mRNA translation via Dhh1. Thus, in a *dcp2Δ* mutant, tethered Scd6 reduces *GFP* protein expression with little effect on mRNA abundance, whereas tethered Scd6 has no impact on *GFP* protein or mRNA expression in a *dcp2Δ dhh1Δ* double mutant. The conserved LSm domain of Scd6 is required for translational repression and mRNA turnover by tethered Scd6. Both functions are enhanced in a *ccr4Δ* mutant, suggesting that the deadenylase function of Ccr4-Not complex interferes with a more efficient repression pathway enlisted by Scd6. Ribosome profiling and RNA-Seq analysis of *scd6Δ* and *dhh1Δ* mutants suggests that Scd6 cooperates with Dhh1 in translational repression and turnover of particular native mRNAs, with both processes dependent on Dcp2. Our results suggest that Scd6 can (i) recruit Dhh1 to confer translational repression and (ii) activate mRNA decapping by Dcp2 with attendant degradation of specific mRNAs in vivo, in a manner dependent on the Scd6 LSm domain and modulated by Ccr4.

## Author summary

Previous work showed that Scd6 homologs in Drosophila, Xenopus, and humans are associated with translationally repressed mRNAs in RNA granules, and that they interact with other mRNA silencing factors, including homologs of RNA helicase Dhh1. However, there is little evidence that such Scd6 homologs are critical for translational repression or degradation of specific mRNAs in vivo. Yeast Scd6 interacts with the mRNA decapping enzyme and was shown to inhibit translation in cell extracts through binding to cap-binding translation initiation factor eIF4G. However, it was unknown whether Scd6 represses translation or accelerates degradation of any specific mRNAs in vivo, or whether Scd6 requires the decapping enzyme or its activators for such events. Here we show that tethering Scd6 stimulates the degradation or translational repression of reporter mRNAs in yeast, collaborating with Dhh1 for translational repression and the decapping enzyme Dcp2/Dcp1 for mRNA turnover. Using ribosome profiling we further identify groups of native mRNAs that appear to be targeted for degradation or translational repression by Scd6 and Dhh1 and find unexpectedly that Dcp2 is necessary for translational repression as well as enhanced degradation of most such mRNAs. Thus, Scd6 partners with Dhh1 and Dcp2 to mediate translational repression and degradation of specific mRNAs in vivo.

## Introduction

After being transcribed and processed in the nucleus, and exported to the cytoplasm, mRNAs can either engage with the translational machinery for protein synthesis, undergo storage in a translationally silent state, or be targeted for degradation. Cellular mRNAs can alternate between these processes, and translation, storage, and decay influence each other in multiple ways to regulate gene expression [1]. In general, mRNAs selected for translation are thought to establish interactions of their 5’-cap and 3’-poly(A) tail appendages with proteins from the translational machinery. mRNAs are activated for translation by binding to the mRNA cap of eukaryotic initiation factor eIF4F (comprised of cap-binding protein eIF4E, scaffolding protein eIF4G, and helicase eIF4A) and association of poly(A)-binding protein (PABP) with the poly(A) tail; and interactions between eIF4G and PABP can form a “closed-loop” mRNP competent for initiation. Further interactions between eIF4G and other translation initiation factors associated with the 43S pre-initiation complex (PIC) pre-assembled on the small (40S) ribosomal subunit serve to recruit mRNA and form a 48S PIC competent for subsequent mRNA scanning and start codon selection (reviewed in [2]).

The mRNA decay machinery can compete with the translation initiation machinery for access to cap and poly(A) tail of the transcript [1]. Degradation of mRNA is generally initiated by removal of the poly(A) tail through sequential deadenylation reactions by the Pan2/Pan3 and Ccr4-Not complexes, followed by loss of associated PABP [3]. Turnover can proceed in the 3’ to 5’ direction via the cytoplasmic exosome, or in the 5’ to 3’ direction via removal of the cap by the Dcp2/Dcp1 enzyme complex and exonucleolytic digestion by Xrn1 [4]. Decapping is a highly regulated step that irreversibly commits mRNAs for complete digestion [5]. One regulatory mechanism is thought to include formation of complexes comprised of Dcp1/2, Xrn1, and distinct sets of Dcp2 interactors that modulate both mRNA substrate recruitment or catalysis by Dcp2 [4, 6]. These decapping activators include DEAD-box helicase Dhh1, Pat1, Edc3, the Lsm1-7 complex, and Scd6 [4]. Decapping activators can function by distinct mechanisms: (i) Scd6, Dhh1, and Pat1 can inhibit translation initiation by blocking formation of a 48S PIC in vitro, which in turn favors mRNA decapping [7]; (ii) Pat1 and Edc3 can directly bind Dcp2 and stimulate its catalytic activity in vitro and in vivo [7–9]; and (iii) Dhh1 can detect reductions in ribosome transit at non-optimal codons and further inhibit translation elongation, eliciting acceleration of transcript decapping in vivo [6, 10].

Based on their ability to inhibit translation initiation in vitro, it has been proposed that the decapping activators Dhh1 and Scd6 can direct their target mRNAs from a translationally active state to an mRNP state competent for mRNA storage or decapping [11]. Scd6 belongs to a highly conserved protein family with orthologs in humans (hRAP55/Lsm14), *Xenopus laevis* (xRAP55), *Drosophila melanogaster* (Tral), *Caenorhabditis elegans* (CAR-1), *Trypanosoma brucei* (TbSCD6), and fission yeast (Sum2), among others [12]. All family members contain a conserved N-terminal LSm (like-Sm) domain, followed by central DFDF-FFD-TGF boxes, and variable numbers of C-terminal RGG/RGX motifs [12, 13]. In metazoans and *Plasmodium*, Scd6 associates with Dhh1 homologs, other proteins, and mRNAs to form stable mRNPs that contain translationally silent transcripts subject to developmental regulation [12]. For example, in *Drosophila*, Scd6 and Dhh1 homologs, Tral and Me31B, belong to a repression complex containing CUP, an inhibitor of eIF4E-eIF4G association, shown to control the translation of certain mRNAs with key functions in embryogenesis [14, 15]. This complex might function more broadly to repress translation of many mRNAs during early embryogenesis, possibly by coating the mRNA [16, 17]. An Scd6 homolog in *Xenopus*, xRAPB, associates with the Dhh1 ortholog Xp54 in translationally inactive maternal transcripts in stored mRNPs; although xRAPB appears to oppose rather than promote Xp54 function in repressing translation in oocytes [18]. A longer *Xenopus* variant, xRAP55 represses translation in vitro, and decreases reporter protein levels when tethered to a reporter mRNA in cell extracts, dependent on its N-terminal LSm domain [19]; however, its role in oocytes is unclear [18].

In yeast and humans, Scd6 is required for the formation and accumulation of mRNA-containing cytoplasmic aggregates called Processing (P) bodies, and it also localizes to mRNA-containing stress granules under a variety of adverse conditions [19–22]. In addition to its ability to bind the decapping enzyme subunit Dcp2, yeast Scd6 can also interact with the other decapping activators Pat1 and Edc3 [7, 23–29], as well as with various members of the Lsm complex and Dhh1 [23, 24, 29]. In vitro, yeast Scd6 represses translation initiation by directly interacting with the C-terminal region of eIF4G (in the context of eIF4F) via the Scd6 C-terminal RGG domain, preventing recruitment of the 43S PIC to activated mRNA and formation of the 48S PIC [7, 22]. The RGG domain is also required for overexpressed Scd6 to inhibit cell growth and produce stress granules [22]. Yeast Scd6 can also interact with other translation components, including proteins of the small and large ribosomal subunits, PABP, eIF4B, eIF5, and eEF1A [23, 25, 29]. Like yeast Scd6, the Arabidopsis homolog, Dcp5, was shown to repress translation in vitro [30]. The homolog in *Trypanosoma brucei* is present in cytoplasmic granules and appears to be a general repressor of translation, even though it does not exhibit an association with the Dhh1 homolog [13, 16, 17] that is otherwise conserved in yeast, worms, flies, and vertebrates [31].

Considerable evidence points to an important role for yeast Scd6 in cytoplasmic post-transcriptional control. It interacts with the decapping enzyme and its regulators, can inhibit 48S PIC assembly in vitro, and overexpression of its gene induces stress granule formation and inhibits cell growth in a manner requiring its C-terminal eIF4G-interaction domain. Nevertheless, it has not been demonstrated that Scd6 mediates the degradation or translational repression of any specific mRNA in yeast cells. In this study, we show that tethering Scd6 to two different reporter mRNAs in vivo evokes Dhh1-dependent repression of translation initiation, which is accompanied by reporter-specific Dcp2-mediated mRNA turnover. Both Scd6 functions are dependent on its LSm domain, and are enhanced by depletion of the Ccr4 deadenylase of the Ccr4-Not complex in vivo. By ribosome profiling we further provide evidence that Scd6 cooperates with Dhh1 in repressing the mRNA abundance and translation of particular native mRNAs in nutrient-replete medium, and present evidence that both functions require Dcp2. Thus, Scd6 appears to be an important component of the gene expression control network in yeast cells, acting at the levels of both translation initiation and mRNA turnover.

## Results

### Tethered Scd6 represses expression of a *GFP* reporter mRNA

**Fig 1:**
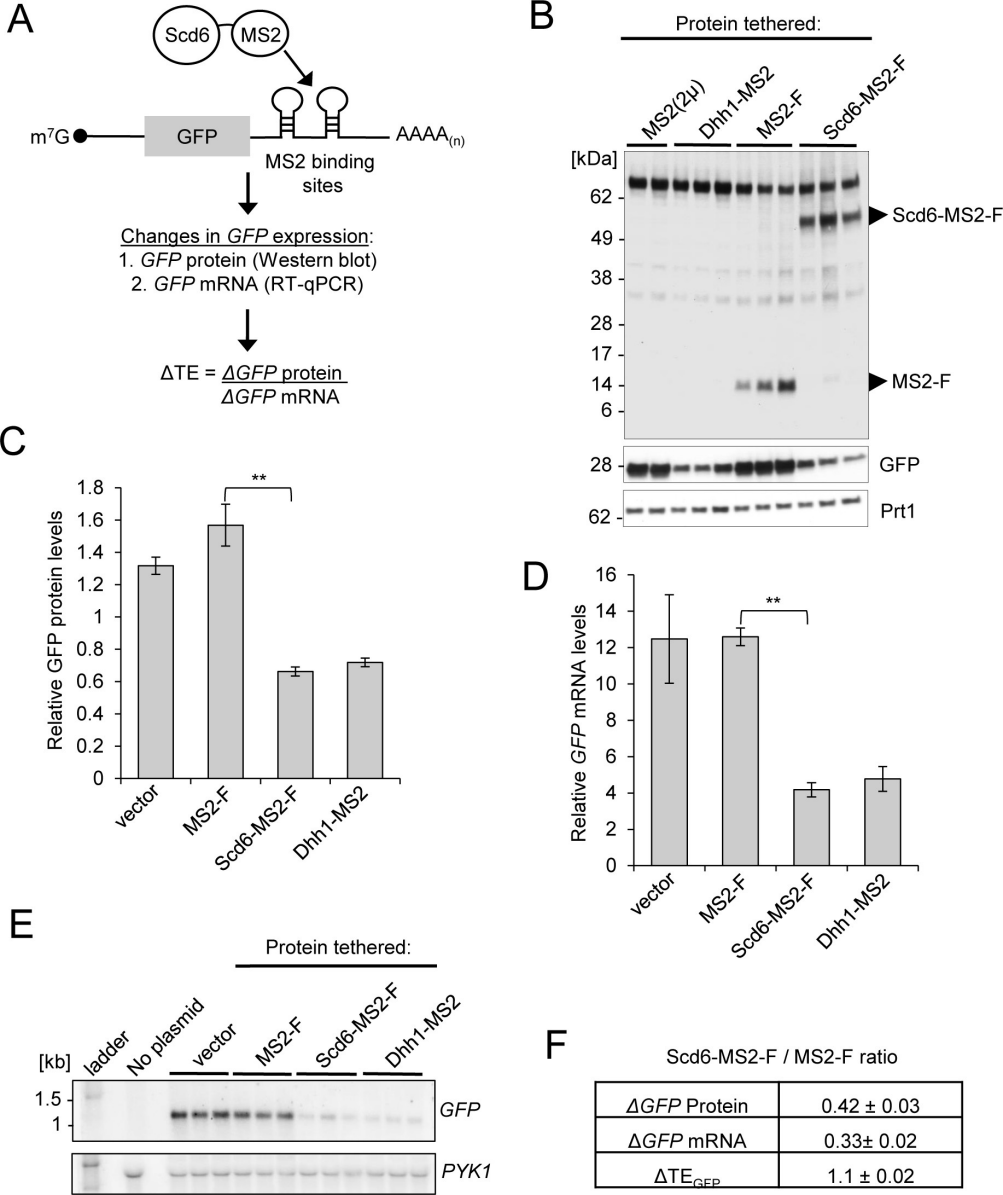
Tethered Scd6 destabilizes *GFP* mRNA *in vivo*. **(A)** Schema of the MS2 CP tethering assay with Scd6-MS2-F fusion protein targeted to stem-loops in the 3’ UTR of the *GFP* reporter; with summary of assays used to measure *GFP* protein or mRNA abundance and translation efficiency (TE) of the reporter. **(B)** WT cells (BY4741) were co-transformed with an expression plasmid for MS2(2μ) (pJC398), Dhh1-MS2 (pJC236), Scd6-MS2-F (pQZ127) or MS2-F (pQZ130), along with *GFP* reporter plasmid (pJC429), cultured in synthetic complete medium without leucine or uracil (SC-L-U) with 2% galactose/2% raffinose replacing dextrose at 30°C for at least two cell divisions, and harvested at A_600_ ~0.6–0.7. Total protein was extracted under denaturing conditions and subjected to Western blot analysis with antibodies against FLAG, GFP or Prt1 (as loading control). Typical results from three biological replicates are shown. **(C)** Relative *GFP* protein signal normalized to Prt1 protein signal by densitometry analysis of samples in (B). Average results (± S.E.M.s) from at least three biological replicates are shown. **(D)** Transformants from (A), and WT strain BY4741 harboring empty vector YCpLac111 and *GFP* reporter pJC429, were cultured as in (B). Total RNA was extracted from whole cell extracts (WCEs) and reporter mRNA abundance was quantified by RT-qPCR, relative to that of *ACT1* mRNA. Average results (± S.E.M.s) from at least three biological replicates are shown. **(E)** Total RNA samples from (D) were subjected to Northern blot analysis and probed for *GFP* mRNA; blots were stripped and re-probed for *PYK1* mRNA as a loading control. **(F)** Changes (Δ) in expression of *GFP* reporter protein or mRNA, or reporter TE, on tethering Scd6-MS2-F, as ratios of values in cells harboring Scd6-MS2-F versus MS2-F. Mean values (± S.E.M.s) were determined from at least three biological replicates. Calculations of S.E.M.s for mean ratios of *GFP* protein and mRNA expression shown in (F), and determination of P-values from significance testing of differences in mean values in (C-D) using an unpaired Student’s t-test, were conducted as described in the supporting file S1 Text “Analysis and Explanation of Supporting Data Files”. P-values are summarized as: **, P <0.01; *, P <0.05. P-values for these and all other statistical tests performed in this study can be found in the supplementary data files provided for the respective figures.

Previously, it was shown that Scd6 interacts with mRNA decapping factors and can inhibit translation initiation in vitro [7, 22]; however, it was unknown whether Scd6 can reduce the abundance and repress the translation of specific mRNAs in vivo. Because native mRNA substrates of Scd6 were unknown, we employed a tethered-function assay to examine the consequences of Scd6 binding to reporter mRNA—an approach used successfully to demonstrate regulation of mRNA translation and turnover by the decapping activator/translational repressor Dhh1 [6]. A fusion of Scd6 to bacteriophage MS2 coat protein (CP), or MS2 CP alone, each tagged with three FLAG epitopes (henceforth, Scd6-MS2-F or MS2-F), was expressed in wild-type (WT) cells from mRNA containing the 5’ and 3’ untranslated regions (UTRs) of *SCD6* driven by the native *SCD6* promoter from a low copy plasmid. A *GFP* reporter mRNA, harboring the 5’UTR and 3’UTR of *PGK1* mRNA with tandem MS2 RNA recognition elements inserted in the 3’ UTR, and driven by a galactose-inducible *GAL1 UAS* and *PGK1* hybrid promoter [6], was expressed from a low copy plasmid in the same strains by culturing cells with galactose as carbon source. As a positive control, we expressed the Dhh1-MS2 fusion shown previously to evoke translational repression of the same *GFP* reporter [6]. Western blotting with anti-GFP antibodies and either Northern blotting or qRT-PCR analyses were conducted to measure steady-state expression of *GFP* protein and *GFP* reporter mRNA, respectively, and the ratio of these measurements yielded the translational efficiency (TE) of the reporter mRNA (Fig 1A).

Tethering Scd6-MS2-F reduced expression of *GFP* protein and reporter mRNA by comparable amounts, ~2.5- to 3-fold, compared to tethering MS2-F alone (Fig 1B–1D, Scd6-MS2-F vs. MS2-F; P <0.0001 in C & D). These results are similar to those observed on tethering Dhh1-MS2 versus MS2 alone expressed from a high-copy plasmid (Fig 1B–1D, Dhh1-MS2 vs. MS2(2μM)), in agreement with previous findings [6]. Expression of *GFP* protein and mRNA in cells expressing MS2-F was essentially indistinguishable from that measured in transformants of the same strain harboring empty vector (S1A Fig, Fig 1C and 1D). Northern analysis confirmed the repression of *GFP* mRNA by both Scd6-MS2-F and Dhh1-MS2 in comparison to empty vector and MS2-alone controls (Fig 1E). These results are consistent with the possibility that tethering Scd6 increases the rate of *GFP* mRNA turnover to lower its steady-state abundance, with attendant reduction in *GFP* protein expression. Owing to comparable repression of reporter protein and mRNA, tethered Scd6-MS2-F evokes little change in the TE of the *GFP* reporter (Fig 1F).

To determine whether tethering Scd6-MS2 to *GFP* mRNA accelerates its degradation, we measured the half-life of *GFP* mRNA following a shift from galactose to glucose medium that should repress the *GAL* promoter and halt new synthesis of reporter mRNA. On tethering Scd6-MS2-F, the half-life of *GFP* mRNA was significantly reduced from 2.8 ± 0.06 min (tethering MS2-F alone) to 1.9 ± 0.15 min (P = 0.03; S1B Fig). A similar fold-reduction in half-life was observed on tethering Dhh1-MS2 vs MS2 alone (2.3 ± 0.09 min vs. 3.20 ± 0.02 min, respectively; P = 0.01; S1C Fig). We conclude that tethered Scd6-MS2-F reduces *GFP* mRNA abundance by accelerating its degradation.

Because it was shown previously that Npl3 and Sbp1 can also repress translation initiation in vitro dependent on interaction of their RGG domains with eIF4G in a manner similar to Scd6 [22], we examined MS2 fusions of these proteins, expressed from plasmid constructs containing the native promoters and 5’- and 3’-UTRs of *NPL3* or *SBP1*, respectively, along with the corresponding MS2 control proteins. In contrast to results obtained for Scd6-MS2-F, neither Npl3-MS2-F nor Sbp1-MS2-F had any significant effect on *GFP* expression from the same reporter analyzed above (S2A, S2B, S2C and S2D Fig) despite being expressed at levels exceeding that of Scd6-MS2–F (S2E Fig). These results underscored the specificity of the effects of Scd6-MS2-F and led us to probe its mechanism of repression.

### Tethered Scd6 evokes Dcp2-dependent reduction of reporter mRNA abundance and Dhh1-dependent translational repression

**Fig 2:**
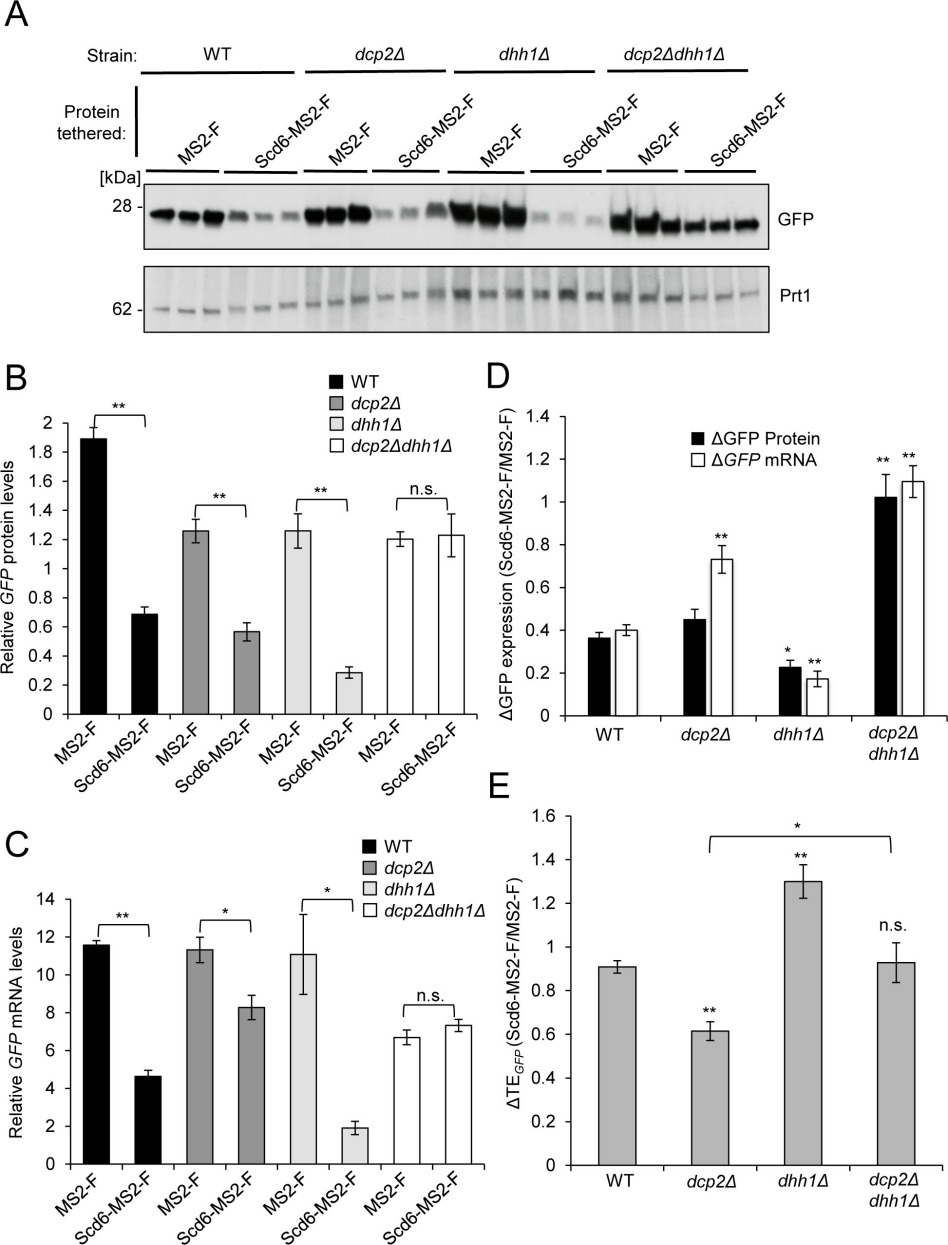
Evidence that translational repression of the *GFP* reporter by tethered Scd6-MS2-F is coupled to mRNA turnover via Dcp2 and requires Dhh1 *in vivo*. **(A-C)** Transformants of WT (HFY114), *dcp2Δ* (CFY1016), *dhh1Δ* (YQZ127), or *dcp2Δdhh1Δ* (QZY128) strains (isogenic in the W303 background) harboring expression plasmids for MS2-F (pQZ130] or Scd6-MS2-F (pQZ127) and *GFP* reporter pJC429, were analyzed for reporter protein (A-B) and mRNA (C) expression as in Fig 1B–1D. Mean values (± S.E.M.s) from three different biological replicates per group are shown. **(D-E)** Changes in expression of *GFP* reporter protein or mRNA (D), or in TE (E), on tethering Scd6-MS2-F versus MS2-F for the strains analyzed in panels A-C, reported as in Fig 1F. Mean values (± S.E.M.s) were determined from at least three biological replicates. Calculations of S.E.M.s for changes in mean ratios of *GFP* protein and mRNA expression shown in (D), and determination of P-values from significance testing of differences in mean values in (B-E) using an unpaired Student’s t-test, were conducted as described in the supporting file S1 Text “Analysis and Explanation of Supporting Data Files”. P-values are summarized as: **, P <0.01; *, P <0.05; n.s., not significant. In (E), the entries directly above the bars in cols. 2–3 refer to differences in mean values between the indicated mutants vs. WT (col. 1).

As Scd6 can bind to Dcp2, the catalytic component of the decapping enzyme [7], we asked whether the repression of reporter mRNA abundance evoked by tethered Scd6-MS2-F is attenuated in a *dcp2Δ* strain. The isogenic WT *DCP2* strain employed for this experiment exhibited reductions in *GFP* mRNA and *GFP* protein expression conferred by Scd6-MS2-F versus MS-F alone (Fig 2A–2D, WT data) equal to, or greater than those observed in the different WT strain employed above (Fig 1B–1D). Tethering Scd6-MS2-F to the *GFP* reporter in *dcp2Δ* cells reduced the abundance of *GFP* mRNA (Fig 2C, *dcp2Δ*, Scd6-MS2-F vs. MS2-F; P = 0.03). Importantly, however, the reduction in reporter mRNA abundance conferred by Scd6-MS2-F was diminished in *dcp2Δ* versus WT cells, with the ratio of *GFP* mRNA in cells expressing Scd6-MS2-F versus MS2-F increasing from ~0.4 in WT to ~0.7 in *dcp2Δ* cells (Fig 2D, white bars, WT vs. *dcp2Δ*; P = 0.009). Expression of *GFP* protein in cells containing MS2-F alone was reduced somewhat in the *dcp2Δ* vs. WT strain; however, this reduction occurred independently of tethering as it was also observed in the corresponding strains containing empty vector (S3A and S3B Fig). Tethering Scd6-MS2-F in *dcp2Δ* cells conferred a strong reduction in *GFP* protein expression compared to MS2-F alone (Fig 2B, *dcp2Δ*, Scd6-MS2-F vs. MS2-F; P = 0.002), that was only slightly smaller in magnitude compared to that seen in WT cells (Fig 2D, black bars, WT vs. *dcp2Δ*). As a consequence of relatively greater repression of *GFP* protein versus *GFP* mRNA on tethering Scd6-MS2-F versus MS2-F alone in the *dcp2Δ* mutant (Fig 2D, WT vs. *dcp2Δ*, black vs. white bars), the TE of *GFP* reporter mRNA is diminished in *dcp2Δ* versus WT cells by ~33% (Fig 2E, WT vs. *dcp2Δ*; P = 0.005). These findings suggest that: (i) Dcp2 is required for strong repression of reporter mRNA abundance, and (ii) translational repression of the *GFP* mRNA is unveiled in cells lacking Dcp2, where mRNA turnover by tethered Scd6-MS2 is diminished. Similar conclusions were reached previously for tethered Dhh1-MS2 [6].

The concordance between effects on *GFP* reporter mRNA and protein expression conferred by tethered Scd6-SM-2 (Fig 2A–2E) and Dhh1-MS2 (Sweet et al., 2012) in WT and *dcp2Δ* cells raised the possibility that Dhh1 is required for translational repression conferred by tethered Scd6-MS2-F. To address this possibility, we examined the regulation of *GFP* reporter expression in *dhh1Δ* cells. In contrast to our findings for isogenic *dcp2Δ* cells, we observed greater repression of reporter mRNA abundance by tethered Scd6-MS2-F in *dhh1Δ* versus WT cells (Fig 2C, Scd6-MS2-F vs. MS2-F, WT vs. *dhh1Δ*), with the Scd6-MS2-F/MS2-F repression ratio for *GFP* mRNA declining ~2.4-fold from ~0.4 in WT to ~0.17 in *dhh1Δ* cells (Fig 2D, white bars, WT vs. *dhh1Δ*; P = 0.007). Thus, unlike Dcp2, Dhh1 is dispensable for repression of reporter mRNA abundance by tethered Scd6-MS2-F. One possible explanation for the enhanced repression of *GFP* mRNA in *dhh1Δ* cells (vs. WT) might be that Dhh1 impedes Dcp1/Dcp2-mediated decapping of the reporter in the presence of tethered Scd6-MS2-F. Repression of *GFP* protein by Scd6-MS2-F was also intact in the *dhh1Δ* strain (Fig 2B, Scd6-MS2-F vs. MS2-F; P = 0.001), with an Scd6-MS2-F/MS2-F repression ratio for *GFP* protein ~1.6-fold lower than that seen in WT cells (Fig 2D, black bars, WT vs. *dhh1Δ*; P = 0.03). However, owing to even greater Scd6-MS2-F-mediated repression of *GFP* mRNA compared to *GFP* protein, the TE of the *GFP* reporter was increased by ~1.4-fold in *dhh1Δ* vs. WT cells (Fig 2E; P = 0.009). This observation is consistent with the possibility that Dhh1 contributes to translational repression by tethered Scd6-MS2-F.

We reasoned that if Dcp2 and Dhh1 are respectively required for repression of mRNA abundance and translation by tethered Scd6-MS2-F, then eliminating both proteins in a *dcp2Δ dhh1Δ* double mutant should abrogate repression of both *GFP* protein and *GFP* mRNA by Scd6-MS2-F. Indeed, nearly identical expression levels of *GFP* protein and mRNA were observed in the *dcp2Δ dhh1Δ* mutant whether expressing Scd6-MS2-F or MS2–F (Fig 2B and 2C, *dcp2Δ dhh1Δ*, Scd6-MS2-F vs. MS2-F), yielding near-unity Scd6-MS2-F/MS2-F repression ratios in this strain for both reporter mRNA and protein expression (Fig 2D, *dcp2Δ dhh1Δ*, black and white bars), which are markedly increased from the corresponding repression ratios of 0.36 and 0.40 in WT cells (Fig 2D, *dcp2Δ dhh1Δ* vs. WT, white and black bars; P = 0.004 (Protein), P = 0.0009 (mRNA)). Importantly, comparing the reporter TEs in the double mutant to the *dcp2Δ* single mutant reveals a loss of translational repression in *dcp2Δ dhh1Δ* cells (Fig 2E, *dcp2Δ dhh1Δ* vs. *dcp2Δ*; P = 0.04), supporting a requirement for Dhh1 in translational repression of *GFP* mRNA abundance by tethered Scd6-MS2-F. That the residual repression of mRNA abundance seen in the *dcp2Δ* single mutant is eliminated by *dhh1Δ* in the double mutant (Fig 2D, white bars, *dcp2Δ dhh1Δ* vs. *dcp2Δ*; P = 0.02) might indicate that Dhh1 mediates a Dcp2-independent mechanism of mRNA degradation evoked by tethered Scd6-MS2-F in *dcp2Δ* cells, such as involvement of the exosome or a ribosome-associated endonuclease. In summary, analyses of *dcp2Δ* and *dhh1Δ* mutants indicate that efficient repression of reporter mRNA abundance is dependent on Dcp2, whereas Dhh1 participates in translational repression, by tethered Scd6-MS2-F.

### Tethered Scd6-MS2-F alters the polysomal distribution of *GFP* mRNA

**Fig 3:**
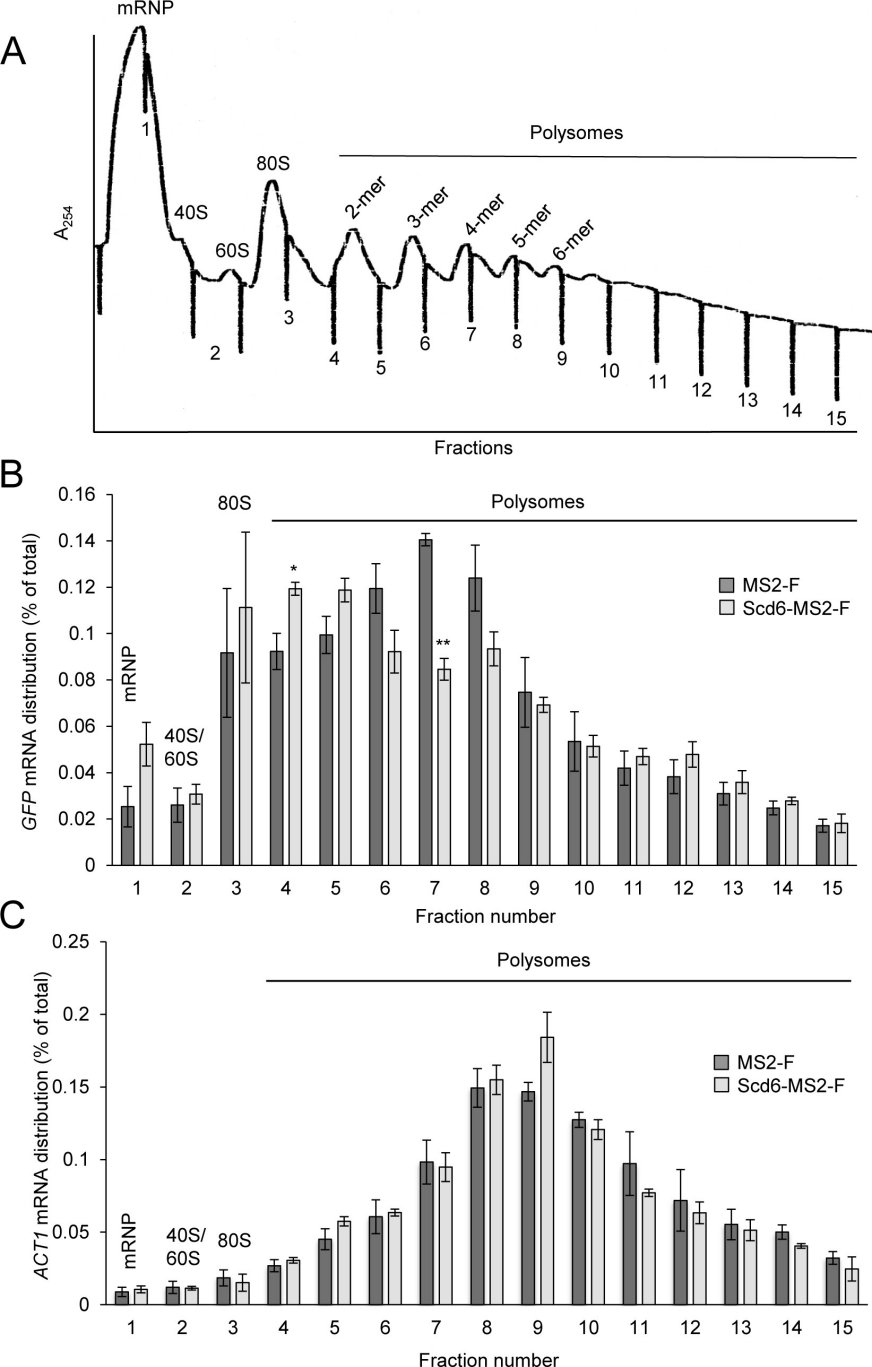
Polysome size distribution of *GFP* reporter mRNA is altered on tethering Scd6-MS2-F. **(A-C)** *dcp2Δ* transformants from Fig 2A harboring the *GFP* reporter and expressing Scd6-MS2-F or MS2-F were cultured as in Fig 1B and WCEs were separated by velocity sedimentation on sucrose density gradients and fractionated with continuous monitoring at A_254_. A representative profile is shown in (A). The abundance of *GFP* mRNA (B) or *ACT1* mRNA (C) was quantitated by RT-qPCR in total RNA extracted from the gradient fractions and plotted as the percentage of total RNA signal in the gradient summed across all fractions. Average results (and ±S.E.M.s) from three biological replicates are shown, which are presented in S4A and S4B Fig. An unpaired Student’s t-test showed that the proportions of mRNA in fractions 7 and 4, representing 4-mers and 1- to 2-mer polysomes, respectively, differed significantly between the Scd6-MS2-F vs. MS2-F transformants, with P-values summarized as: **, P <0.01; *, P <0.05.

Having observed that the occurrence of translational repression of *GFP* mRNA is unmasked in *dcp2Δ* cells, owing to partial stabilization of reporter mRNA, we examined the effect of tethered Scd6-MS2-F on the size distribution of *GFP* mRNA in polysomes, 80S monosomes, ribosomal subunits, and free mRNPs. Using sedimentation through sucrose density gradients and qRT-PCR analysis of *GFP* and actin (*ACT1*) mRNA in the gradient fractions, we reproducibly observed a shift in the fractions with peak abundance of *GFP* mRNA from polysomes containing 3 to 6 ribosomes (fractions 6–8, 3- to 6-mers) in cells expressing MS2-F alone to those containing 80S monosomes and 2- to 3-mers (fractions 3–5) in cells expressing Scd6-MS2–F (Fig 3A and 3B (P-values for fractions 7 and 4 of 0.031 and 0.0005); S4A and S4B Fig). By contrast, the fractions containing the peak abundance of actin mRNA (fractions 8–10, 5- to 8-mers) did not differ reproducibly between cells expressing Scd6-MS2-F versus MS2–F (Fig 3A & 3C). Despite the shift in peak *GFP* mRNA abundance from 3- to 5-mers to 2-mers and monosomes, there was no reduction in the proportion of *GFP* mRNA found in the largest polysomes near the bottom of the gradient on tethering Scd6-MS2-F versus MS–F (Fig 3A and 3B, fractions 10–15). One way to explain these findings is to propose that tethering Scd6-MS2-F inhibits translation to a greater extent at the initiation versus elongation stage of protein synthesis for the fraction of *GFP* mRNA that shifts towards monosomes and free mRNP, while inhibiting elongation more than initiation on a minority fraction that is retained in heavier polysomes.

### Elimination of Ccr4 enhances repression of both *GFP* mRNA abundance and translational efficiency by tethered Scd6-MS2-F

**Fig 4:**
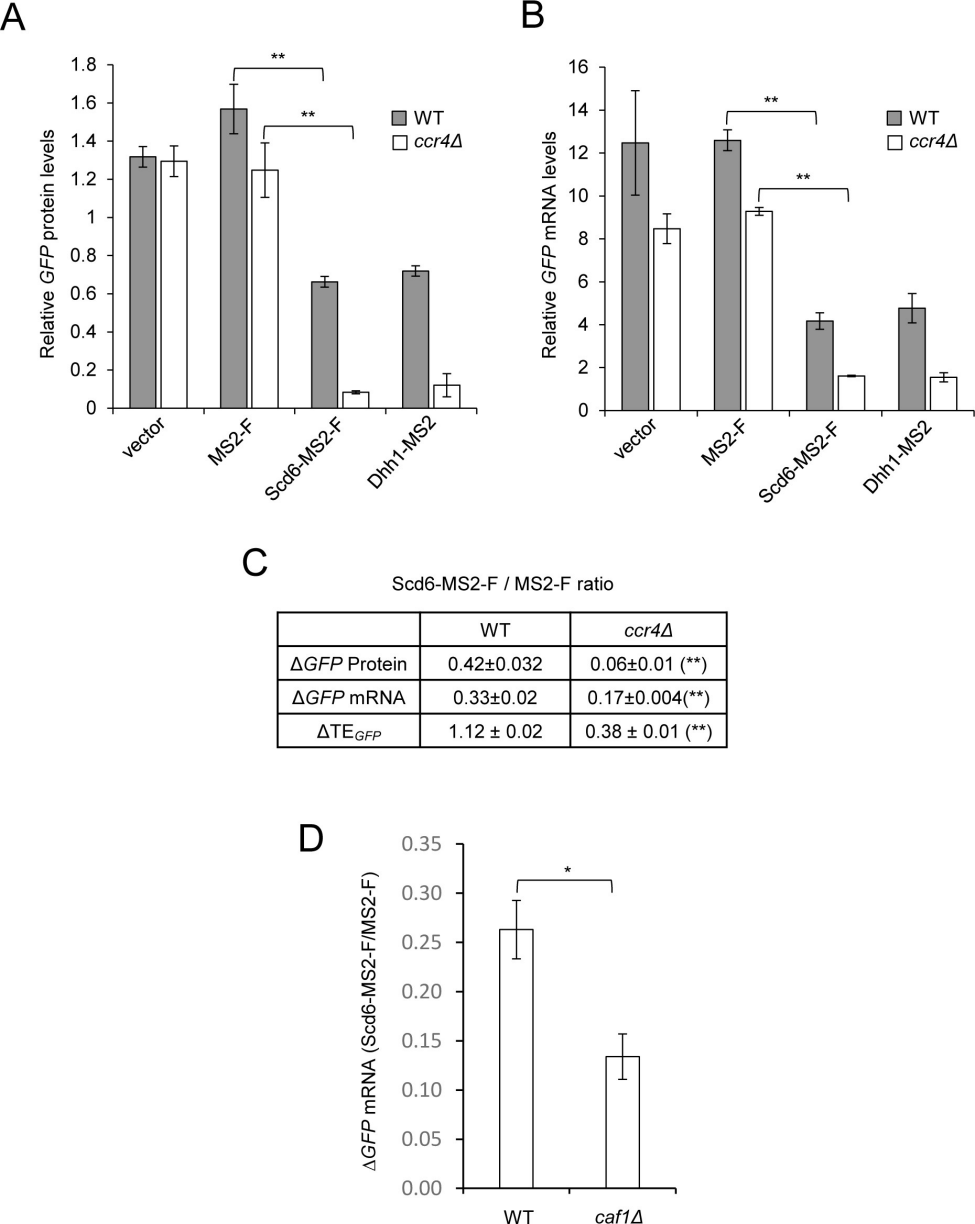
Evidence that both translational repression and mRNA turnover evoked by tethered Scd6-MS2-F are intensified in *ccr4Δ* cells. **(A-D)** Transformants of isogenic WT (BY4741) and *ccr4Δ* (387) strains (A-C), or WT (BY4741) and *caf1Δ* (7123) strains (D), harboring the *GFP* reporter and MS2 fusion expression plasmids described in Fig 1 were analyzed for reporter protein (A) and mRNA (B,D) expression as in Fig 1B–1D. Mean values (± S.E.M.s) were determined from at least three biological replicates. Calculations of S.E.M.s for changes in mean ratios of *GFP* protein and mRNA expression shown in (C & D), and determination of P-values from significance testing of differences in mean values in (A-D) using an unpaired Student’s t-test, were conducted as described in the supporting file S1 Text. P-values are summarized as: **, P <0.01; *, P <0.05. P-values for panel A are <0.0001 (WT) and 0.0012 (*ccr4Δ*); P-values for panel B are <0.0001 for both WT and *ccr4Δ* cells.

Having implicated Dcp2 in the reduction of reporter mRNA abundance by tethered Scd6-MS2-F, and noting that mRNA degradation in yeast frequently proceeds via removal of the poly(A) tail followed by decapping [4], we asked next whether repression of *GFP* mRNA expression by Scd6-MS2-F requires Ccr4, the major cytoplasmic deadenylase in yeast [32]. Unexpectedly, the reduction in reporter mRNA abundance by Scd6-MS2-F was enhanced rather than diminished in cells lacking Ccr4. We observed a modest (~30%) reduction in *GFP* mRNA levels in the *ccr4Δ* mutant containing empty vector or MS2–F (Fig 4B, vector & MS2-F, white vs. grey bars), but tethering Scd6-MS2-F conferred ~3-fold lower *GFP* mRNA abundance in *ccr4Δ* vs. WT cells (Fig 4B, Scd6-MS2-F, white vs. grey bars). The resulting >5-fold reduction in mRNA expression by Scd6-MS2-F versus MS2-F seen in the *ccr4Δ* mutant (Fig 4B, Scd6-MS2-F vs. MS2-F, white bars) is >2-fold larger than that observed in WT cells (Fig 4B, Scd6-MS2-F vs. MS2-F, grey bars; Fig 4C, Δ*GFP* mRNA, *ccr4Δ* vs. WT; P = 0.004). These results suggest that Ccr4 is not only dispensable for the reduced abundance of *GFP* mRNA conferred by tethered Scd6-MS2-F, but actually appears to impede a more efficient repression pathway that can operate in its absence.

Interestingly, the absence of Ccr4 also dramatically increased the repression of *GFP* protein expression by Scd6-MS2–F (Fig 4A, Scd6-MS2-F vs. MS2-F, white vs. grey bars), reducing the Scd6-MS2-F:MS2-F repression ratio for *GFP* protein from 0.42 to ~0.07 (Fig 4C, Δ*GFP* protein, *ccr4Δ* vs. WT; P = 0.0001). Owing to greater repression of *GFP* protein versus *GFP* mRNA in *ccr4Δ* cells, tethering Scd6-MS2-F decreased the TE of *GFP* mRNA to ~40% of the corresponding TE of ~1.1 observed in WT cells (Fig 4C, ΔTE_*GFP*_, *ccr4Δ* vs. WT; P<0.0001). These findings imply that the presence of Ccr4 also interferes with a more efficient mechanism for translational repression by tethered Scd6-MS2-F that can proceed in *ccr4Δ* cells. Similar findings were observed on tethering Dhh1-MS2 versus MS2-F to *GFP* mRNA, as repression of protein expression was greatly enhanced in *ccr4Δ* versus WT cells (Fig 4A and 4B, Dhh1-MS2-F vs. Scd6-MS2-F, grey vs. white bars), as previously observed with this same tethering system [6].

Recent findings suggest that the Caf1 subunit of the Ccr4-Not complex cooperates with Ccr4 in the deadenylation and degradation of a subset of yeast mRNAs with low codon optimality, functioning upstream of Dhh1-mediated decapping of such mRNAs [33]. Accordingly, we asked whether eliminating Caf1 from cells would diminish the repression of *GFP* mRNA abundance conferred by tethered Scd6-MS-F. At odds with this possibility, we observed a greater reduction in *GFP* mRNA abundance on tethering Scd6-MS2-F in *caf1Δ* compared to WT cells (Fig 4D; P = 0.01), similar to our findings with the *ccr4Δ* mutant (Fig 4C). Thus, the Ccr4-Not complex is dispensable for, and even seems to impede, the degradation of mRNAs promoted by tethered Scd6-MS2-F, as observed previously for tethered Dhh1 [6, 34].

### Dhh1-dependent translational repression of *lacZ* reporter mRNA by tethered Scd6-MS2-F is enhanced in the absence of Ccr4

**Fig 5:**
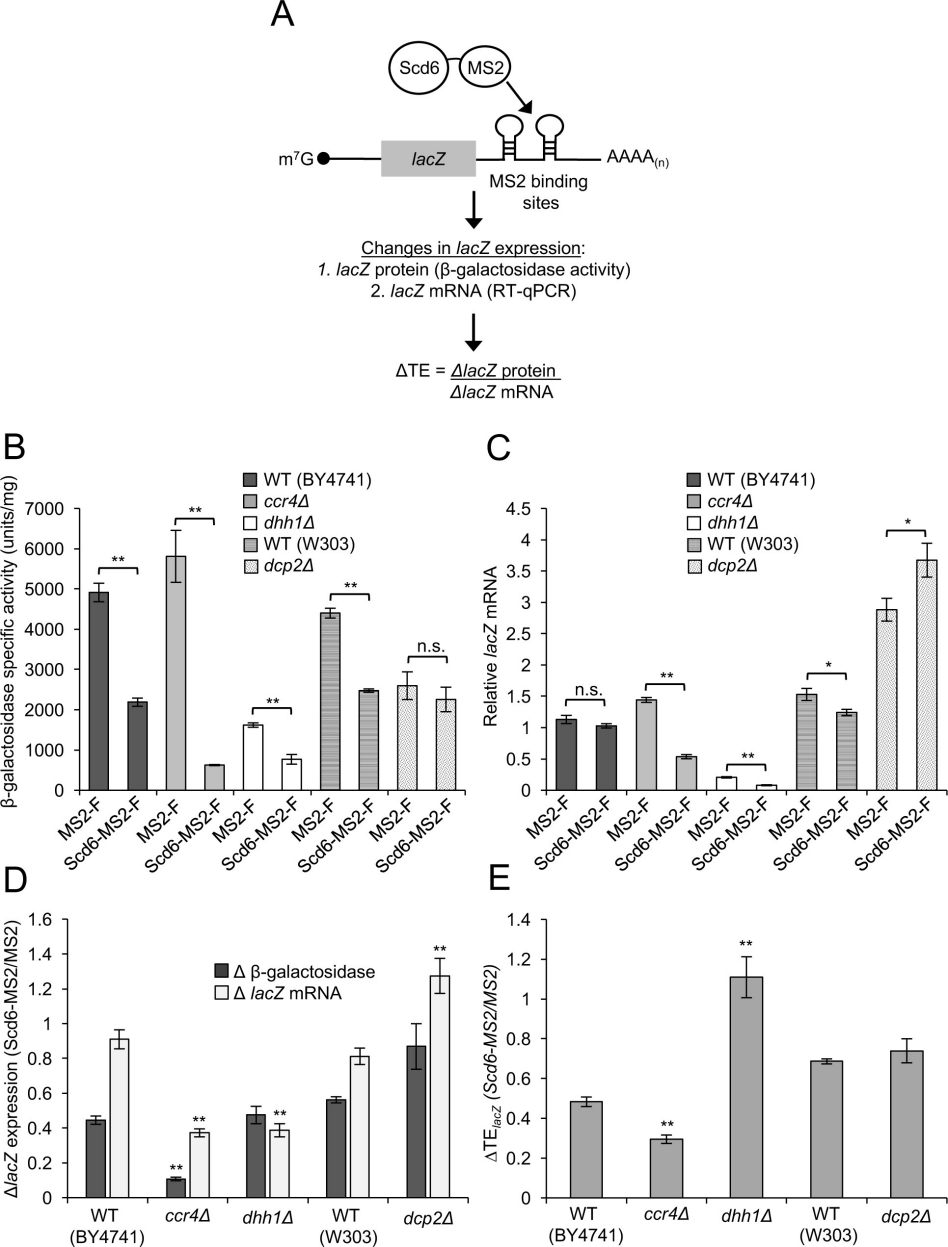
Dhh1-dependent translational repression of *lacZ* reporter mRNA by tethered Scd6-MS2-F *in vivo*. **(A)** Schema of the MS2CP tethering system, as in Fig 1A, for a *lacZ* versus *GFP* reporter mRNA. **(B-C)** Transformants of WT strains BY4741 or HFY114 (W303 background), as indicated, and *ccr4Δ* (387), *dhh1Δ* (3858), or *dcp2Δ* (CFY1016) strains, containing the MS2-F or Scd6-MS2-F expression plasmids from Fig 1 and *lacZ* reporter plasmid (pQZ131), were cultured as in Fig 1B. β-galactosidase activities (in units of nanomoles of o-nitrophenyl-b-D-galactopyranoside cleaved per min per mg) were measured in WCEs (B) and *lacZ* mRNA was quantified by RT-qPCR (C). The *ccr4Δ* and *dhh1Δ* strains are isogenic to BY4741; the *dcp2Δ* mutant is isogenic to HFY114. Mean values (± S.E.M.s) were determined from at least three biological replicates. Calculations of S.E.M.s for changes in mean ratios of β-galactosidase activity and *lacZ* mRNA expression shown in (D), and determination of P-values from significance testing of differences in mean values in (B-E) using an unpaired Student’s t-test, were conducted as described in the supporting file S1 Text. P-values are summarized as: **, P <0.01; *, P <0.05; n.s., not significant.

To examine further whether tethered Scd6-MS2-F can repress translation of reporter mRNA independently of Ccr4, we utilized an alternative reporter mRNA, in which the bacterial *lacZ* gene, encoding β-galactosidase, replaced the *GFP* coding sequences (Fig 5A), and assays of β-galactosidase activity in cell extracts replaced Western analysis for quantifying reporter protein expression. As observed with the *GFP* reporter, tethering Scd6-MS2-F conferred an ~2- to 2.5-fold repression of β-galactosidase activity compared to that measured with MS2-F alone in the two different WT strains described above (Fig 5B, Scd6-MS-F vs. MS2-F in WT(BY4741) (P<0.0001) and WT(W303) (P = 0.0005); Fig 5D, grey bars, WT strains). By contrast, β-galactosidase expression on tethering Npl3-MS2-F or Sbp1-MS2-F was indistinguishable from that observed for the corresponding MS2-only controls, or with empty vector in WT cells (S5C Fig). Thus, tethering Scd6-MS2-F, but not the corresponding Npl3 or Sbp1 fusions, confers similar repression of protein expressed from *GFP* and *lacZ* reporters. Repression of the *lacZ* reporter by Scd6-MS2-F was intact in an *scd6Δ* strain (S5A Fig; P<0.0001), ruling out a contribution of native Scd6 to the function of tethered Scd6-MS2-F. Expression of β-galactosidase activity from heterologous *GCN4-lacZ* or *GAL1-lacZ* reporters lacking MS2 binding sites was indistinguishable in cells expressing Scd6-MS-F or MS2–F (S5B Fig), indicating that repression by Scd6-MS-F requires its tethering to *lacZ* mRNA.

Interestingly, *lacZ* reporter mRNA abundance was not significantly altered, or reduced by only ~20%, by tethered Scd6-MS2-F in the two different WT strains (Fig 5C, Scd6-MS-F vs. MS2-F in WT(BY4741) and WT(W303) (P = 0.02); Fig 5D, Δ*lacZ* mRNA, WT strains). As a consequence of greater repression of protein versus mRNA expression in WT cells (Fig 5D, WT strains, grey vs. white bars), tethering Scd6-MS2-F reduced the TE of *lacZ* reporter mRNA by ~30–50% compared to MS2-F alone in the WT strains (Fig 5E, WT strains). Thus, unlike our findings for the *GFP* reporter (Fig 1F, ΔTE_*GFP*_), the ability of tethered Scd6-MS2-F to repress translation of the *lacZ* reporter was observable in WT cells containing Dcp2.

Tethering Scd6-MS2-F in *ccr4Δ* cells conferred a dramatic ~9-fold reduction in β-galactosidase expression, and also a ~2.7-fold decrease in *lacZ* mRNA expression, in comparison to MS2-F alone (Fig 5B and 5C, Scd6-MS-F vs. MS2-F, *ccr4Δ*; P = 0.001 (panel B), P<0.0001 (panel C). The reductions in β-galactosidase expression and *lacZ* mRNA on tethering Scd6-MS-F were both greater in *ccr4Δ* versus WT(BY4741) cells (Fig 5D, grey and white bars, *ccr4Δ* vs. WT(BY4741); P = <0.0001 (grey), P = 0.0005 (white). However, the reduction in β-galactosidase was relatively larger, yielding an ~3-fold reduction in TE attributable to tethered Scd6-MS2-F, which exceeds the ~2-fold reduction in TE found in WT cells (Fig 5E, *ccr4Δ*; P = 0.002). Thus, eliminating Ccr4 enhances the repression of both mRNA abundance and translational efficiency of the *lacZ* reporter by tethered Scd6-MS2-F, as observed above for the *GFP* reporter (Fig 4C). The finding that tethered Scd6-MS2-F yields a larger reduction in *GFP* versus *lacZ* reporter mRNA abundance in WT cells might be explained by proposing that another step in mRNA turnover that is not accelerated by tethered Scd6-MS-F (eg. exonucleolytic degradation by Xrn1 or deadenylation) is more rate-limiting than decapping for the *lacZ* reporter.

Compared to the levels observed in the isogenic WT strain, the levels of the *lacZ* reporter mRNA and expression of β-galactosidase were substantially reduced in *dhh1Δ* cells, even in the presence of empty vector or MS2–F (S5E and S5F Fig, vector and MS2-F, grey vs. white bars). Expression of an unrelated *GAL1-lacZ* fusion was also diminished by *dhh1Δ* in cells lacking any MS2 proteins, albeit to a smaller degree (S5D Fig). While these effects of *dhh1Δ* complicated our analysis, we nevertheless obtained results consistent with the earlier conclusion that Dhh1 is required for translational repression by tethered Scd6-MS2-F. Despite the general reductions in *lacZ* mRNA levels in *dhh1Δ* cells, Scd6-MS2-F conferred ~2.5-fold lower mRNA levels versus MS2-F alone in this mutant (Fig 5C, Scd6-MS2-F vs. MS2-F, *dhh1Δ*; P = 0.0001). Thus, eliminating *DHH1* uncovers a reduction in *lacZ* mRNA abundance on tethering Scd6-MS2-F not observed in the isogenic WT strain (Fig 5D, white bars, *dhh1Δ* vs. WT; P = 0.0002). Expression of β-galactosidase in *dhh1Δ* cells also showed an ~2-fold reduction on tethering Scd6-MS2-F versus MS2–F (Fig 5B, Scd6-MS2-F vs. MS2-F, *dhh1Δ*; P<0.0001), as in the WT strain (Fig 5D, white grey bars, *dhh1Δ* vs WT). Because tethered Scd6-MS2-F reduced both reporter mRNA and reporter protein by ~60% compared to MS2-F alone (Fig 5D, *dhh1Δ*), it did not confer any reduction in TE of *lacZ* mRNA, in contrast to the ~50% reduction in TE observed in WT cells (Fig 5E, *dhh1Δ* vs. WT; P = 0.001), which is consistent with Dhh1 being required for translational repression of the *lacZ* reporter.

Deletion of *DCP2* led to an ~2.5-fold increase in *lacZ* mRNA levels in the presence of MS2-F alone (Fig 5C, MS2-F, *dcp2Δ* vs. WT(W303)), which also occurred in the presence of vector alone and, hence, is not a consequence of tethering (S5G Fig). Tethering Scd6-MS2-F evoked an ~27% additional increase in *lacZ* reporter mRNA versus tethering MS2-F in *dcp2Δ* cells (Fig 5C, Scd6-MS2-F vs. MS2-F, *dcp2Δ*; P = 0.03), eliminating the small reduction in *lacZ* mRNA abundance on tethering Scd6-MS2-F in the isogenic WT strain noted above (Fig 5C, Scd6-MS2-F vs. MS2-F, WT; Fig 5D, white bars, *dcp2Δ* vs. WT; P = 0.001). The fact that the ~1.3-fold increase in *lacZ* mRNA conferred by tethered Scd6-MS2-F versus MS2-F in *dcp2Δ* cells is smaller than the corresponding ~2-fold increase in *GFP* mRNA abundance conferred by tethered Scd6-MS2-F in *dcp2Δ* versus WT cells shown above (Fig 2C, black vs. dark grey bars) might reflect that tethering Scd6-MS2-F confers a much smaller reduction in *lacZ* mRNA (~1.1-fold) versus *GFP* mRNA abundance (~2.6-fold) in WT cells (Figs 5D vs. 2D, WT cells, white bars). Coupling the small increase in *lacZ* mRNA abundance with a slight reduction in β*-*galactosidase expressed on tethering Scd6-MS2-F versus MS2-F in *dcp2Δ* cells (Fig 5B and 5C, Scd6-MS2-F vs. MS2-F, *dcp2Δ*), results in an ~30% reduction in TE compared to tethering MS2-F alone, which is indistinguishable from that observed in the isogenic WT strain (Fig 5E, *dcp2Δ* vs. WT(W303)). These findings are consistent with our conclusion above that Dcp2 is dispensable for translational repression by tethered Scd6-MS2-F.

In summary, tethering Scd6-MS2-F to the *lacZ* reporter confers a decrease in translational efficiency that appears to be dependent on Dhh1, independent of Dcp2, and dampened by Ccr4; and Ccr4 also diminishes the repression of *lacZ* mRNA abundance by tethered Scd6-MS2-F. All of these observations are in agreement with our findings for the *GFP* reporter. Unlike our results for the *GFP* reporter, where deleting *DCP2* reduced the TE on tethering Scd6-MS2–F (Fig 2E, cols. 1–2), *dcp2Δ* had little effect on TE of the *lacZ* reporter because it did not substantially reduce the apparent degradation of *lacZ* mRNA by tethered Scd6-MS2-F, conferring only a small increase in *lacZ* mRNA abundance (Fig 5D, WT vs. *dcp2Δ*, white bars.)

### The LSm but not the RGG domain of Scd6 is required for repression of reporter expression by tethered Scd6-MS2-F

**Fig 6:**
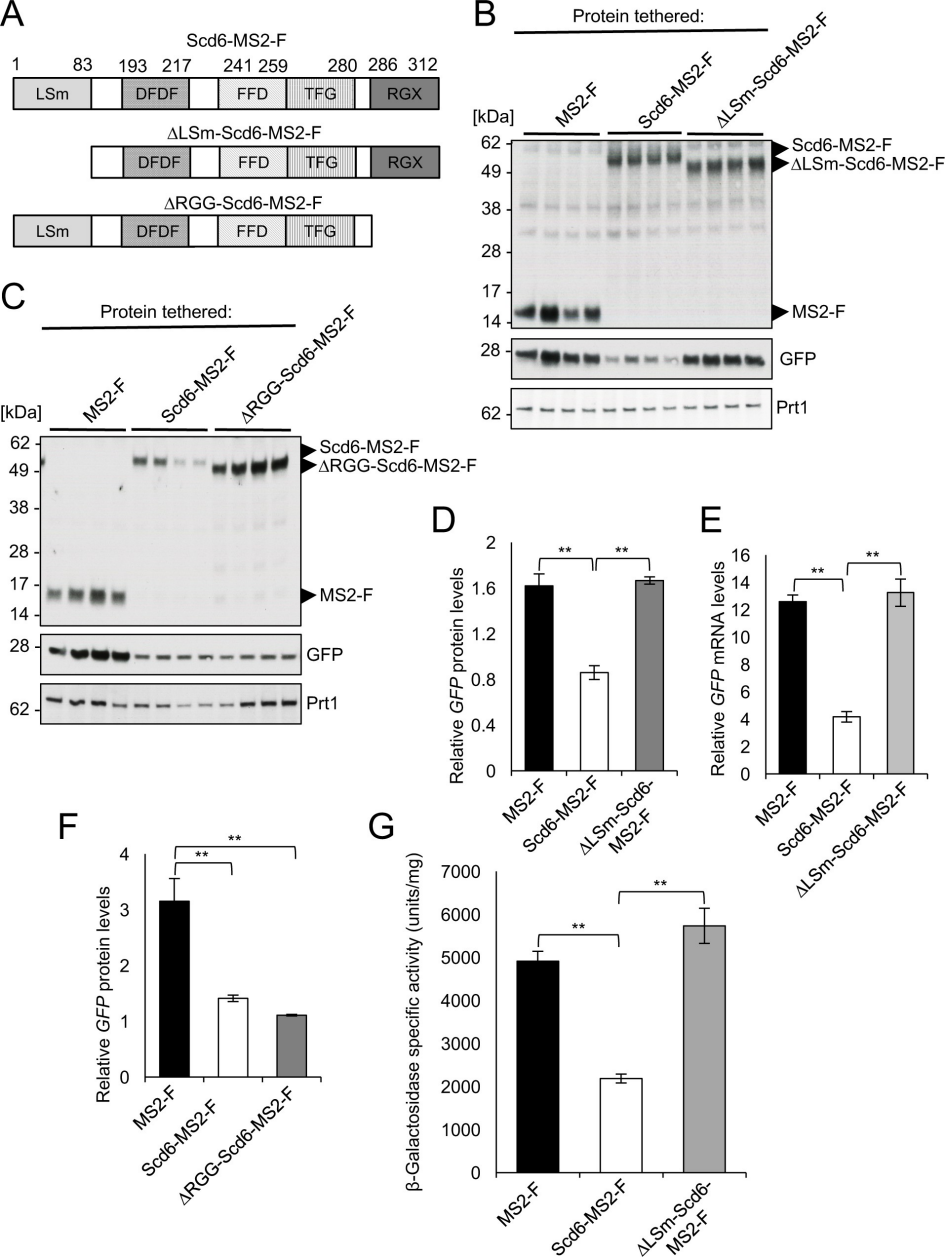
Evidence that the conserved N-terminal LSm domain is essential for translational repression and stimulation of mRNA decay by tethered Scd6-MS2-F *in vivo*. **(A)** Diagrams representing the domain organization of full-length Scd6 (Scd6-MS2-F) or variants lacking amino acids 1–83 at its N-terminus (ΔLSm-Scd6-MS2-F) or amino acids 286–312 at its C-terminus (ΔRGG-Scd6-MS2-F), present in the corresponding fusions to MS2-F. MS2 and FLAG tags are not depicted. **(B-F)** Transformants of WT strain BY4741 containing the *GFP* reporter plasmid pJC429 and expression plasmids for MS2-F (pQZ130) and Scd6-MS2-F (pQZ127), and either ΔLSm-Scd6-MS2-F (pQZ139) (B, D-E) or ΔRGG-Scd6-MS2-F (pQZ142) (C & F) were analyzed for *GFP* protein (B-D & F) and mRNA (E) expression as in Fig 1B–1D. **(G)** Transformants of WT strain BY4741 harboring the *lacZ* reporter plasmid pQZ131 and the expression plasmids for MS2-F, Scd6-MS2-F, or ΔLSm-Scd6-MS2-F used in (B) were analyzed for β-galactosidase expression as in Fig 5B. Mean values (± S.E.M.s) were determined from at least four biological replicates. Determination of P-values from significance testing of differences in mean values in (D-G) using an unpaired Student’s t-test, were conducted as described in the supporting file S1 Text. P-values are summarized as: **, P <0.01; *, P <0.05.

Scd6 interacts with eIF4G via the C-terminal region of Scd6 containing the RGG domain [22]. Interaction partners of the N-terminal LSm domain of Scd6 are unknown; however, the LSm domains in Scd6 homologs from different species mediate binding to Dcp2 in *S*. *pombe* [35], both Dcp1 and the translational repressor CUP in *D*. *melanogaster* [15], and decapping activators and translational repressors 4E-T and EDC4 in humans [36]. We examined the importance of the LSm and RGG domains of Scd6 for reporter mRNA repression by truncating the Scd6-MS2-F fusion at the N- or C-terminal ends to remove these domains individually (Fig 6A). Eliminating the LSm domain completely abrogated repression of both protein and mRNA expressed from the *GFP* reporter by tethered Scd6-MS2–F (Fig 6D and 6E, white vs. grey bars; P = 0.0001 (D), P<0.0001 (E) for ΔLSm-Scd6-MS2-F vs. Scd6-MS2-F), without detectably altering expression of the Scd6-MS2-F fusion protein (Fig 6B, upper blot, lanes 5–12). The fact that no reduction in *GFP* mRNA occurs on tethering the ΔLSm-Scd6-MS2-F variant implies that the LSm domain is required for accelerated mRNA turnover conferred by WT tethered Scd6-MS2-F. Because there is no reduction in *GFP* protein expression on tethering the ΔLSm-Scd6-MS2-F, despite high levels of the *GFP* reporter mRNA, we can also infer that translational repression of *GFP* mRNA is eliminated by removing the LSm domain. Repression of β-galactosidase expression from the *lacZ* reporter was also abolished by removing the LSm domain from Scd6-MS2–F (Fig 6G, white vs. grey bars; P<0.0001 for ΔLSm-Scd6-MS2-F vs. Scd6-MS2-F). Because there is little or no reduction in *lacZ* mRNA abundance on tethering WT Scd6-MS2-F, it seems likely that translational repression is abrogated by removing the LSm domain for this reporter mRNA as well. (However, we cannot discard the unlikely possibility that tethering ΔLSm-Scd6-MS2-F would substantially increase the abundance of *lacZ* mRNA and thereby mask efficient translational repression by the ΔLSm variant.)

In contrast to our findings on removing the LSm domain, eliminating the RGG domain from Scd6-MS2-F had no apparent effect on repression of the *GFP* reporter by Scd6-MS2–F (Fig 6C, GFP blot; Fig 6F, black vs. grey; P = 0.002), although an apparent increase in expression of the ΔRGG-Scd6-MS2-F versus WT Scd6-MS2-F fusion (Fig 6C, upper blot, lanes 5–12) might have obscured a reduced efficiency of reporter repression for the ΔRGG variant. These findings indicate that the LSm domain, and most likely interactions it mediates with effector proteins, is required for both enhanced degradation of *GFP* reporter mRNA and translational repression of both reporters by tethered Scd6-MS2-F. By contrast, interaction of Scd6-MS2-F with eIF4G via the Scd6 RGG domain might be dispensable for translational repression when Scd6 is tethered tightly to the mRNA; although we cannot eliminate the possibility that translational repression is impaired by the ΔRGG truncation and that the efficient repression of *GFP* reporter protein expression conferred by ΔRGG-Scd6-MS2-F occurs exclusively from accelerated mRNA turnover.

### Scd6 and Dhh1 cooperate in regulating mRNA abundance and translational efficiencies of particular native mRNAs in vivo

To determine whether Scd6 and Dhh1 participate in regulating the abundance or translation of native yeast mRNAs, we conducted ribosome footprint profiling and RNA-Seq analyses on the WT, *dcp2Δ*, *scd6Δ* and *dhh1Δ* strains in the W303 genetic background, cultured in rich (YPD) medium. We also analyzed isogenic *dcp2Δscd6Δ* and *dcp2Δdhh1Δ* double mutants, anticipating that changes in translational efficiency might be more evident in the absence of mRNA decapping by Dcp2. Independent RNA-Seq analysis was also conducted in parallel on two isogenic *scd6Δ* strains, an additional isogenic *dhh1Δ* strain and isogenic mutants lacking the Dcp2-decapping activators Pat1 and Lsm1 [4] to determine whether Pat1 and Lsm1 contribute to Scd6-mediated repression of mRNA abundance. The results of biological replicates were highly correlated for both ribosome-protected fragments (RPFs) and mRNA sequences for all strains analyzed (S6A–S6L Fig).

**Fig 7:**
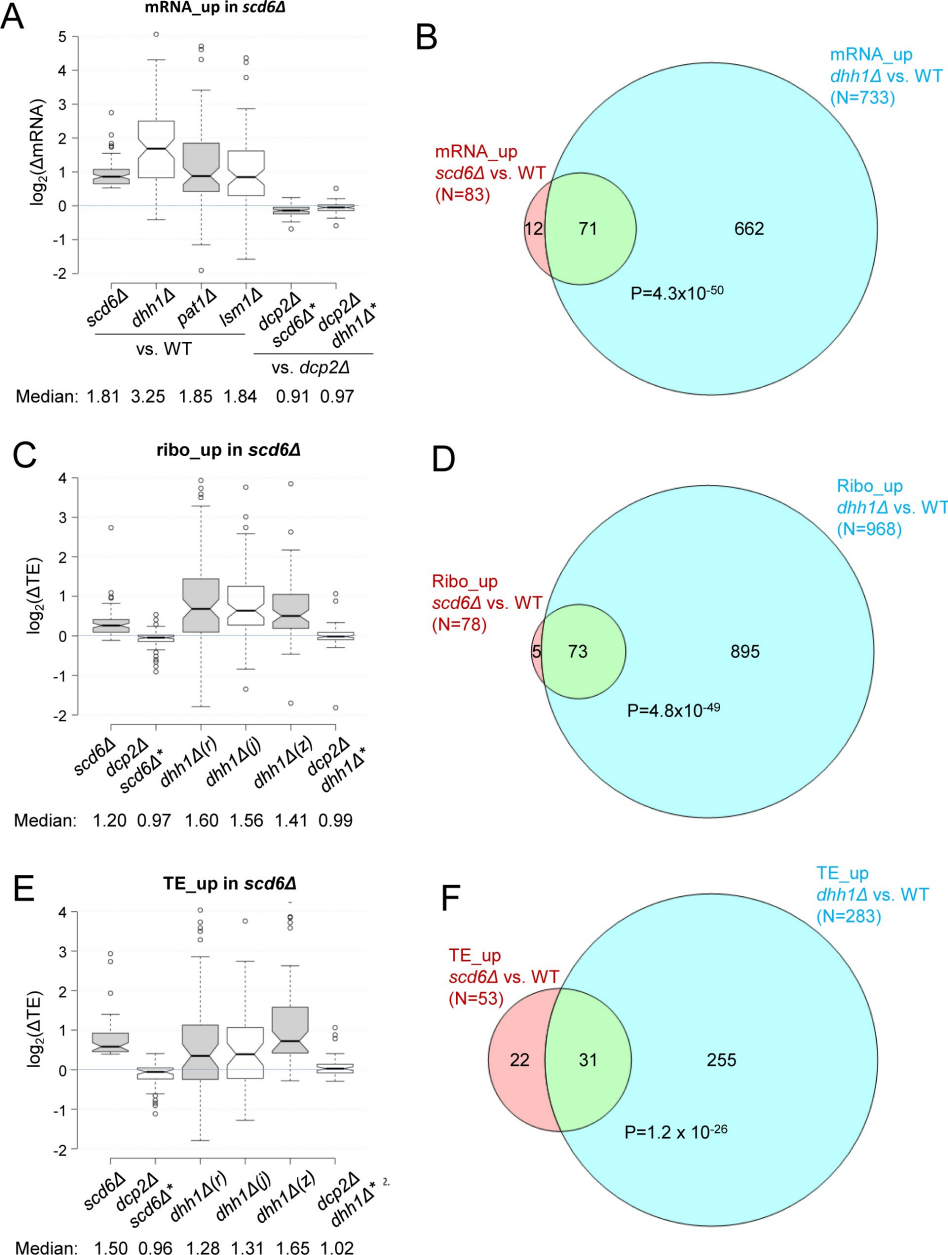
Scd6 and Dhh1 cooperate in repressing mRNA abundance and translational efficiencies of a subset of native mRNAs. **(A)** Notched box-plots of log_2_ mRNA changes (ΔmRNA) in the indicated mutants for 83 mRNAs exhibiting ≥1.4-fold increased mRNA abundance in *scd6Δ* versus WT cells (at FDR<0.01). The 83 mRNAs were identified by RNA-Seq analysis of *scd6Δ* strains (SYY2352 and SYY2353) and isogenic WT strain (HFY114); and the RNA changes shown for the mutants were determined by RNA-Seq analysis of isogenic strains of the indicated genotypes: *dhh1Δ* (SYY2686), *pat1Δ* (SYY2674), *lsm1Δ* (SYY2680), all compared to WT strain (HFY114); and *dcp2Δscd6Δ* (FZY843) and *dcp2Δdhh1Δ* (QZY128) compared to the *dcp2Δ* strain (CFY1016). (In this and all subsequent plots, the double mutants carry an (*) to indicate that their values have been compared to the *dcp2Δ* single mutant rather than to WT.) Non-overlapping notches indicate that the true medians of the two groups are different with a 95% confidence level. **(B)** Overlap between 83 mRNAs exhibiting ≥1.4-fold increased mRNA abundance in *scd6Δ* (SYY2352 and SYY2353) vs. WT (HFY114) cells and 733 mRNAs similarly up-regulated in *dhh1Δ* cells (QZY126), both at FDR<0.01. **(C)** Notched box-plots of log_2_ TE changes (ΔTE) in the indicated mutants for 78 mRNAs exhibiting ≥1.33-fold increased ribosome abundance across the CDS in *scd6Δ* versus WT cells (at P≤0.01). The 78 mRNAs were identified by ribosome profiling of *scd6Δ* strain (SYY2353); and TE changes were calculated from ribosome profiling/RNA-seq data conducted on this *scd6Δ* strain, *dhh1Δ* strain (QZY126, *dhh1Δ(z)*), and WT strain (HFY114); and on the *dcp2Δscd6Δ*, *dcp2Δdhh1Δ* and *dcp2Δ* strains mentioned in (A). TE changes for the *dhh1Δ(r)* and *dhh1Δ(j)* strains in the BY4743 background were calculated from published ribosome profiling/RNA-Seq data from Radhakrishnan et al. [10] and Jungfleisch et al. [37], respectively. **(D)** Overlap between 78 mRNAs exhibiting ≥1.33-fold increased ribosome occupancy (p<0.01) in *scd6Δ* (SYY2353) vs. WT (HFY114) cells and 968 mRNAs similarly up-regulated by ≥1.4-fold (FDR<0.01) in *dhh1Δ* cells (QZY126). **(E)** Notched box-plots of log_2_(ΔTE) values in the indicated mutants for 53 mRNAs exhibiting ≥1.33-fold increased TE in *scd6Δ* versus WT cells (at P≤0.1), identified by ribosome profiling/RNA-Seq analysis of *scd6Δ* (SYY2353) and WT (HFY114) strains. The TE changes were calculated from the data sets described in (B). **(F)** Overlap between 53 mRNAs exhibiting ≥1.33-fold increased TE (p<0.10) in *scd6Δ* (SYY2353) vs. WT (HFY114) cells and 283 mRNAs similarly up-regulated by ≥1.33-fold (FDR<0.10) in *dhh1Δ* cells (QZY126). P-values in (B-F) were assigned based on the hypergeometric distribution. For the boxplots in panels A, C, & E, the changes in mRNA abundance or TE for the relevant group of mRNAs found in each of the indicated mutants were plotted irrespective of whether the changes exhibit statistical significance in that mutant, to allow a coherent comparison of the behavior of the complete cohort of mRNAs across the entire panel of mutants. Statistical significance is evaluated for differences in the median changes found in the different mutants, with non-overlapping notches indicating with 95% confidence that the median changes found for two mutants differ from one another.

RNA-Seq analysis of WT and *scd6Δ* strains identified 83 mRNAs whose abundance was significantly up-regulated in the mutant by ≥1.4-fold at an FDR of <0.01, with a median fold-change (FC) compared to WT of ~1.8, which is significantly higher than the median FC for all mRNAs (which is 1.0 (log_2_(ΔmRNA) = 0)) owing to normalization for equal RNA read numbers for all genes in each strain) (Fig 7A, *scd6Δ*). Interestingly, this group of mRNAs also displayed significantly increased abundance in the isogenic *dhh1Δ*, *pat1Δ*, and *lsm1Δ* strains, with median FCs of ~3.3, ~1.9, and ~1.8, respectively (Fig 7A, cols. 2–4). Consistent with the latter, most of the 83 mRNAs up-regulated in *scd6Δ* cells are a subset of the larger group of 733 mRNAs elevated to the same degree in *dhh1Δ* cells (Fig 7B). Importantly however, derepression of these 83 mRNAs was not observed on deleting *SCD6* or *DHH1* in the strain lacking *DCP2*, ie. when comparing the *dcp2Δscd6Δ* and *dcp2Δdhh1Δ* double mutants to the *dcp2Δ* single mutant (Fig 7A, rows 5–6). These findings are consistent with the notions that: (i) Scd6 accelerates degradation of a subset of native mRNAs, (ii) that Dhh1, Pat1, and Lsm1 all participate in this down-regulation of mRNA abundance, and (iii) that the decapping enzyme subunit Dcp2 is required for both Scd6- and Dhh1-enhanced degradation of the set of Scd6-targeted mRNAs.

We obtained complementary results for a group of 346 mRNAs whose abundance was significantly increased (at FDR<0.01) in an isogenic *dhh1Δ* mutant, exhibiting an ~3-fold increase in median mRNA abundance in *dhh1Δ* vs. WT cells (S7A Fig, *dhh1Δ(z)*), and also showing ~1.9-fold and ~1.8-fold increases in two published RNA-seq datasets for a *dhh1Δ* mutant in the BY4741 background [10, 37] (S7A Fig, *dhh1Δ(r)* and *dhh1Δ(j)*). This group of Dhh1 down-regulated mRNAs also displays a slight, but statistically significant, ~15% derepression in the *scd6Δ* mutant (S7A Fig, *scd6Δ*). Supporting these findings, hierarchical clustering analysis of expression changes for all mRNAs revealed that a large proportion of genes exhibit altered mRNA levels in the same direction in response to *scd6Δ* or *dhh1Δ* (S7B Fig), which is particularly evident for the mRNAs showing the largest fold-changes in *dhh1Δ* vs. WT cells (S7C Fig), with the magnitudes of these changes being generally greater in *dhh1Δ* vs. *scd6Δ* cells (S7B and S7C Fig). These results suggest that Scd6 contributes appreciably to Dhh1-enhanced degradation of a large fraction of the mRNAs whose abundance is repressed by Dhh1. As observed for the Scd6 down-regulated mRNAs, the derepression of mRNA levels for the group of 346 Dhh1-repressed mRNAs conferred by either *scd6Δ* or *dhh1Δ* was eliminated when these mutations were made in cells lacking *DCP2* (S7A Fig, cf. cols. 1–2 and 5–6), indicating that Dcp2 is required for robust Dhh1-mediated mRNA turnover.

The two groups of 83 and 346 mRNAs whose abundance is derepressed in *scd6Δ* (Fig 7A) or *dhh1Δ* cells (S7A Fig), respectively, were interrogated next for changes in translation efficiency (TE) in different mutants by combining the results of ribosome profiling and RNA-Seq experiments conducted on the same cell cultures. The TE of each mRNA was calculated as the sum of RPFs divided by the sum of RNA reads across the CDS, and the change in TE (ΔTE) was calculated as the ratio of TE in mutant versus WT cells. (Because RPFs and mRNA reads for each gene are normalized to RPF or mRNA reads for all genes in each strain, the ΔTE for each gene is determined relative to the median ΔTE for all genes, which is ~1.0). Both groups of mRNAs showing derepression of mRNA abundance in *scd6Δ* or *dhh1Δ* cells also exhibited modest increases in median TE in our *dhh1Δ* mutant of ~12–15% (S7D and S7E Fig, col. 5 in each panel). Comparable or somewhat greater increases in TE were identified in the two published ribosome profiling/RNA-seq datasets for a *dhh1Δ* mutant in the BY4741 background [10, 37] (S7D and S7E Fig, cols. 3–4 in each panel); whereas the *scd6Δ* mutation increased the median TE for these groups of mRNAs by only ~5% (S7D and S7E Fig, col. 1). Despite the modest TE increases for these groups of mRNAs conferred by *dhh1Δ*, it is noteworthy that these changes were not observed on comparing the *dcp2Δdhh1Δ* double mutant to the *dcp2Δ* single mutant (S7D and S7E Fig, cols. 5–6 in each panel), providing genetic evidence that the TE changes are genuine, and indicating that translational repression by Dhh1 is dependent on Dcp2.

**Fig 8:**
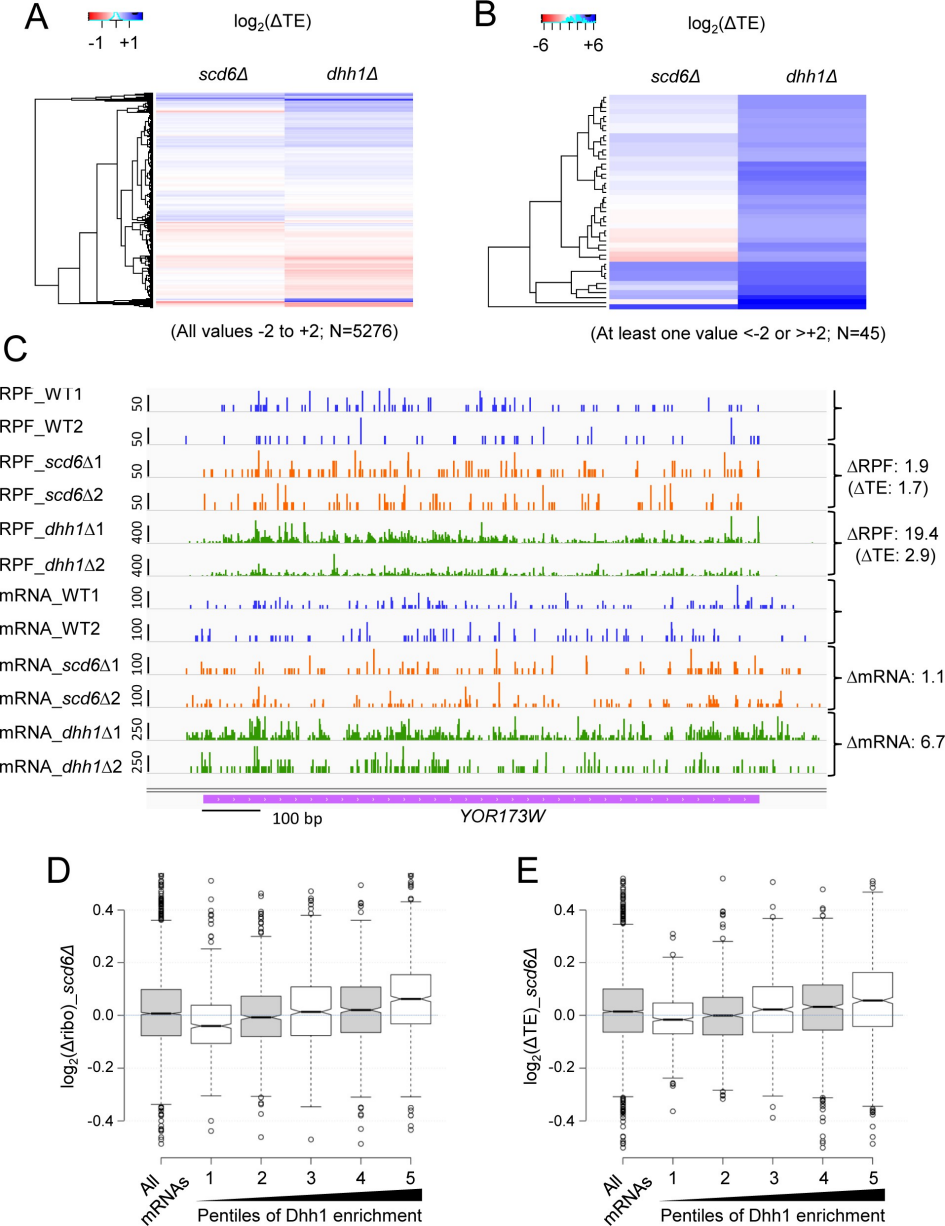
Scd6 and Dhh1 cooperate in repressing translational efficiencies of a subset of native mRNAs in a manner associated with elevated Dhh1 occupancies. **(A-B)** Hierachical clustering analysis conducted with the R heatmap.2 function from the R gplots library, using the default “hclust” hierarchical clustering algorithm, using ribosome profiling data from *scd6Δ* (SYY2353), *dhh1Δ* (QZY126, *dhh1Δ(z)*), and WT (HFY114) strains. Approximately 50 genes were removed for which no data were available in one of the strains, or where the log_2_(ΔTE) value was >4 or <-4 in one of the mutant vs. WT comparisons, after which separate clustering analysis was performed on two sets of mRNAs in which all log_2_(ΔTE) values fell between -2 or +2 (panel A, 5276 mRNAs), or in which the log_2_(ΔTE) value in one of the mutants was < -2 or > +2 (panel B, 45 mRNAs). The color key for log_2_(ΔTE) values is indicated above each analysis. **(C)** Exemplar gene exhibiting increased TE in both *scd6Δ* and *dhh1Δ* versus WT cells. Integrated Genomics Viewer (Broad Institute) display of ribosome-protected fragments (RPFs) and mRNA reads across the *YOR173W* gene from two biological replicates each for WT, *scd6Δ* and *dhh1Δ* strains, shown in units of rpkm (reads per 1000 million mapped reads). Position of the CDS (magenta) is at the bottom with the scale in bp; scales of rpkm for each track are on the left, and calculated ΔRPF, ΔmRNA and ΔTE values between each mutant and WT are on the right. **(D-E)** Boxplot analysis of changes in ribosome occupancy or TE versus Dhh1 occupancy. Dhh1 RIP-seq enrichment values from Miller et al (2018) were equally divided into five pentiles of 739 genes from lowest to highest values and plotted against the log_2_(Δribo) values (D) or log_2_(ΔTE) values (E) determined by ribosome profiling analysis of *scd6Δ* strain SYY2353 and WT strain HFY114. The Pearson correlation coefficients for the relationship between log_2_(Δribo) values (panel D) or log_2_(ΔTE) values (panel E) and Dhh1 enrichment for all mRNAs are 0.2 (P = 2 X 10^−34^) and 0.16 (P = 2 X 10^−22^), respectively.

Evidence for Dcp2-dependent translational repression of native mRNAs by Scd6 was provided by examining a group of 78 mRNAs exhibiting significantly increased ribosome occupancies in the *scd6Δ* mutant, and for a second group of 53 mRNAs showing the largest TE increases conferred by *scd6Δ*. In response to *scd6Δ*, both groups of mRNAs exhibit increased median TEs of ~1.2- and ~1.5-fold, respectively, in otherwise WT cells, but not in the presence of *dcp2Δ* (Fig 7C & 7E, cf. cols. 1–2 in each panel). Comparable, or somewhat greater, increases in median TE were observed for both groups of mRNAs in all three *dhh1Δ* datasets (Fig 7C & 7E, cols. 3–5), indicating that Dhh1 contributes to translational repression of a substantial proportion of the mRNAs thus repressed by Scd6. This last inference is further supported by the significant overlaps between mRNAs exhibiting increased ribosome occupancies or TEs in response to *scd6Δ* and the larger groups of mRNAs showing comparable increases in ribosome occupancy or TE in response to *dhh1Δ* (Fig 7D & 7F). Moreover, clustering analysis for all mRNAs showed that the majority of mRNAs displaying increased TEs in response to *scd6Δ* also exhibit TE increases of generally greater degree in response to *dhh1Δ*; although it is also evident that many mRNAs translationally repressed by Dhh1 are not repressed by Scd6 (Fig 8A and 8B). Once again, comparing the *dcp2Δdhh1Δ* double mutant to the *dcp2Δ* single mutant revealed that *dcp2Δ* largely suppresses the TE increases conferred by *dhh1Δ* in *DCP2* cells (Fig 7C & 7E, cf. cols. 5–6 in each panel), supporting a widespread requirement for Dcp2 in translational repression by Dhh1.

The mRNAs encoded by *YOR173W*, *YHR033W*, *YGR088W*, *YFR017C*, and *YNR034W-A* exhibit TE increases conferred by *scd6Δ*, and they respond similarly to *dhh1Δ*, while exhibiting larger increases in mRNA and TE levels in *dhh1Δ* versus *scd6Δ* cells (Fig 8C and S8A–S8D Fig). Gene-ontology (GO) analysis revealed that mRNAs whose transcript abundance was derepressed in *dhh1Δ* or *scd6Δ* cells are enriched in related functional categories of genes involved in metabolism of energy reserves and other aspects of carbohydrate metabolism; and the Dhh1-repressed mRNAs are also enriched in stress-response genes (S9A and S9B Fig). These results suggest that Scd6 and Dhh1 cooperate in repressing both mRNA abundance and translational efficiency for a discrete subset of native mRNAs in nutrient-replete medium.

To provide evidence supporting a direct role for Dhh1 in translational repression of native mRNAs by Scd6, we interrogated published results from deep-sequencing of mRNAs specifically immunoprecipitated with epitope-tagged Dhh1 (RIP-Seq) from WT cells grown on rich medium [38]. The 3686 mRNAs for which both RIP-Seq and ribosome profiling data exist were sorted into 5 equal percentiles based on Dhh1 enrichment values in RIP-seq and compared for translation changes in response to *dhh1Δ* or *scd6Δ*. This analysis revealed a direct correlation between Dhh1 enrichment values and changes in both ribosome occupancies and TEs in response to *dhh1Δ* (S10A and S10B Fig), and a similar correlation exists between Dhh1 enrichment and changes in mRNA levels in *dhh1Δ* vs. WT cells (S10C Fig), as noted previously [38]. These correlations support previous conclusions that Dhh1 binding to mRNAs is associated with accelerated mRNA decay and translational repression. Interestingly, Dhh1 enrichment values are likewise correlated with changes in ribosome occupancies and TEs in response to *scd6Δ* (Fig 8D and 8E), supporting the notion that Scd6 translational repression of many native mRNAs involves recruitment of Dhh1. Finally, we considered the possible contribution of codon optimality in dictating susceptibility of mRNAs to Scd6. Previously, low codon optimality was associated with Dhh1-mediated mRNA decay in part by demonstrating an inverse correlation between the sTAI value, a measure of overall codon optimality of the mRNA [39], and the change in mRNA abundance in *dhh1Δ* versus WT cells [10]. This correlation is also evident in our *dhh1Δ* dataset and that of Jungfleisch et al. [37] (S11B and S11C Fig), although less pronounced than observed in the data from Radhakrishnan et al. [10] (S11A Fig). A similar, modest trend was also evident for mRNA changes observed here in *scd6Δ* cells (S12A Fig), suggesting that mRNAs exhibiting Scd6-dependent mRNA degradation have a tendency to exhibit poor codon optimality. Interestingly, however, the group of 82 mRNAs whose abundance is most strongly derepressed in *scd6Δ* cells (characterized in Fig 7A) exhibit sTAI values that are somewhat higher, not lower, than the genome average value (S12B Fig), indicating that poor codon optimality is not the key determinant of Scd6-dependent mRNA turnover for the transcripts that it most strongly represses.

## Discussion

In this report we have shown that tethering an Scd6-MS2-F fusion protein to two different reporter mRNAs harboring MS2 binding sites represses reporter protein expression and, in the case of the *GFP* reporter, also reduces reporter mRNA abundance. Together with Npl3 and Sbp1, Scd6 is one of three yeast proteins containing RGG domains capable of binding to the C-terminal domain of eIF4G and repressing translation initiation in cell extracts [22]. However, we observed no effects on reporter expression on tethering Npl3 or Sbp1. The fact that tethered Scd6-MS2 reduced β-galactosidase expression without reducing *lacZ* mRNA abundance implied a reduction in translational efficiency of the *lacZ* mRNA. While this inference was not possible for the *GFP* reporter in WT cells, owing to comparable reductions in protein and mRNA expression, it was clearly indicated by the much greater repression of *GFP* protein versus *GFP* mRNA conferred by tethered Scd6-MS2 in the *ccr4Δ* mutant. Moreover, translational repression of the *GFP* reporter was revealed in the *dcp2Δ* mutant, as the reduction in *GFP* mRNA abudance was diminished while repression of *GFP* protein was maintained. Thus, we propose that tethering Scd6-MS2 evokes translational repression of both *GFP* and *lacZ* reporter mRNAs, and also degradation of the *GFP* reporter mRNA, with the latter dependent on decapping by Dcp2. Additional evidence for translational repression was provided by our finding that tethering Scd6-MS2 in *dcp2Δ* cells shifted a proportion of the *GFP* mRNA from large to smaller polysomes and monosomes, suggesting a reduced rate of translation initiation.

We implicated Dhh1 in translational repression of both *GFP* and *lacZ* mRNAs, but found it to be dispensable for the degradation of *GFP* mRNA, evoked by tethered Scd6-MS2-F. Thus, the TE values for both *GFP* and *lacZ* mRNAs were higher in *dhh1Δ* versus WT cells (Figs 2E & 5E); and also were higher in the *dhh1Δ dcp2Δ* double mutant compared to the *dcp2Δ* single mutant for the *GFP* reporter (Fig 2E). Importantly, repression of both *GFP* protein and *GFP* mRNA abundance by tethered Scd6-MS2 is absent in the *dhh1Δ dcp2Δ* double mutant, whereas *GFP* protein repression is intact in *dcp2Δ* cells, and repression of *GFP* mRNA abundance occurs in *dhh1Δ* cells (Fig 2D). These comparisons indicate that Dcp2 is required for efficient mRNA degradation while Dhh1 is required for full translational repression of *GFP* reporter mRNA. The role of Dhh1 in translational repression is further supported by our finding that tethering Dhh1 as an MS2 fusion represses expression of *GFP* protein more than *GFP* mRNA abundance in *ccr4Δ* cells, as observed previously [6]), similar to the effects of tethered Scd6-MS2–F (Fig 4A and 4B). These results are consistent with previous demonstrations of direct interactions between Scd6 and Dhh1 [23, 24]. Our finding that repression of *GFP* mRNA abundance by Scd6-MS2 remains intact in the *dhh1Δ* strain implies that Dhh1 is not required for recruitment or activation of Dcp1/Dcp2 on this reporter mRNA, which could involve instead the known direct interaction of Scd6 with Dcp2 [7, 23, 24, 27, 29].

Although we could readily observe that tethered Scd6-MS2-F reduces the TE of the *GFP* reporter mRNA in *ccr4Δ* cells, it was not possible to infer translational repression in WT cells because tethered Scd6-MS2-F repressed *GFP* reporter and protein expression almost equally. However, translational repression by tethered Scd6-MS2-F was uncovered in the *dcp2Δ* strain in which the accelerated degradation of *GFP* reporter mRNA was diminished. The ability to observe translational repression after uncoupling it from mRNA turnover in a *dcp2Δ* mutant has been reported previously for Dhh1 using the same tethering assay and *GFP* reporter employed here [6], and also in similar experiments involving the tethering of Dhh1 to reporter mRNA in mutant strains where mRNA turnover was impaired by elimination of Dcp1 or Xrn1 [34]. Together, these findings indicate that both Scd6 and Dhh1 can repress translation independently of their functions in activating mRNA decay. In addition to the genetic uncoupling of mRNA decay from translational repression accomplished in yeast, these processes have been kinetically resolved in miRNA-mediated repression in Drosophila cells [40] and zebrafish [41] by showing that translational repression precedes mRNA turnover.

Previous findings showed that tethered Dhh1-MS2 interferes with the elongation stage of translation and is associated with the presence of slowly decoded suboptimal codons in the reporter mRNA [6]. Current evidence suggests that Dhh1 can be recruited to mRNAs by slowly elongating ribosomes and triggers decapping and subsequent mRNA degradation; and that Dhh1 can also impede the progression of 80S ribosomes at suboptimal codons, at least when tethered to mRNA or overexpressed in cells [10]. These findings are ostensibly at odds with our conclusion that Dhh1 participates in translational repression by tethered Scd6-MS2-F and the results of our polysome analysis indicating that tethered Scd6-MS2 does not shift the *GFP* reporter mRNA into larger polysomes (Fig 3), which would be expected for slower elongation. Rather, tethered Scd6-MS2 appears to shift the mRNA towards smaller polysomes and possibly free mRNP (Fig 3). However, Dhh1 can bind directly to both 40S and 60S subunits [6, 10], and has been implicated in the inhibition of bulk translation initiation during carbon starvation [42] or when overexpressed in nutrient-replete cells [9]. Moreover, Dhh1 can inhibit 48S PIC assembly when added to cell extracts [9]. It has been suggested that Dhh1 can inhibit either initiation by interacting with the PIC, or elongation by binding to translating 80S ribosomes, and the relative importance of these mechanisms could vary with the mRNA, depending, for example, on the number and position of suboptimal codons [6, 10]. Although tethered Dhh1-MS2 was found to inhibit elongation on the *GFP* reporter mRNA [6], for which tethered Scd6-MS2 appears to have a relatively greater effect on initiation, perhaps the amount of Dhh1 that would be recruited to the reporter by Scd6-MS2 is lower than achieved by tethering Dhh1-MS2 itself, and may be sufficient to inhibit initiation but not elongation. As shown for Dhh1, Scd6 can also inhibit 48S PIC formation in cell extracts [7, 22] and tethered Scd6-MS2-F might work in conjunction with Dhh1 to produce a rate-limiting initiation defect on the *GFP* reporter.

We found that the LSm domain of Scd6 is indispensable for the ability of tethered Scd6-MS-F to repress *GFP* reporter mRNA and protein expression (Fig 6), implying its requirement for both translational repression by Dhh1 and decapping by Dcp2. Whereas the LSm domains of Scd6 homologs in *S*. *pombe* and *D*. *melanogaster* have been shown to interact with Dcp1 or Dcp2, the interactions with Dhh1 homologs involve the DFDF and TFG domains in the C-terminal regions of Scd6 homologs in *D*. *melanogaster* [15] and humans [36]. If the LSm domain in *S*. *cerevisiae* Scd6 likewise interacts with Dcp1 or Dcp2, this could explain the requirement for this domain in stimulating mRNA degradation by tethered Scd6-MS2-FL; however, the additional requirement of the LSm domain for translational repression presumably does not involve direct recruitment of Dhh1. Considering that the LSm domains in Scd6 homologs also bind the translational repressor proteins CUP in *Drosophila* [15] and 4E-T in humans [36], we suggest that this domain in *S*.*cerevisiae* Scd6 likewise recruits an additional repressor protein that, together with Dhh1, mediates translational inhibition by tethered Scd6-MS2-FL.

In contrast to our findings on the LSm domain of Scd6, we found that the RGG domain at the C-terminus of Scd6 was not needed for repression of the *GFP* reporter by tethered Scd6-MS-F. As interaction of the Scd6 RGG domain with eIF4G was found previously to be required for the inhibition of translation initiation by Scd6 in cell extracts [22], it is possible that this interaction interferes with the intrinsic function of the eIF4G C-terminal region in 48S PIC assembly. Alternatively, the Scd6-RGG/eIF4G interaction could serve primarily to recruit Scd6 to eIF4F-mRNP complexes for inhibition of 43S PIC recruitment, via Scd6 interactions with other components of the eIF4F-mRNP or 43S PIC, or by recruiting repressor proteins like Dhh1 or Pat1 to do so. Although our findings are more consistent with the latter possibility, an inhibitory interaction of the Scd6 RGG domain with the eIF4G C-terminus might still be crucial for translational repression on native mRNAs to which Scd6 is not tightly tethered.

Our results using the tethering assay demonstrate that Scd6 can repress both mRNA abundance and translational efficiency of specific reporter mRNAs when tethered to these mRNA targets in vivo. Using RNA-Seq and ribosome profiling we went on to provide evidence that Scd6 is involved in repressing the abundance and/or translation of a discrete set of native yeast mRNAs in cells cultured in rich medium. The abundance of a group of 83 mRNAs was significantly up-regulated in *scd6Δ* cells, and the functions of the encoded proteins are enriched in the processes of metabolism of energy reserves and other aspects of carbon metabolism. Similarly, the abundance of a group of 346 mRNAs was derepressed in *dhh1Δ* cells, which are enriched for the same functional categories, as well as in stress response functions. These findings are consistent with recent results indicating that Dhh1-occupied mRNAs are enriched for transcripts whose levels are derepressed in cells depleted of glucose or a preferred nitrogen source [38]. Importantly, the group of 83 mRNAs whose levels are elevated in *scd6Δ* cells also tend to be elevated in mutants lacking Dhh1, Pat1, or Lsm1 (Fig 7A and 7B), suggesting cooperation among these decapping activators in degradation of many native Scd6 target mRNAs. The groups of mRNAs whose abundance is derepressed in *scd6Δ* or *dhh1Δ* cells also exhibit a modest up-regulation in median TE values in *dhh1Δ* cells (S7D and S7E Fig), consistent with concerted mRNA destabilization and translational repression by Dhh1 on a subset of these mRNAs. It is possible that the observable extent of translational repression of these mRNAs is dampened by their accelerated degradation, in the manner we observed for the *GFP* reporter mRNA on tethering Scd6-MS2-F.

**Fig 9:**
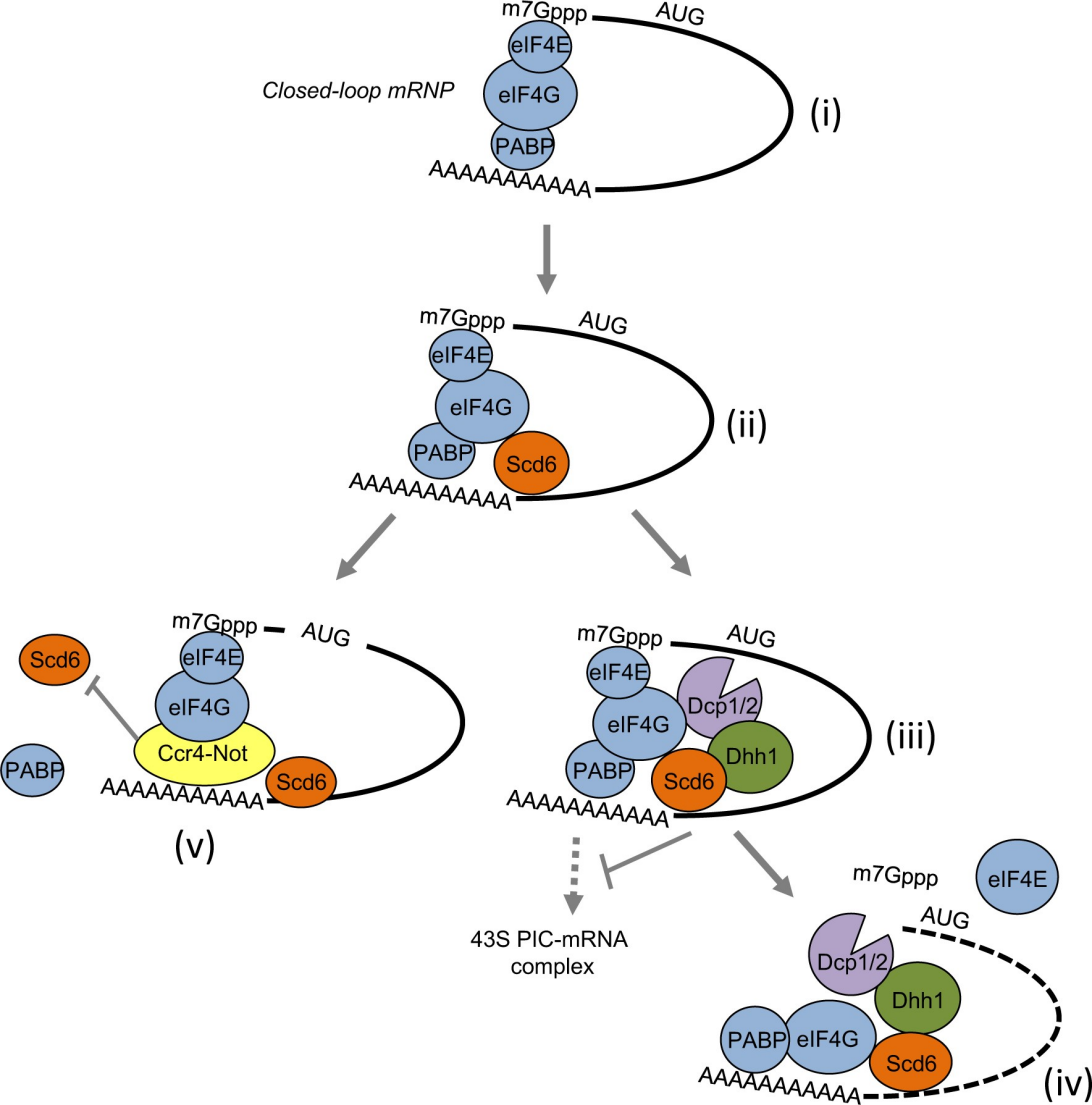
Model for Scd6-stimulated translational repression via Dhh1 and mRNA decapping by Dcp1/Dcp2, perturbed by the Ccr4/Not complex. **(i)** Closed-loop mRNP formation by mutual interactions of eIF4G with eIF4E bound to the mRNA cap and PABP bound to the poly(A) tail of mRNA, activating mRNA for translation initiation. **(ii)** Scd6 is recruited to the mRNA by binding to the C-terminus of eIF4G, but might also interact independently with the 3’UTR. **(iii)** Scd6 recruits Dhh1 and Dcp1/Dcp2 to form an inactive mRNP incapable of recruiting the 43S PIC complex near the 5’ end of the mRNA. **(iv)** Dcp1/Dcp2 decaps the mRNA, dissociating eIF4E, and exposing the 5’ end of the mRNA for subsequent exonucleolytic degradation (depicted as dotted line). **(v)** Recruitment of the Ccr4/Not complex to the mRNA interferes with Scd6-mediated translational repression and mRNA degradation.

For two additional groups of mRNAs exhibiting the largest increases in ribosome occupancy or TE in *scd6Δ* cells, we again observed a contribution of Dhh1 to translational repression (Fig 7C–7F), similar or even greater in magnitude to that of Scd6 for these groups of mRNAs (Fig 7C & 7E). Broad cooperation between Scd6 and Dhh1 in translational control was also evident in genome-wide comparisons of TE changes in *scd6Δ* vs. *dhh1Δ* cells (Fig 8A and 8B). Moreover, we found that Dhh1 occupancy is correlated with increased translation and increased TE in *scd6Δ* cells (Fig 8D and 8E) as well as in *dhh1Δ* cells (Fig 9A and 9B). These findings support our conclusion reached from tethering assays that translational repression by Scd6 involves Dhh1.

Interestingly, our analyses of double mutants lacking Dcp2 in addition to Scd6 or Dhh1 indicated that translational repression, as well as mRNA degradation, mediated by Scd6 or Dhh1 is highly dependent on Dcp2 for most native mRNAs regulated by these proteins. Dcp2-dependence was expected for repression of mRNA levels, as decapping is an established prelude to mRNA degradation by the 5’-3’ exonuclease Xrn1 in yeast [4]. We did not anticipate a requirement for Dcp2 in translational repression, however, as translational repression of the *GFP* reporter by both tethered Scd6-MS-F (Fig 2) and Dhh1-MS [6] was uncovered in *dcp2Δ* cells by the reduced *GFP* mRNA turnover, rather than being diminished. One way to account for this discrepancy is to propose that, on native mRNAs targeted by Scd6 or Dhh1, Dcp2 is required for stable assembly of a translation repression complex capable of impeding 43S PIC association (Fig 9, (iii)), in addition to decapping mRNA to enhance degradation (Fig 9, (iv)), and this contribution of Dcp2 to translational inhibition is bypassed by artificially increasing the occupancies of Scd6 or Dhh1 on the mRNAs via tethering. It is also possible that a broad effect of *dcp2Δ* in increasing the abundance of many capped mRNAs, possibly with shortened poly(A) tails, indirectly diminishes translational repression by Dhh1 or Scd6 binding to target mRNAs. Although the underlying mechanism for the role of Dcp2 in translational repression of native mRNAs remains to be determined, the fact that *dcp2Δ* completely suppressed the increased mRNA levels and TE values conferred by *scd6Δ* or *dhh1Δ* (Fig 7A, 7C and 7E) provides genetic evidence that, while modest in magnitude for *scd6Δ*, these changes are physiologically relevant for the affected mRNAs.

An unexpected finding from the tethering assays is that Scd6-MS2 binding conferred only a small decrease in *lacZ* reporter mRNA levels, which was limited to one genetic background (Fig 5C), but a marked reduction in abundance of *GFP* mRNA (Figs 1 and 2). We considered that these different responses of the *GFP* and *lacZ* reporters to Scd6-MS2-F tethering might arise from differences in codon optimality. However, the sTAI values for the *GFP* and *lacZ* coding sequences, 0.37 and 0.30, respectively, are both within one standard deviation of the mean sTAI value for all yeast genes of 0.35 [10]. Given the negative correlation between sTAI values and change in mRNA abundance conferred by *dhh1Δ* for native mRNAs [10] (S11A Fig), it might be expected that *lacZ* mRNA (in the absence of tethered Scd6-MS2-F) would show increased abundance in *dhh1Δ* vs. WT cells; however we observed the opposite (S5F Fig), and we made qualitatively similar findings for a heterologous *GAL1-lacZ* mRNA (S5D Fig). Thus, these *lacZ* mRNAs behave more like mRNAs with optimized codons, except that the magnitudes of their reductions in *dhh1Δ* cells (2.5 to 4.5-fold) exceed the typical response of 10–20% reduced abundance seen for native codon-optimal mRNAs [10]. In addition, one might expect that tethering Scd6-MS2 would evoke greater Dhh1-mediated reduction in mRNA abundance for the less codon-optimal *lacZ* versus more codon-optimal *GFP* mRNA [10], but again we found the opposite result. Hence, it is unlikely that differences in codon optimality underlie the different responses of these two reporters to tethered Scd6-MS2. Finally, it is noteworthy that most of the 53 mRNAs exhibiting the largest TE increases in *scd6Δ* cells do not exhibit increases in mRNA abundance (S7F Fig), indicating that Scd6 frequently decreases TE without reducing mRNA abundance of native mRNAs—as we observed for the *lacZ* reporter on tethering Scd6-MS2. On the other hand, a proportion of the 53 mRNAs do exhibit increased mRNA abundance in parallel with increased TE in *scd6Δ* cells (found in upper quartile of box plot in col. 1 of S7F Fig)—implying coupled repression of TE and mRNA abundance by Scd6—as we observed for the *GFP* reporter on tethering Scd6-MS2.

Another unexpected finding from the tethering assays was that reductions in reporter mRNA levels on tethering Scd6-MS-F are enhanced in *dhh1Δ* cells, increasing the repression ratio of *GFP* mRNA abundance (Fig 2C and 2D) and uncovering a repression of *lacZ* reporter mRNA abundance that was barely detectable in WT cells (Fig 5C and 5D). These observations might indicate that Dhh1 interferes with mRNA degradation evoked by tethered Scd6-MS2-F. This influence of Dhh1 was not seen for the five native mRNAs presented as exemplars of Scd6 translational repression (Fig 8C and S8A–S8D Fig), which all exhibit higher rather than lower mRNA levels in *dhh1Δ* cells. Moreover, increased mRNA levels in *dhh1Δ* cells holds for a large proportion of the group of 53 mRNAs whose TE was most strongly derepressed in the *scd6Δ* mutant (S7F Fig, col. 3). However, there is also a fraction of these mRNAs that do resemble the reporter mRNAs on Scd6-MS2 tethering in showing decreased mRNA abundance in *dhh1Δ* cells (S7F Fig, bottom quartile in col. 3). More work is required to understand the differing responses to Dhh1 for different Scd6 targets.

In summary, our results, in combination with previous findings on yeast Scd6 [22], support a model wherein recruitment of Scd6 to an mRNA, directed by or stabilized by its interaction with eIF4G, enables Scd6 to recruit other effectors of mRNA decapping/degradation and translational repression including, but not limited to, Dcp1/Dcp2 and Dhh1, and possibly also to interfere directly with recruitment of the 43S PIC by binding to the C-terminus of eIF4G (Fig 9, (i-iii)). Decapping by Dcp1/Dcp2 and subsequent degradation of the mRNA can proceed concurrently with translational repression (Fig 9, (iv)). Based on our findings that *ccr4Δ* enhances mRNA turnover and translational repression, we suggest that association of the Ccr4/Not complex with the mRNA, or deadenylation of the mRNA, interferes with the ability of Scd6 to associate with the mRNA or recruit decapping activators and translational repressors, thereby diminishing Scd6-enhanced mRNA degradation and translational repression in WT versus *ccr4Δ* cells. Further work will be required to determine whether Scd6 is recruited to specific mRNAs by 3’UTR sequences or RNA binding proteins unique to its mRNA targets, or whether intrinsic features of mRNAs (sequences or other binding proteins) confer a heightened sensitivity to Scd6 that would be recruited broadly to most mRNAs by eIF4G. Our identification of native mRNAs targeted by Scd6 for translational repression sets the stage for efforts to reconstitute the repressive function of Scd6 and its associated decapping activators in a purified translation system, and thereby elucidate their molecular mechanisms of translational control.

## Materials and methods

### Plasmid constructions

Plasmids employed in this study are listed in Table 1. Plasmids containing constructs encoding FLAG-tagged MS2-CP fusions to Npl3, Sbp1, and Scd6 (S13 Fig, *left*) were constructed by first PCR-amplifying *NPL3*, *SBP1* or *SCD6* respectively with their native endogenous promoter (~500 bp upstream flanking sequence) plus their coding sequence minus the stop codon, from genomic DNA of WT strain BY4741, with primers containing a gene-specific restriction site at the N-terminus, and an XhoI site and XbaI/SpeI site at the C-terminus. The following primers were used: (i) *NPL3* (forward primer with SpeI site, 5’-ACGAGGACTAGTTATCAATATGCAAATGCTCGGC-3’; reverse primer with XhoI and SpeI sites, 5’-ACGAGCACTAGTCTCGAGCCTGGTTGGTGATCTTTCACG); (ii) *SBP1* (forward primer with XbaI site, 5’-ACGAGCTCTAGATCATCGAGCGGAAAATATTG-3’; reverse primer with XhoI and XbaI sites, 5’-ACGAGCTCTAGACTCGAGTTCTTGCTTTTCTTCAGAACC-3’); (iii) *SCD6* (forward primer with SpeI site, 5’-ACGAGGACTAGTTGCTCGTAACAATCTTGG-3’; reverse primer with XhoI and SpeI sites, 5’-ACGAGGACTAGTCTCGAGAAATTCAACGTTGGAAGGAGG-3’). The amplified fragments were inserted between the XbaI/SpeI sites of YCplac111 to generate YCplac111-NPL3, YCpLac111-SBP1, or YCpLac111-SCD6, respectively. The MS2-CP CDS was PCR-amplified from plasmid pJC236 with primers containing an XhoI site and encoding a flexible linker (Gly-Gly-Gly-Gly-Gly-Ser) at the N-terminus, 3xFLAG epitopes, a stop codon and an overlapping sequence (for fusion PCR) at the C-terminus, using forward primer 5’-ATTCATCTCGAGGGTGGTGGTGGTGGTTCTGCTTCTAACTTTACTCAGTTCGTT-3’ and reverse primer 5’-TTACTTGTCATCGTCATCCTTGTAGTCGATGTCATGATCTTTATAATCACCGTCATGGTCTTTGTAGTCGTAGATGCCGGAGTTTGCTGCGAT-3’. Next, the 3’UTR from each gene was amplified from genomic DNA of BY4741, with primers containing an upstream overlapping sequence (for fusion PCR) and a downstream XmaI site, using primers: (i) *NPL3* 3’UTR, forward primer 5’-CATGACATCGACTACAAGGATGACGATGACAAGTAAGCCATTTATATAGTTGAGAAAAAA-3’; reverse primer 5’-ATTTATCCCGGGTACCTATTCTGGCGTGTAATCCTTATCA-3’); (ii) *SBP1* 3’UTR, forward primer 5’-CATGACATCGACTACAAGGATGACGATGACAAGTAATTACTTCTTACCCACATCCCTATT-3’; reverse primer 5’-ATTTATCCCGGGTACCTCTCCGAGGTAGTGAACCATTGAG-3’); and (iii) *SCD6* 3’UTR, forward primer 5’-CATGACATCGACTACAAGGATGACGATGACAAGTAAAATGATGTTTCTATGTAAATTAAGTA-3’; reverse primer 5’-ATTTATCCCGGGTACCCTTTTCTTGTAGTTTGTTGTTCTTAC-3’). Fragments containing linker-MS2CP-FLAG-3’UTR sequences for each gene were generated by fusion PCR using the amplified fragments above, and inserted between the XhoI/XmaI sites of YCplac111-NPL3, YCplac111-SBP1, or YCplac111-SCD6, to generate the constructs pQZ125 (*NPL3-MS2-F*), pQZ126 (*SBP1-MS-F*) and pQZ127 (*SCD6-MS2-F*). Plasmids encoding MS2-FLAG control proteins pQZ128 (*P*_*NPL3*_*-MS2-F*), pQZ129 (*P*_*SBP1*_*-MS2-F*) and pQZ130 (*P*_*SCD6*_*-MS2–F*) (S13 Fig, *right*) were constructed by a strategy similar to that described above but with the *NPL3*, *SBP1* and *SCD6* CDSs absent from the final constructs and an ATG added at the beginning of the MS2 CP-encoding fragment. The specific primers for these constructions were: (i) *P*_*NPL3*_*-MS2-F*: forward primer for *NPL3* promoter with SphI site 5’-ACGAGGGCATGCTATCAATATGCAAATGCTCGGCTC-3’; reverse primer for *NPL3* promoter with ATG 5’-CATTATCCTTATGGTTTTAGCGTAATT-3’; forward primer for MS2-NPL3 3’UTR with ATG 5’-AATTACGCTAAAACCATAAGGATAATGGGTGGTGGTGGTGGTTCTGCTTCT-3’; reverse primer for MS2-NPL3 3’UTR with KasI site 5’-ATTTATGGCGCCTATTCTGGCGTGTAATCCTTATCA-3’); (ii) *P*_*SBP1*_*-MS2-F*: forward primer for *SBP1* promoter with SphI site 5’-ACGAGGGCATGCTCATCGAGCGGAAAATATTGAAAA-3’; reverse primer for *SBP1* promoter with ATG 5’-CATATTTTTCTTCGTTTGAGGGTTTTC-3’; forward primer for MS2-SBP1 3’UTR with ATG 5’-GAAAACCCTCAAACGAAGAAAAATATGGGTGGTGGTGGTGGTTCTGCTTCT-3’; reverse primer for MS2-SBP1 3’UTR with XmaI site 5’-ATTTATCCCGGGTACCTCTCCGAGGTAGTGAACCATTGAG-3’); (iii) *P*_*SCD6*_*-MS2-F*: forward primer for *SCD6* promoter with SphI site 5’-ACGAGGGCATGCTGCTCGTAACAATCTTGGCCTAGC-3’; reverse primer for *SCD6* promoter with ATG 5’-CATTGCCTTGCTGCTGTTTTTCGATGA-3’; forward primer for MS2-SCD6 3’UTR with ATG 5’-TCATCGAAAAACAGCAGCAAGGCAATGGGTGGTGGTGGTGGTTCTGCTTCT-3’; reverse primer for MS2-SCD6 3’UTR with XmaI site 5’-ATTTATCCCGGGTACCCTTTTCTTGTAGTTTGTTGTTCTTAC-3’). The *GFP* reporter plasmid pJC429 was described previously [6]. The *lacZ* reporter plasmid pQZ131 was generated by PCR-amplifying the *lacZ* CDS sequence from *GCN4-lacZ* reporter plasmid p180, adding an SphI site and an ATG to the N-terminus (forward primer 5’-AAACTTGCATGCTTACGGAT-3’), and a PacI site to the C-terminus (reverse primer 5’-ACGAGCTTAATTAATTTTTGACACC-3’). The resulting fragment was inserted between the SphI/PacI sites of pJC429. Plasmid pQZ145 (*DCP2 URA3*) was generated by inserting into pRS316 a 4.3 kb XbaI-XmaI *DCP2* fragment from pRS315-DCP2. Plasmids pQZ139 (ΔLSm-Scd6-MS2-F) and pQZ142 (ΔRGG-Scd6-MS2-F) were generated by deleting the CDS for amino acids Q3-D78 or S287-N318, respectively, of pQZ127 by site-directed mutagenesis (GenScript USA Inc). All plasmids were screened by restriction digestion and DNA sequencing was conducted to verify the presence of the intended inserts.

**Table 1:** Plasmids used in this study.

Plasmid	Relevant Description^a^	Source or Reference
YCplac111	sc *LEU2* cloning vector	[54]
pQZ125	sc *LEU2 NPL3-MS2-FLAG* in YCpLac111	This study
pQZ126	sc *LEU2 SBP1-MS2-FLAG* in YCpLac111	This study
pQZ127	sc *LEU2 SCD6-MS2-FLAG* in YCpLac111	This study
pQZ128	sc *LEU2 MS2-FLAG* under control of *NPL3* in YCpLac111	This study
pQZ129	sc *LEU2 MS2-FLAG* under control of *SBP1* in YCpLac111	This study
pQZ130	sc *LEU2 MS2-FLAG* under control of *SCD6* in YCpLac111	This study
pQZ131	lc *URA3 lacZ* under control of *GAL10* UAS	This study
pQZ139	sc *LEU2 ΔLSm-SCD6-MS2-FLAG* in YCpLac111	This study
pQZ142	sc *LEU2 ΔRGG-SCD6-MS2-FLAG* in YCpLac111	This study
pQZ145	sc *URA3 DCP2* in pRS316	This study
pJC236	sc *LEU2 DHH1-MS2* in YCpLac111	[6]
pJC398	hc *LEU2 MS2* in YEpLac181	[6]
pJC429	lc *URA3 GFP* under control of *GAL10* UAS	[6]
p180	sc *URA3 GCN4-lacZ* in YCp50	[55]
pCGS286	hc *URA3 GAL1-lacZ*	[56]
pRS315-DCP2	lc *LEU2 DCP2*	[57]

^a^sc, single copy number; lc, low copy number; hc, high copy number

### Yeast strains and growth conditions

Yeast strains employed in this work are listed in Table 2. Strain QZY126 was constructed by transforming HFY114 with a DNA fragment containing *dhh1Δ*::*kanMX4* and including ~400 bp of sequences from both upstream and downstream of *DHH1* that was PCR-amplified from genomic DNA of strain 3858 and selecting on YPD medium containing G418. Strain QZY128 was constructed by transforming strain CFY1016 (*dcp2Δ*::*HIS3*) harboring pQZ145 (*DCP2 URA3*) with the *dhh1Δ*::*kanMX4* cassette as above, and evicting pQZ145 by counter-selection growth on 5-FOA medium. FZY843 was constructed by transforming CFY1016 with a fragment containing *scd6Δ*::*KanMX4* that was PCR-amplified from yeast strain 5544. Strains SYY2352 and SYY2353 were constructed by transforming HFY114 with a DNA fragment harboring the *scd6*::*KanMX6* null allele, which contains 400 bp from both upstream and downstream of *SCD6* with the coding region replaced by a 1447-bp *KanMX6* cassette. Gene disruptions were confirmed by PCR analysis of chromosomal DNA using the appropriate primers. Unless otherwise noted, all strains were grown at 30°C in synthetic complete medium without leucine or uracil (SC-L-U) with 2% galactose/2% raffinose replacing dextrose. All cultures were grown for at least two cell divisions and harvested at mid-log phase (OD_600_ = 0.6–0.7).

**Table 2:** Yeast strains used in this study.

Strain	Genotype	Source or reference
BY4741	*MATa his3Δ1 leu2Δ0 met15Δ0 ura3Δ0*	Research Genetics
5544	*MATa his3Δ1 leu2Δ0 met15Δ0 ura3Δ0 scd6Δ*::*kanMX4*	Research Genetics
3858	*MATa his3Δ1 leu2Δ0 met15Δ0 ura3Δ0 dhh1Δ*::*kanMX4*	Research Genetics
387 6925	*MATa his3Δ1 leu2Δ0 met15Δ0 ura3Δ0 ccr4Δ*::*kanMX4* *MATa his3Δ1 leu2Δ0 met15Δ0 ura3Δ0 caf1Δ*::*kanMX4*	Research Genetics Research Genetics
HFY114	*MATa ade2-1 ura3-1 his3-11*,*15 trp1-1 leu2-3*,*112 can1-100*	[57]
CFY1016	*MATa ade2-1 ura3-1 his3-11*,*15 trp1-1 leu2-3*,*112 can1-100 dcp2*::*HIS3*	[57]
QZY126	*MATa ade2-1 ura3-1 his3-11*,*15 trp1-1 leu2-3*,*112 can1-100 dhh1Δ*::*kanMX4*	This study
QZY128 FZY843 SYY2353 SYY2352 SYY2686 SYY2674 SYY2680	*MATa ade2-1 ura3-1 his3-11*,*15 trp1-1 leu2-3*,*112 can1-100 dcp2Δ*::*HIS3 dhh1Δ*::*kanMX4* *MATa ade2-1 ura3-1 his3-11*,*15 trp1-1 leu2-3*,*112 can1-100 dcp2Δ*::*HIS3 scd6Δ*::*KanMX4* *MATa ade2-1 ura3-1 his3-11*,*15 trp1-1 leu2-3*,*112 can1-100 scd6*::*KanMX6* *MATa ade2-1 ura3-1 his3-11*,*15 trp1-1 leu2-3*,*112 can1-100 scd6*::*kanMX6* *MATa ade2-1 ura3-1 his3-11*,*15 trp1-1 leu2-3*,*112 can1-100 dhh1*::*kanMX6* *MATa ade2-1 ura3-1 his3-11*,*15 trp1-1 leu2-3*,*112 can1-100 pat1*::*kanMX6* *MATa ade2-1 ura3-1 his3-11*,*15 trp1-1 leu2-3*,*112 can1-100 lsm1*::*kanMX6*	This study This study This study This study [58] [58] [58]

### Biochemical analyses using yeast cell extracts

For Western blot analysis, whole-cell extracts (WCEs) from at least three biological replicates (independent transformants) were prepared by trichloroacetic acid (TCA) extraction as previously described [43]. Aliquots of WCEs were resolved by 4–20% SDS-PAGE, transferred to PVDF membrane and probed with antibodies against GFP (Covance), Prt1 [44], FLAG epitope (Sigma), or Gcd6 [45]. Immune complexes were detected using the Pierce enhanced chemiluminescence (ECL) system and autoradiography; and signal intensities were quantified by scanning densitometry using NIH ImageJ software. Assays of β-galactosidase activity in WCEs were performed as described previously [46]. At least four biological replicates (and two technical replicates per transformant) were employed for all β-galactosidase assays.

### Polysome analysis and RNA extraction from sucrose gradients

For polysomes profiles, 300 mL of cells were treated with 50 μg/ml cycloheximide for 5 min prior to harvesting. WCEs were prepared in 1x breaking buffer (20 mM Tris-HCl, pH 7.5, 50 mM KCl, 10 mM MgCl_2_, 1 mM DTT, 50 μg/ml cycloheximide, 1 mM PMSF, Complete EDTA-free Protease Inhibitors, 1U/μl SUPERase-In RNase inhibitor) by vortexing with glass beads, followed by two cycles of centrifugation for 10 min at 15,000 rpm at 4°C. 15 OD_260_ units of cleared lysate were loaded on 15%-45% (w/w) sucrose gradients prepared on a Biocomp Gradient Master in 1x breaking buffer and centrifuged at 41,000 rpm for 73 min at 4°C in a Beckman SW41Ti rotor. Gradients were fractionated with a Brandel Fractionation System and ribosomal peaks were visualized at 254 nm with an Isco UV detector. Fractions (0.7 mL) were precipitated overnight at -20°C using 1.5 volumes RNA precipitation mix (95% EtOH, 5% NaOAc), and centrifuged at 15,000 rpm for 30 min to pellet RNA/protein. Pellets were washed in 1 mL cold 80% EtOH, dried in a speed vacuum, and resuspended in 100 μl AE buffer (50 mM NaOAc, 10 mM EDTA, pH 5.2) with 1% SDS. Fractions were extracted by adding 350 μl QIAzol lysis reagent and incubating 5 min at room temperature, adding 70 μL chloroform and incubating 2–3 min at room temperature, and centrifuging at 15,000 rpm for 15 min at 4°C. The aqueous phase (~300 μl) was transferred to a new collection tube, thoroughly mixed with 1 volume of 100% EtOH, and applied to a purification column (RNA Clean and Concentrator™ kit, Zymo Research) to isolate RNA following the vendor’s instructions. RNA from each polysomal fraction was eluted with 25 μl RNase-free water, and 2 μl/reaction were used for first strand cDNA synthesis, as described below.

### Total RNA isolation, RT-qPCR and Northern blot analysis

Yeast cultures (25 mL) were harvested by centrifugation, and the resulting pellets were resuspended in 400 μL AE buffer (50 mM NaOAc, 10 mM EDTA, pH 5.2) with 1% SDS. Cell suspensions were extracted twice with AE buffer-equilibrated phenol, twice with phenol/chloroform/isoamyl alcohol (25:24:1), once with chloroform/isoamyl alcohol (24:1), and then precipitated at -20°C with 2 volumes RNA precipitation mix (95% EtOH, 5% NaOAc). RNA pellets were recovered by centrifugation at 15,000 rpm for 30 min (4°C), washed once with 1 mL cold 80% EtOH, dried in a speed vacuum, and resuspended in 50 μL RNase-free water. Total RNA concentration was calculated at A_260_ using a NanoDrop spectrophotometer. For RT-qPCR, reverse transcription was carried out using SuperScript III First-Strand cDNA Synthesis SuperMix (Invitrogen), with random primers and either 1 μg total cellular RNA from above or 2 μL polysomal RNA isolated from sucrose gradient fractions. qPCR was carried out using Brilliant III Ultra-Fast SYBR Green Master Mix (Agilent) in a Mx3000P System (Stratagene) and the following oligonucleotide pairs: *GFP* (5’-GGCTAGCAAAGGAGAAGAACTC-3’; 5’-CCGTATGTTGCATCACCTTC-3’), *lacZ* (5’-ACCAACGTAACCTATCCCATTAC-3’; 5’-TTCCTGTAGCCAGCTTTCATC-3’), *ACT1* (5’-TGTGTAAAGCCGGTTTTGCC-3’; 5’-GATACCTCTCTTGGATTGAGCTTC-3’). Levels of reporter mRNA relative to actin were calculated using the ΔCt method. For Northern blot analysis, 10 μg of total RNA/lane were denatured with glyoxal/DMSO, separated on 1.4% agarose gels, transferred to nylon membranes, and probed with [^32^P]-end-labeled DNA oligonucleotides complementary to *GFP* or yeast *PYK1* transcripts. Blots were exposed to PhosphorImager screens and scanned using a Storm scanner.

**Measurements of *GFP* mRNA half-lives**. A previously described protocol [47] was employed with the following modifications. Yeast transformants were cultured in 330 mL of SC-U,-L with 2% Galacose and 2% Raffinose to an A_600_ of ~0.8. An aliquot of 30 mL, representing the “0 min” time point, was poured onto ice and collected by centrifugation at 3,000 rpm in a Beckman JS-4.2 rotor for 5 min at 4°C. The cell pellet was resuspended and transferred to a 1.5 mL Eppendorf tube, and spun at 4°C in a microfuge at top speed (15,000 rpm) for 2 min. Residual medium was aspirated and cell pellets were immediately frozen on dry ice. The rest of the culture was collected by centrigugation at room temperature for 8 min at 7,000 rpm in an SLA3000 rotor. The cell pellet was resuspended in an equal volume of SC-U,-L 4% glucose medium pre-warmed to 30°C, and aliquots of 30 mL were harvested exactly as described for the “0 min” sample at 2-, 4-, 6-, 8-, 10-, 15-, -20, -30, -40, and 60 min after glucose containing medium was added. Frozen cells pellets were stored at -80°C until being thawed for isolation of total RNA and qRT-PCR analysis, which was conducted as described above.

### Statistical analysis of tethering data

Changes in reporter protein expression (Δ*GFP* protein or Δβ-galactosidase) or reporter mRNA abundance (Δ*GFP* mRNA or Δ*lacZ* mRNA) were calculated as ratios of mean values of protein or mRNA expression measured in 3 or more biological replicates of transformants expressing Scd6-MS2-F vs. MS2-F alone. The propagated S.E.M.s for the resulting mean ratios were computed as (X/Y)*(√[(SE_x_/x)^2^+(SE_y_/y)^2^]), where X, SE_x_, and x are the mean, standard error of the mean, and highest values for reporter protein input, respectively; and Y, SE_y_, and y are the corresponding values for the mRNA input. Unpaired t-tests were performed to compare the mean changes in reporter protein or mRNA expression between wild type and mutant strains. Changes in TE of reporter mRNA on tethering Scd6-MS2 were determined by calculating the TE of the reporter, as the ratio of reporter protein to reporter mRNA expression, measured in 3 or more pairs of independent transformants expressing Scd6-MS2-F or MS2-F alone, from which the mean ΔTE and S.E.M. was calculated. Unpaired t-tests were performed to compare the resulting mean ΔTE values between wild type and mutant strains.

### Ribosome footprint profiling and RNA-Seq

Ribosome profiling and RNA-Seq analysis were conducted in parallel essentially as described previously [48] on isogenic strains in the W303 background HFY114 (WT), CFY1016 (*dcp2Δ*), SYY2353 (*scd6Δ*), QZY126 (*dhh1Δ*), FZY843 (*dcp2Δscd6Δ*), and QZY128 (*dcp2Δdhh1Δ*), providing two biological replicates of each genotype. Strains growing exponentially in YPD medium at 30°C were harvested by vacuum filtration at room temperature, and quick-frozen in liquid nitrogen. Cells were lysed in a freezer mill with lysis buffer (20 mM Tris [pH 8.0], 140 mM KCl, 1.5 mM MgCl_2_, 1% Triton, 500 μg/mL cycloheximide). For ribosome footprint library preparation, approximately 60 A_260_ units of extract were treated with RNAse I at 15 U per OD_260_ unit (Ambion, #AM2295) for 1h at 25°C on a Thermomixer at 700 rpm, and 80S ribosomes were purified by sedimentation through a sucrose density gradient as described [49]. Ribosome-protected mRNA fragments (footprints) were purified by phenol and chloroform extractions [49]. Following size selection and dephosphorylation, a Universal miRNA cloning linker (Synthesized by Integrated DNA Technologies) was ligated to the 3’ ends of footprints, followed by reverse transcription, circular ligation, rRNA subtraction, PCR amplification of the cDNA library, and DNA sequencing with an Illumina HiSeq system. For RNA-Seq library preparation, total RNA was purified using miRNeasy Mini kit (Qiagen) from aliquots of the same extracts (30 OD_260_ units) used for footprint library preparation, 5 μg total RNA was randomly fragmented at 70°C for 12 min in fragmentation reagent (Ambion #AM8740). Fragment size selection, library generation and sequencing were carried out as above, except Ribo-Zero Gold rRNA Removal Kit (Yeast) was employed to remove rRNAs after linker-ligation in lieu of poly(A) selection. As described [48], linker sequences were trimmed from Illumina reads and the trimmed fasta sequences were aligned to the *S*. *cerevisiae* ribosomal database using Bowtie [50]. The non-rRNA reads (unaligned reads) were then mapped to the *S*. *cerevisiae* genome using TopHat [51]. Wiggle track normalization for viewing RPF or RNA reads in the IGV browser was conducted as follows. Wiggle files were produced from the alignment file, one each for genes on the Watson or Crick strand. The total reads on both strands were summed and a normalization factor *q* was calculated as 1,000,000,000/(total reads on W+C strands). Wiggle files were then regenerated by multiplying all reads by the factor *q*, yielding the number of reads per 1,000 million total reads (rpkm). Statistical analysis of changes in mRNA, RPFs, or TE values between two replicates each of any two strains being compared was conducted using DESeq2 [52], excluding any genes with less than 10 total mRNA reads in the 4 samples combined.

RNA-Seq analysis of strains SYY2352 (*scd6Δ*), SYY2353 (*scd6Δ*), SYY2686 (*dhh1Δ*), SYY2674 (*pat1Δ*), and SYY2680 (*lsm1Δ*) was conducted as described previously [53] after culturing cells in YPD at 30°C; and the results will be described in full in a future publication.

For all notched box-plots, constructed using a web-based tool at http://shiny.chemgrid.org/boxplotr/, the upper and lower boxes contain the 2^nd^ and 3^rd^ quartiles and the band gives the median. If the notches in two plots do not overlap, there is roughly 95% confidence that their medians are different.

### Accession numbers of deposited data

RNA-Seq data employed in the analysis of mRNA changes in *scd6Δ*, *dhh1Δ*, *pat1Δ*, and *lsm1Δ* strains for the group of 83 mRNAs derepressed in *scd6Δ* cells have been deposited in the NCBI Gene Expression Omnibus (GEO; http://www.ncbi.nlm.nih.gov/geo/) under the accessions numbers GEO:GSE107841 and GEO:GSE114428. All other RNA-Seq or Ribo-Seq data generated in this study are deposited separately in GEO:GSE114892.

## Supporting information

S1 Fig Tethered Scd6-MS2-F and Dhh1-MS2 confer similar decreases in the half-life of *GFP* reporter mRNA. **(A)** Transformants from Fig 1B–1C expressing MS2-F, Scd6-MS-F, Dhh1-MS2, and WT strain BY4741 harboring empty vector YCpLac111 (Vector), all harboring *GFP* reporter plasmid pJC429, were analyzed for *GFP* protein expression as in Fig 1B. **(B-C)** Transformants of WT strain HFY114 containing expression plasmids for Scd6-MS2-F (pQZ127) or MS2-F (pQZ130) (B), or Dhh1-MS2 (pJC236) or MS2-F (pQZ130) (C), and *GFP* reporter plasmid pJC429, were cultured in SC-L-U with 2% galactose/2% raffinose and shifted to SC-L-U with 2% glucose to repress reporter mRNA transcription. Total RNA was isolated from cells harvested at the indicated times and subjected to qRT-PCR to measure the amount of *GFP* mRNA remaining at each time point relative to *ACT1* mRNA. The t_1/2_ values were calculated from the slopes of the best-fit lines shown in the plots, k, for the initial rates of decay, using the equation t½ = 0.693/k. Data from two biological replicates are shown for each construct, with the results of an unpaired Student’s t-test on the mean t_1/2_ values measured for Scd6-MS2-F (B) or Dhh1-MS2 (C) vs. MS2-F alone indicated: *, P < 0.05.
(PDF)

S2 Fig Tethering Npl3-MS2-F or Sbp1-MS2-F does not reduce *GFP* reporter protein expression *in vivo*. **(A-B)** WT cells (BY4741) transformed with plasmids expressing MS2_NPL3_-F (pQZ128) or Npl3-MS2-F (pQZ125) and *GFP* reporter plasmid pJC429 were analyzed for *GFP* protein expression as in Fig 1B and 1C. Average results (±S.E.M.s) from at least three biological replicates are represented. **(C-D)** WT cells (BY4741) were co-transformed with plasmids encoding MS2_SBP1_-F (pQZ129) or Sbp1-MS2-F (pQZ126) and pJC429 were analyzed for *GFP* protein expression as in Fig 1B and 1C. Mean values (± S.E.M.s) were determined from at least three biological replicates. (**E**) WCEs of WT cells transformed with plasmids expressing the indicated MS2 fusion proteins were subjected to Western blot analysis using antibodies against FLAG (upper) or Prt1 (lower).
(PDF)

S3 Fig Control experiment showing that tethering MS2-F does not reduce *GFP* reporter protein expression in *dcp2Δ* cells. Transformants of *dcp2Δ* strain CFY1016 harboring expression plasmids for MS2-F (pQZ130) or empty vector YCplac111 and *GFP* reporter pJC429, were analyzed for *GFP* protein expression as in Fig 1B–1D.
(PDF)

S4 Fig Polysome size distribution of *GFP* reporter mRNA is altered on tethering Scd6-MS2-F. **(A-B)** Results from three biological replicate gradients of *dcp2Δ* transformants harboring the *GFP* reporter and expressing MS2-F (A) or Scd6-MS2-F (B), which were averaged to produce the results shown in Fig 3B. WCEs were separated by velocity sedimentation on sucrose density gradients and fractionated with continuous monitoring at A_254_. The abundance of *GFP* mRNA was quantitated by RT-qPCR in total RNA extracted from the gradient fractions and plotted as the percentage of total signal in the gradient.
(PDF)

S5 Fig Additional tethering experiments and controls for the *lacZ* reporter. **(A) Repression of the *lacZ* reporter by tethered Scd6-MS2-F is independent of native Scd6.** Transformants of *scd6Δ* strain 5544 expressing the MS2-F or Scd6-MS2-F fusions from Fig 1 and containing the *lacZ* reporter on pQZ131 were analyzed for β-galactosidase as in Fig 5B. **(B) Expressing Scd6-MS2-F does not affect expression of heterologous *GCN4-lacZ* or *GAL1-lacZ* reporters lacking MS2 CP binding sites.** β-galactosidase activities were determined in WCEs from WT (BY4741) cells harboring plasmids containing a *GCN4-lacZ* reporter (p180) or *GAL1-lacZ* reporter (pCGS286) and expressing either MS2-F (pQZ130) or Scd6-MS2-F (pQZ127), cultured in synthetic complete medium without leucine or uracil (SC-L-U) containing 2% dextrose as carbon source, for p180, or 2% galactose/2% raffinose for pCGS286. **(C) Tethering Npl3-MS2-F or Sbp1-MS2-F does not affect expression of the MS2 CP *lacZ* reporter.** WCEs from WT cells (BY4741) containing either empty vector or the indicated MS2 fusion protein, and pQZ131, were analyzed for β-galactosidase expression as in Fig 5B. **(D) Expression of a heterologous *GAL1-lacZ* reporter lacking MS2CP binding sites is reduced in *dhh1Δ* cells.** β-galactosidase activities were measured in WCEs of isogenic WT (BY4741) or *dhh1Δ* (3858) strains containing a *GAL1-lacZ* reporter on pCGS286, cultured as in Fig 5B. **(E-G) Expression of the *lacZ* reporter is altered in *dhh1Δ* and *dcp2Δ* cells independently of tethered Scd6-MS2-F or MS-F.** Transformants of WT (BY4741) or *dhh1Δ* (3858) strains containing empty vector or the expression plasmids for MS2-F or Scd6-MS2-F described in Fig 1, and pQZ131, were analyzed for expression of β-galactosidase (E) and *lacZ* mRNA (F) as in Fig 5B and 5C. (G) Transformants of *dcp2Δ* strain CFY1016 containing the MS2-F expression plasmid or empty vector and pQZ131 (3858) were analyzed for expression of *lacZ* mRNA. Mean values (± S.E.M.s) were determined from at least three biological replicates. Determination of P-values from significance testing of differences in mean values using an unpaired Student’s t-test, were conducted as described in Supplementary file “Data Analysis and Explanation of Source Files”. P-values are summarized as: **, P <0.01; *, P <0.05.
(PDF)

S6 Fig High reproducibility of RNA-Seq and Ribo-Seq data in biological replicates. **(A-L)** Scatterplots of RNA (A, C, E, G, I, K) or ribosome footprints (B, D, F, H, J, L) read densities (number of reads mapping to each gene’s CDS normalized by the CDS length) for all expressed genes for biological replicates of the following strains: (A-B) HFY114 (WT); (C-D) SYY2353 (*scd6Δ*); (E-F) QZY126 (*dhh1Δ*); (G-H) FZY843 (*dcp2Δscd6Δ*); (I-J) QZY128 (*dcp2Δdhh1Δ*); (K-L) CFY1016 (*dcp2Δ*). Pearson correlation coefficients (r) are indicated in each plot.
(PDF)

S7 Fig Scd6 and Dhh1 cooperate in repressing mRNA abundance and translational efficiencies of a subset of native mRNAs. (**A)** Notched box-plots of log_2_(ΔmRNA) values in the indicated mutants for 346 mRNAs exhibiting ≥2.0-fold increased mRNA abundance in *dhh1Δ(z)* versus WT cells (at FDR<0.01). TE changes were calculated from the data sets described in Fig 7C. **(B-C)** Hierachical clustering analysis conducted as in Fig 8A and 8B RNA-Seq data from *scd6Δ* (SYY2353), *dhh1Δ* (QZY126, *dhh1Δ(z)*), and WT (HFY114) strains. Approximately 50 genes were removed for which no data were available in one of the strains, or where the log_2_(ΔmRNA) value was >4 or <-4 in one of the mutant vs. WT comparisons, after which separate clustering analysis was performed on two sets of mRNAs in which all log_2_(ΔmRNA) values fell between -2 or +2 (panel B, 5213 mRNAs), or in which the log_2_(ΔmRNA) value for one of the mutants was < -2 or > +2 (panel C, 124 mRNAs). The color key for log_2_(ΔmRNA) values is indicated above each analysis. **(D-E)** Notched box-plots of log_2_(ΔTE) values in the indicated mutants for the 83 mRNAs analyzed in Fig 7A exhibiting ≥1.4-fold increased mRNA abundance in *scd6Δ* versus WT cells (D); and for the same 346 mRNAs analyzed in (A), exhibiting ≥2.0-fold increased mRNA abundance in *dhh1Δ(z)* versus WT cells (E). TE changes were calculated from the data sets described in Fig 7C. (In panels A, C, E & F, the double mutants carry an (*) to indicate that their values have been compared to the *dcp2Δ* single mutant rather than to WT.) **(F)** Notched box-plots of log_2_(ΔmRNA) values in the indicated mutants for 53 mRNAs exhibiting ≥1.33-fold increased TE in *scd6Δ* versus WT cells described in Fig 7E. The RNA changes were calculated from the indicated data sets described in (A). For panels A, D, E, & F, the changes in mRNA abundance or TE for the relevant group of mRNAs found in each of the indicated mutants were plotted irrespective of whether the changes exhibit statistical significance in that mutant, to allow a coherent comparison of the behavior of the complete cohort of mRNAs across the entire panel of mutants. Statistical significance is evaluated for differences in the median changes found in the different mutants, with non-overlapping notches indicating with 95% confidence that the median changes found for two mutants differ from one another.
(PDF)

S8 Fig Exemplar genes exhibiting increased TE in both *scd6Δ* and *dhh1Δ* versus WT cells. **(A-D)** Integrated Genomics Viewer (Broad Institute) displays of ribosome-protected fragments (RPFs) and mRNA reads across the indicated genes from two biological replicates each for WT, *scd6Δ* and *dhh1Δ* strains, shown in units of rpkm. Position of the CDS (magenta) is shown at the bottom with the scale in bp; scales of rpkm for each track are on the left, and calculated ΔRPF, ΔmRNA and ΔTE values between each mutant and WT are on the right.
(PDF)

S9 Fig Scd6 and Dhh1 repress mRNA abundance of genes involved in metabolism of energy reserves and other carbohydrates. GO term analysis conducted using web tool Funspec at http://funspec.med.utoronto.ca/ applying Bonferroni correction. The functional categories showing enrichment derive from the MIPS database. k/n/f: number of genes in MIPS category/number of genes in up-regulated list/total number of genes in MIPS category. **(A)** 346 mRNAs exhibiting ≥2.0-fold increased mRNA abundance in *dhh1Δ(z)* versus WT cells at FDR<0.01; analyzed in S7A Fig. **(B)** 83 mRNAs exhibiting ≥1.4-fold increased mRNA abundance in *scd6Δ* versus WT cells at FDR<0.01; analyzed in Fig 7A.
(PDF)

S10 Fig Dhh1 occupancy tends to be elevated for mRNAs showing increased abundance, ribosome occupancy or TE in *dhh1Δ* vs. WT cells. **(A-C)** Boxplot analysis of changes in ribosome occupancy (A), TE (B) or mRNA abundance (C) versus Dhh1 RIP-seq enrichment values from Miller et al (2018). The latter were equally divided into five pentiles of 739 genes from lowest to highest enrichment values and plotted against the log_2_(Δribo) values (A), log_2_(ΔTE) values (B), or log_2_(ΔmRNA) values determined by ribosome profiling analysis of *dhh1Δ* strain (QZY126, *dhh1Δ(z)*) and WT strain HFY114. The Pearson correlation coefficients for the relationship between log_2_(Δribo) values (panel A), log_2_(ΔTE) values (panel B), or log_2_(ΔmRNA) values (panel C) and Dhh1 enrichment for all mRNAs are 0.31 (P = 3 X 10^−81^), 0.18 (P = 1 X 10^−29^), and 0.27 (P = 6 X 10^−62^), respectively.
(PDF)

S11 Fig Low codon optimality is associated with increased mRNA abundance in *dhh1Δ* vs. WT cells. **(A-C).** Yeast mRNAs were binned by sTAI values, a measure of overall codon optimality [10] and the log_2_(ΔmRNA) values measured by RNA-Seq in three independent analyses of *dhh1Δ* vs. WT strains (described in S7A Fig) were displayed in a box-plot for each bin. In all cases, the bin containing the lowest sTAI optimality scores (median of ~0.25) shows greater increases in mRNA expression in the *dhh1Δ* mutant vs WT compared to the bin containing the highest sTAI optimality scores (median of ~0.61), as observed previously [10]. However, the magnitude of this difference is relatively less for the *dhh1Δ* datasets from Jungfleisch et al. [37] (B) and the current study (C) compared to that of Radhakrishnan et al. [10] (A).
(PDF)

S12 Fig Low codon optimality appears to play a minor role in conferring repression of mRNA abundance by Scd6. **(A)** Yeast mRNAs were binned by sTAI values as in S11 Fig and the log_2_(ΔmRNA) values measured by RNA-Seq analysis of *scd6Δ* (SYY2353) vs. WT (HFY114) cells. The bin containing the lowest sTAI optimality scores (median of ~0.25) shows greater increases in mRNA expression in the *scd6Δ* mutant vs WT compared to the bin containing the highest sTAI optimality scores (median of ~0.61), as observed previously for a *dhh1Δ* mutant [10]. **(B)** The sTAI values are significantly higher for the group of 83 mRNAs found by RNA-Seq to exhibit elevated abundance in *scd6Δ* strains (SYY2352 and SYY2353) vs. WT strain (HFY114), compared to all mRNAs.
(PDF)

S13 Fig Schematics of expression constructs for MS2 fusion and control proteins for in vivo tethering assays. Scd6-MS2-F, Npl3-MS2-F, and Spb1-MS-F fusion proteins were expressed under the control of their native promoters and 5’UTR and 3’UTR sequences, with the complete CDS of each protein fused in-frame at the C-terminus to a 5-amino acid linker, followed by the coding sequences for MS2 CP and three FLAG epitopes. The MS2-FLAG control constructs are identical except that they lack the respective Scd6/Npl3/Spb1 CDSs. The fusion protein expression constructs are contained on the following plasmids: *Ρ*_*SCD6*_*-SCD6-MS2-F* (pQZ127), *Ρ*_*NPL3*_*-NPL3-MS2-F* (pQZ125), and *Ρ*_*SBP1*_*-SBP1-MS2-F* (pQZ126). The corresponding MS2-F control constructs are as follows: *Ρ*_*SCD6*_*-MS2-F* (pQZ130), *Ρ*_*NPL3*_*-MS2-F* (pQZ128), and *Ρ*_*SBP1*_*-MS2-F* (pQZ129).
(PDF)

S1 Text Analysis and Explanation of Supporting Data Files. Description of procedures employed to analyze the source data found in supporting data files S1-S10 to (i) calculate mean values from replicate determinations of reporter protein or reporter mRNA expression, reporter TE, or changes in these parameters between mutant and WT cells, and conduct statistical analysis of observed differences in the corresponding means, for results in Figs 1, 2, 4, 5, 6, S2, S3 and S5; (ii) conduct statistical analysis of differences in observed mean values of reporter mRNA levels in different polysome gradient fractions for results in Fig 3; (iii) calculate reporter mRNA mean half-lives and conduct statistical analysis of differences in the corresponding mean values for results in S1 Fig.
(PDF)

S1 Data Source data and statistical analysis for Fig 1C, 1D and 1F. For Fig 1B and 1C, *GFP* protein expression was analyzed by quantification of the *GFP* protein and loading control (LC, Prt1) signals on immunoblots by densitometry and the GFP/LC ratio was calculated for each biological replicate (BR) of Scd6-MS2-F or MS2-F expressing transformants. The mean GFP/LC ratio, and both the standard deviation and S.E.M., were calculated from between 3 and 6 biological replicates of Scd6-MS2-F or MS2-F expressing transformants. An unpaired Student’s t-test was performed comparing the mean GFP/LC ratios between the Scd6-MS2-F and MS2-F transformants and the magnitude of the P-values are summarized as: **, P <0.01; *, P <0.05 in Fig 1C. For Fig 1D, *GFP* mRNA expression was analyzed by determining the mean 2^-Ct^ values from 3 technical replicates (TR) for *GFP* mRNA, and also for *ACT1* mRNA for the same RNA samples, and the ratio of *GFP*/*ACT1* 2^-Ct^ values was calculated from the ratio of the resulting mean 2^-Ct^ values. The *GFP*/*ACT1* ratios thus determined from five or more biological replicates were averaged for Scd6-MS2-F or MS2-F expressing transformants, and the mean values and both the standard deviation and S.E.M. were calculated. An unpaired Student’s t-test was performed comparing the mean *GFP*/*ACT1* 2^-Ct^ values between the Scd6-MS2-F and MS2-F transformants and the magnitudes of the P-values are summarized as: **, P <0.01; *, P <0.05 in Fig 1D. For Fig 1F, changes in *GFP* protein or mRNA expression, or in TE values, on tethering Scd6-MS2 vs. MS2 alone were calculated as follows. The change in *GFP* protein (Δ*GFP* Protein) was calculated as the ratio of the mean values from panel C for Scd6-MS2-F vs. MS2-F. The propagated S.E.M. for the resulting ratio of means was calculated using the formula: (X/Y)*(√[(SE_x/x)^2^+(SE_y/y)^2^]), where X, SE_x, and x are the mean, standard error of the mean, and highest values for Scd6-MS2-F, respectively; and Y, SE_y, and y are the corresponding values for MS2-F. The change in *GFP* mRNA (Δ*GFP* mRNA) and S.E.M. were calculated in the same way, and the resulting mean and S.E.M.s for Δ*GFP* protein and Δ*GFP* mRNA were entered in Fig 1F. Changes in TE of *GFP* mRNA on tethering Scd6-MS2 vs. MS2 alone were calculated as follows, noting that aliquots of cell cultures used for *GFP* protein and *GFP* mRNA were taken from the same biological replicate culture. For each pair of biological replicates expressing Scd6-MS2-F or MS2-F, the change in *GFP* protein and change in *GFP* mRNA were calculated as the Scd6-MS2-F/MS2-F ratios from the corresponding *GFP* protein and *GFP* mRNA values (normalized to Prt1 or *ACT1* mRNA as described above), for that pair of transformants. The ratio of the resulting changes in *GFP* Protein to changes in *GFP* mRNA was calculated to determine the change in TE (ΔTE) on tethering Scd6-MS2-F vs. MS2-F for that pair of transformants. The mean ΔTE and S.E.M. was calculated by averaging the ΔTE values for six different pairs of biological replicates of Scd6-MS2-F and MS2-F transformants, and entered in Fig 1F.
(XLSX)

S2 Data Source data and statistical analysis for Fig 2B–2E. For Fig 2B and 2C, *GFP* protein (B) and *GFP* mRNA (C) expression data in each WT and mutant strain were analyzed exactly as described for Fig 1C and 1D, respectively, in S1 Data for the WT strain; and an unpaired Student’s t-test was performed comparing the mean GFP/LC ratios (B) mean *GFP*/*ACT1* 2^-Ct^ values (C) between the Scd6-MS2-F and MS2-F transformants in each strain. For Fig 2D, changes in *GFP* protein or *GFP* mRNA expression on tethering Scd6-MS2 vs. MS2 alone in each strain were calculated as described for Fig 1F in supporting file S1 Data for the WT strain. An unpaired Student’s t-test was performed comparing the mean Δ*GFP* protein or Δ*GFP* mRNA values between different strains using the propagated S.E.M. values (calculated using the formula employed for Fig 1F) and the number (N) of BRs examined in each strain in comparing Scd6-MS2-F to MS2-F transformants. For Fig 2E, changes in TE of *GFP* mRNA on tethering Scd6-MS2 vs. MS2 alone in each strain were calculated as described for Fig 1F in supporting file S1 Data for the WT strain. An unpaired Student’s t-test was performed comparing the mean ΔTE values between pairs of Scd6-MS2-F and MS2-F transformants determined in each mutant vs. WT. Magnitudes of P-values from t-tests are summarized in Fig 2B–2E as: **, P <0.01; *, P <0.05; n.s., not significant.
(XLSX)

S3 Data Source data and statistical analysis for Fig 3B and 3C. For each of three biological replicate transformants (BR1-BR3) expressing Scd6-MS2-F or MS2-F alone, 2^-Ct^ values were determined in triplicate (technical replicates TR1-TR3) for *GFP* or *ACT1* mRNA from the RNA isolated from each gradient fraction and averaged. The average 2^-Ct^ value for each fraction was plotted as a proportion of the sum of the average 2^-Ct^ values for all gradient fractions in Fig 3B for *GFP* mRNA and in Fig 3C for *ACT1* mRNA. An unpaired Student’s t-test was performed comparing the mean proportions of *GFP* mRNA in each fraction for Scd6-MS2-F versus MS2-F transformants, and the magnitudes of P-values are summarized in Fig 3B and 3C as: **, P <0.01; *, P <0.05.
(XLSX)

S4 Data Source data and statistical analysis for Fig 4A–4D. (**A-B, D**) *GFP* protein (A) or *GFP* mRNA (B,D) expression data in WT and *ccr4Δ* (A-B) or *caf1Δ* (D) strains were analyzed exactly as described for Fig 1C and 1D in supporting file S1 Data, respectively, for the WT strain. (**C-D**) Changes in *GFP* protein (C) or *GFP* mRNA expression (C-D) on tethering Scd6-MS2 vs. MS2 alone in each mutant or WT strain were analyzed as described for Fig 2D in supporting file S2 Data. Changes in TE of *GFP* mRNA on tethering Scd6-MS2 vs. MS2 alone in each mutant or WT strain (C) were analyzed as described for Fig 2E in supporting file S2 Data.
(XLSX)

S5 Data Source data and statistical analysis for Fig 5B–5E. For Fig 5B, units of β-galactosidase activity measured for three or more biological replicates (BR) of Scd6-MS2-F- or MS2-F-expressing transformants of each WT or mutant strain were averaged; and an unpaired Student’s t-test was performed comparing the mean activities between the Scd6-MS2-F and MS2-F transformants in that strain. For Fig 5C, *lacZ* mRNA expression data in each strain were analyzed exactly as described for *GFP* mRNA for Fig 1D in supporting file S1 Data, for the WT strain. For Fig 5D, changes in β-galactosidase activity or *lacZ* mRNA expression on tethering Scd6-MS2 vs. MS2 alone in each strain were analyzed as described for Fig 2D in supporting file S2 Data. For Fig 5E, changes in TE of *lacZ* mRNA on tethering Scd6-MS2 vs. MS2 alone in each strain were analyzed as described for Fig 2E in Source Data File 2.
(XLSX)

S6 Data Source data and statistical analysis for Fig 6D–6G. *GFP* protein (Fig 6D & 6F), *GFP* mRNA (Fig 6E), and β-galactosidase (Fig 6G) expression data in WT transformants expressing WT or mutant derivatives of Scd6-MS2-F, or MS2-F alone, were analyzed as described for Fig 1C and Fig 1D in supporting file S1 Data, and for Fig 5B in file S5 Data, respectively.
(XLSX)

S7 Data Source data and statistical analysis for S1B and S1C Fig. Analysis of RT-qPCR data to calculate the percent normalized *GFP* mRNA found at time = 0 min remaining at each time point following a shift from galactose to glucose as carbon source; determination of the slopes (k) of semi-log plots of the resulting values versus time; and calculation of t_1/2_ values as 0.693/k were analyzed for WT strains expressing the *GFP* reporter and Scd6-MS2-F or MS2-F (B); and Dhh1-MS2 or MS2-F (C).
(XLSX)

S8 Data Source data and statistical analysis for S2B & S2D Fig. *GFP* protein expression data in WT transformants expressing Npl3-MS2-F or MS2-F alone, both expressed from the *NPL3* promoter (S2B Fig); or Sbp1-MS2-F or MS2-F alone, both expressed from the *SBP1* promoter (S2D Fig), were analyzed as described for Fig 1C in supporting file S1 Data.
(XLSX)

S9 Data Source data and statistical analysis for S3 Fig. *GFP* protein expression data in WT or *dcp2Δ* transformants expressing MS2-F, or containing empty vector, were analyzed as described for Fig 1C in supporting file S1 Data.
(XLSX)

S10 Data Source data and statistical analysis for S5A–S5G Fig. For S5A–S5E Fig, β-galactosidase expression data were analyzed as described for Fig 5B in supporting file S5 Data. For S5F and S5G Fig, *lacZ* mRNA expression data were analyzed as described for Fig 5C in file S5 Data.
(XLSX)
