# Correction: Conserved mRNA-granule component Scd6 targets Dhh1 to repress translation initiation and activates Dcp2-mediated mRNA decay *in vivo*

**DOI:** 10.1371/journal.pgen.1008299

**Published:** 2019-07-23

**Authors:** Quira Zeidan, Feng He, Fan Zhang, Hongen Zhang, Allan Jacobson, Alan G. Hinnebusch

The authors wish to state that the original version of Fig 6A and its legend contain several errors regarding the Scd6 amino acids present in the three Scd6-MS2-F constructs. Specifically, Scd6-MS2-F (top construct) contains all Scd6 amino acids from residues 1 to 349, ΔLSm-Scd6-MS2-F (middle construct) lacks amino acids 3–78 near its N-terminus, and ΔRGG-Scd6-MS2-F (lower panel) lacks amino acids 287–318 near its C-terminus. The correct version of Fig 6 and its legend are provided below.

**Fig 6:**
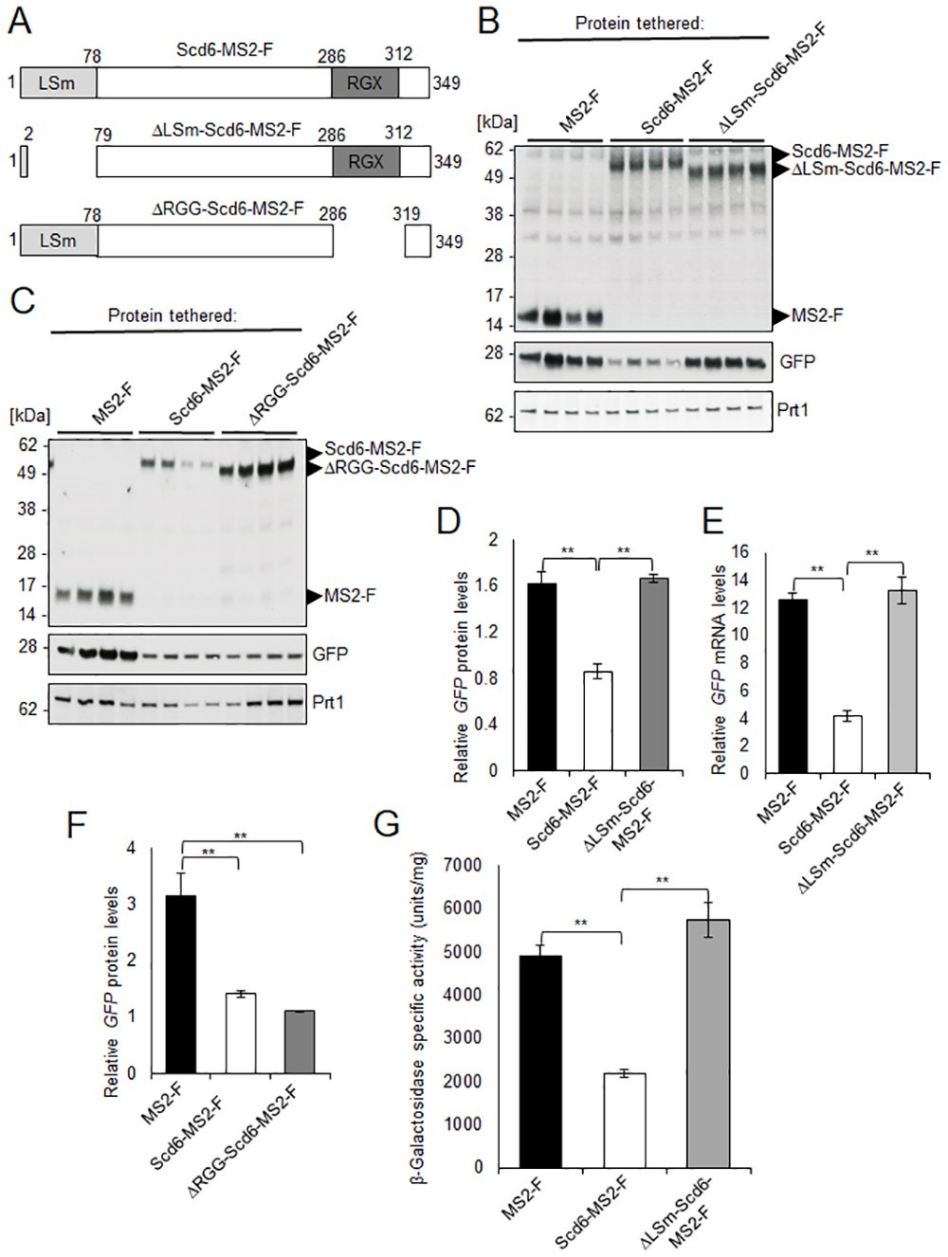
Evidence that the conserved N-terminal LSm domain is essential for translational repression and stimulation of mRNA decay by tethered Scd6-MS2-F *in vivo*. (A) Schematic diagrams (not to scale) representing full-length Scd6 (Scd6-MS2-F; upper panel) or variants lacking amino acids 3–78 at its N-terminus (ΔLSm-Scd6-MS2-F; middle panel) or amino acids 287–318 at its C-terminus (ΔRGG-Scd6-MS2-F; lower panel), present in the corresponding fusions to MS2-F. MS2 and FLAG tags are not depicted. (B-F) Transformants of WT strain BY4741 containing the *GFP* reporter plasmid pJC429 and expression plasmids for MS2-F (pQZ130) and Scd6-MS2-F (pQZ127), and either ΔLSm-Scd6-MS2-F (pQZ139) (B, D-E) or ΔRGG-Scd6-MS2-F (pQZ142) (C & F) were analyzed for *GFP* protein (B-D & F) and mRNA (E) expression as in Fig. 1B-1D. (G) Transformants of WT strain BY4741 harboring the *lacZ* reporter plasmid pQZ131 and the expression plasmids for MS2-F, Scd6-MS2-F, or ΔLSm-Scd6-MS2-F used in (B) were analyzed for β-galactosidase expression as in Fig. 5B. Mean values (S.E.M.s) were determined for at least four biological replicates. Determination of P-values from significance testing of differences in mean values in (D-G) using an unpaired Student’s t-test, were conducted as described in the supporting file S1 Text. P-values are summarized as: **, P <0.01; *, P <0.05.

In addition, fully annotated DNA sequences of the complete DNA fragments encoding the fusion proteins present in plasmids pQZ127 (Scd6-MS2-F), pQZ139 (ΔLSm-Scd6-MS2-F), and pQZ142 (ΔRGG-Scd6-MS2-F) are provided in S1 Appendix.

These corrections do not influence the corresponding results of experiments employing these plasmids and the expressed chimeric proteins. We wish to apologize for any difficulties these errors may have caused.

## Supporting information

S1 Appendix DNA sequences of SCD6 constructs. Fully annotated DNA sequences of the complete DNA fragments encoding the fusion proteins present in plasmids described in the correct version of Fig 6A, and the multiple sequence alignment delimiting the Scd6 LSm and RGG domains.
(PDF)
