# Supplementary figures and images for: Conserved mRNA-granule component Scd6 targets Dhh1 to repress translation initiation and activates Dcp2-mediated mRNA decay in vivo
Source: PLoS Genet. 2018 Dec 7;14(12):e1007806. doi: 10.1371/journal.pgen.1007806 (PMC6307823; doi:10.1371/journal.pgen.1007806)

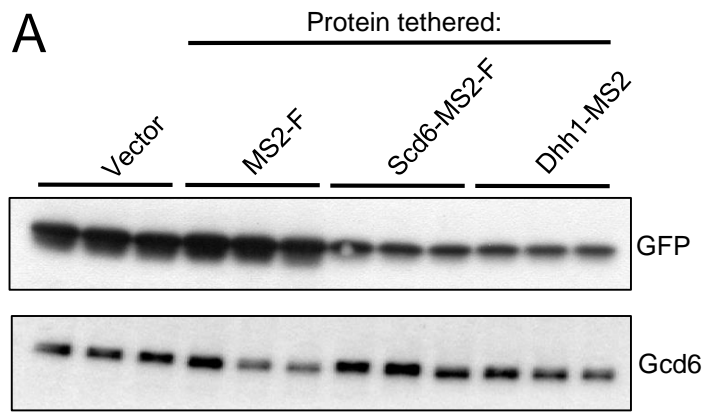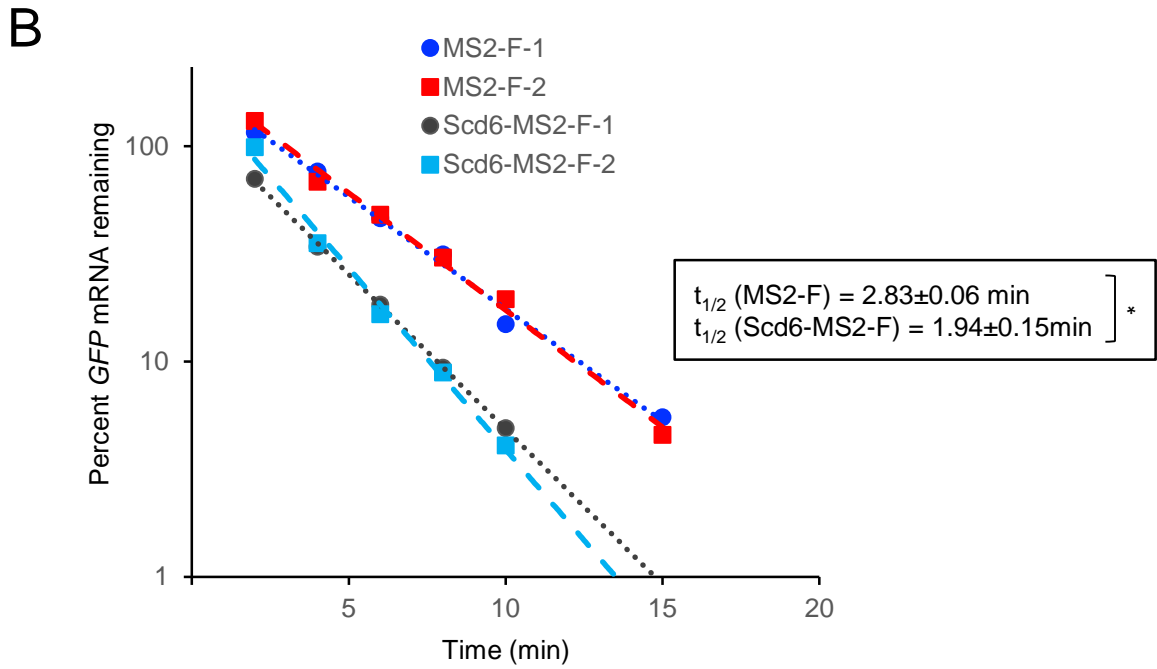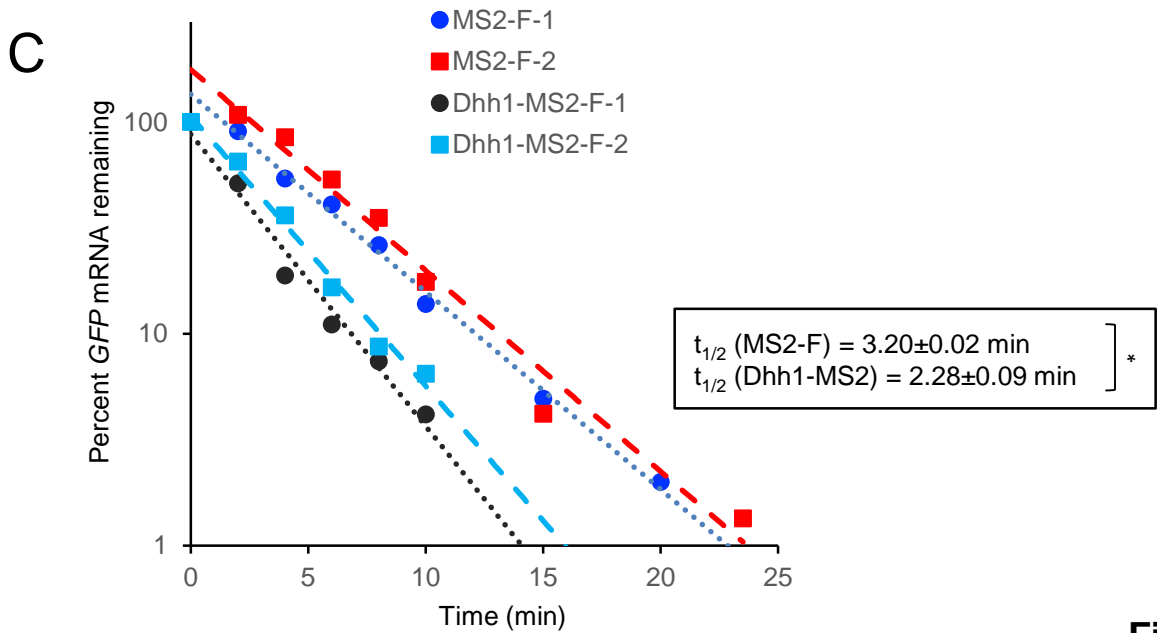

**Figure S1**

Supplement: S1 Fig — (A) Transformants from Fig 1B–1C expressing MS2-F, Scd6-MS-F, Dhh1-MS2, and WT strain BY4741 harboring empty vector YCpLac111 (Vector), all harboring GFP reporter plasmid pJC429, were analyzed for GFP protein expression as in Fig 1B. (B-C) Transformants of WT strain HFY114 containing expression plasmids for Scd6-MS2-F (pQZ127) or MS2-F (pQZ130) (B), or Dhh1-MS2 (pJC236) or MS2-F (pQZ130) (C), and GFP reporter plasmid pJC429, were cultured in SC-L-U with 2% galactose/2% raffinose and shifted to SC-L-U with 2% glucose to repress reporter mRNA transcription. Total RNA was isolated from cells harvested at the indicated times and subjected to qRT-PCR to measure the amount of GFP mRNA remaining at each time point relative to ACT1 mRNA. The t1/2 values were calculated from the slopes of the best-fit lines shown in the plots, k, for the initial rates of decay, using the equation t½ = 0.693/k. Data from two biological replicates are shown for each construct, with the results of an unpaired Student’s t-test on the mean t1/2 values measured for Scd6-MS2-F (B) or Dhh1-MS2 (C) vs. MS2-F alone indicated: *, P < 0.05. (PDF) [file pgen.1007806.s001.pdf]

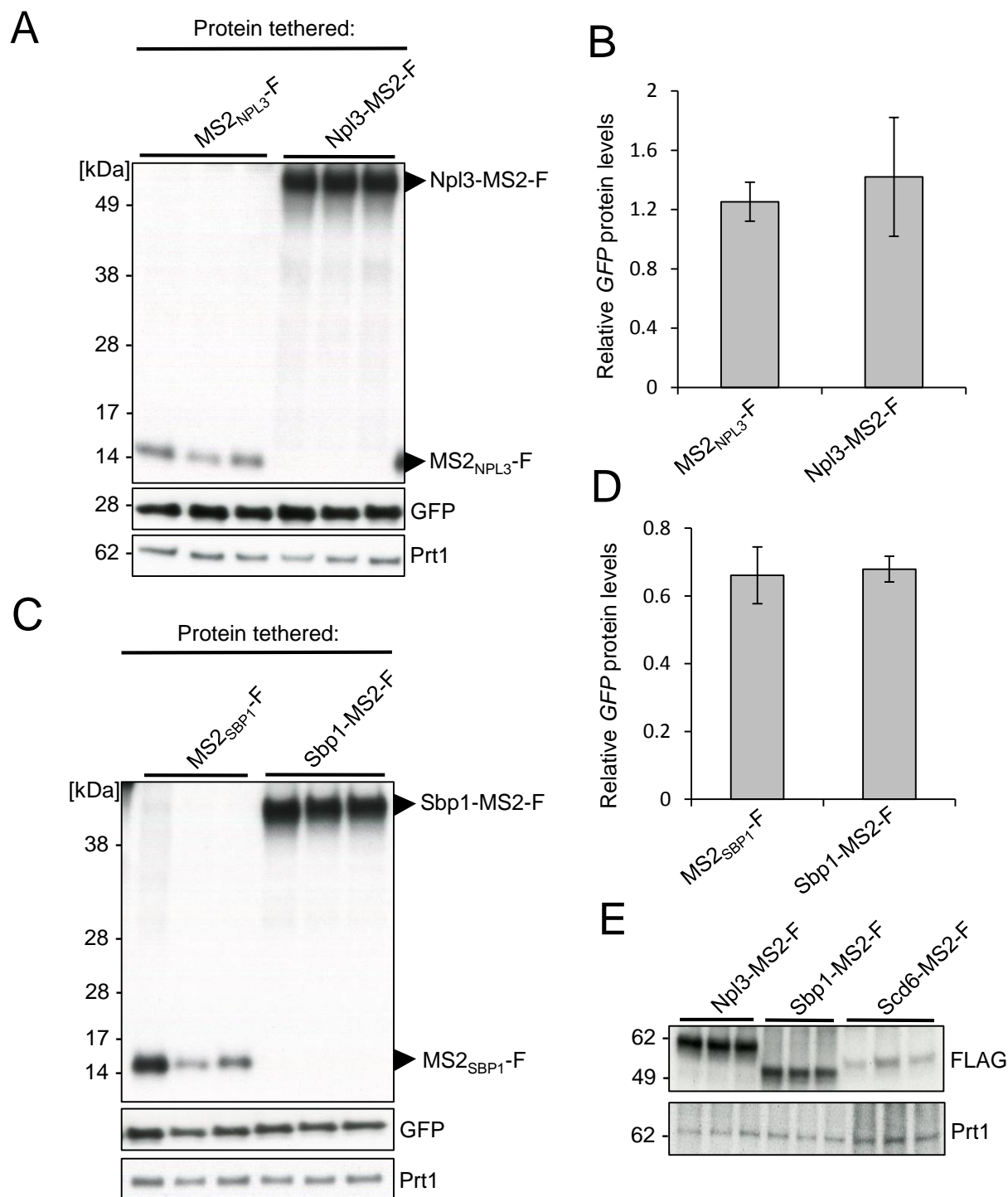

**Figure S2**

Supplement: S2 Fig — (A-B) WT cells (BY4741) transformed with plasmids expressing MS2NPL3-F (pQZ128) or Npl3-MS2-F (pQZ125) and GFP reporter plasmid pJC429 were analyzed for GFP protein expression as in Fig 1B and 1C. Average results (±S.E.M.s) from at least three biological replicates are represented. (C-D) WT cells (BY4741) were co-transformed with plasmids encoding MS2SBP1-F (pQZ129) or Sbp1-MS2-F (pQZ126) and pJC429 were analyzed for GFP protein expression as in Fig 1B and 1C. Mean values (± S.E.M.s) were determined from at least three biological replicates. (E) WCEs of WT cells transformed with plasmids expressing the indicated MS2 fusion proteins were subjected to Western blot analysis using antibodies against FLAG (upper) or Prt1 (lower). (PDF) [file pgen.1007806.s002.pdf]

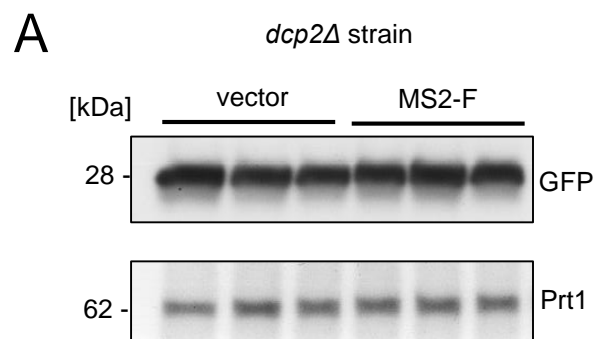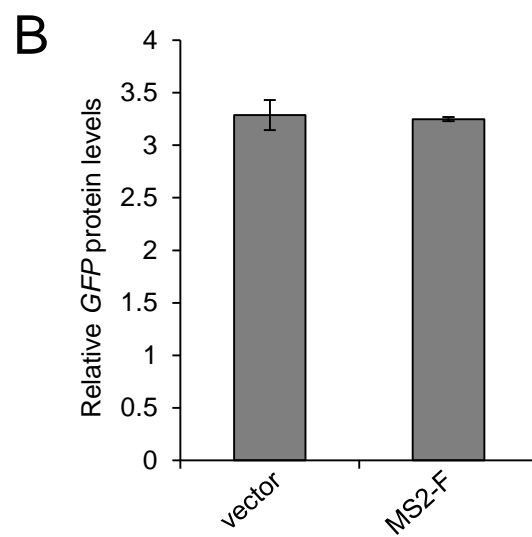

**Figure S3**

Supplement: S3 Fig — Transformants of dcp2Δ strain CFY1016 harboring expression plasmids for MS2-F (pQZ130) or empty vector YCplac111 and GFP reporter pJC429, were analyzed for GFP protein expression as in Fig 1B–1D. (PDF) [file pgen.1007806.s003.pdf]

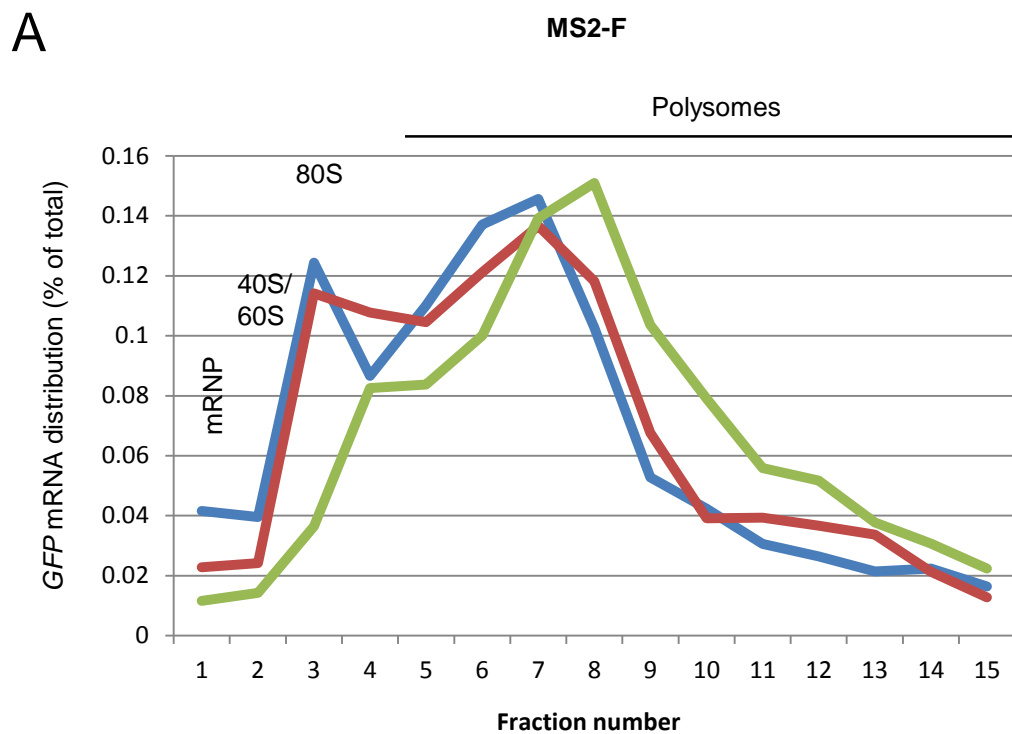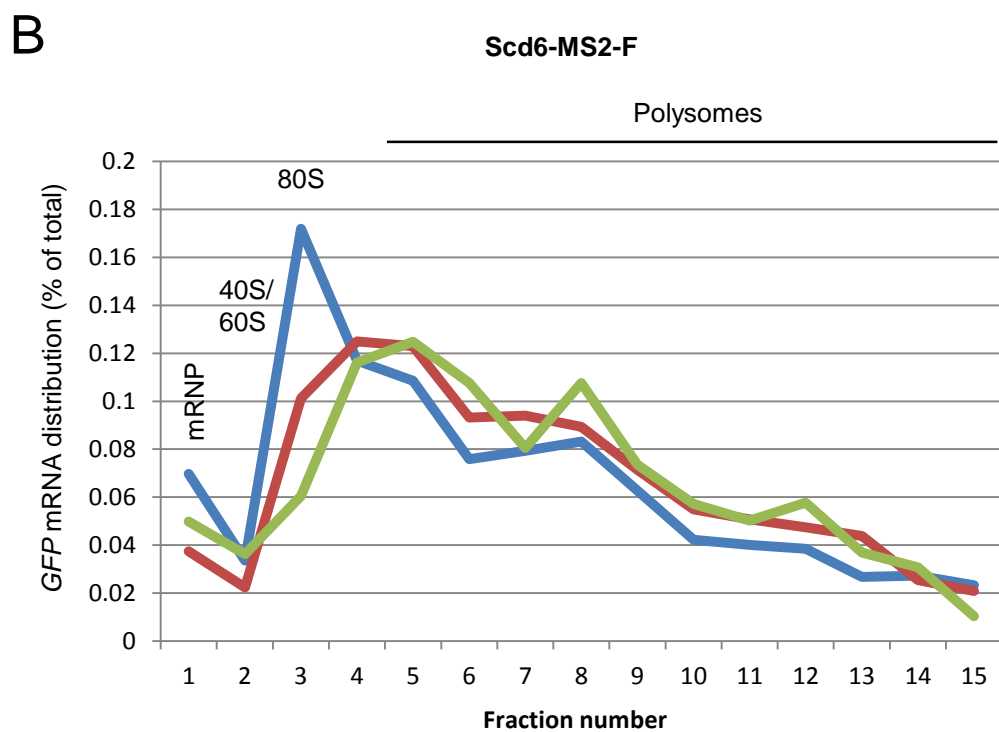

**Figure S4**

Supplement: S4 Fig — (A-B) Results from three biological replicate gradients of dcp2Δ transformants harboring the GFP reporter and expressing MS2-F (A) or Scd6-MS2-F (B), which were averaged to produce the results shown in Fig 3B. WCEs were separated by velocity sedimentation on sucrose density gradients and fractionated with continuous monitoring at A254. The abundance of GFP mRNA was quantitated by RT-qPCR in total RNA extracted from the gradient fractions and plotted as the percentage of total signal in the gradient. (PDF) [file pgen.1007806.s004.pdf]

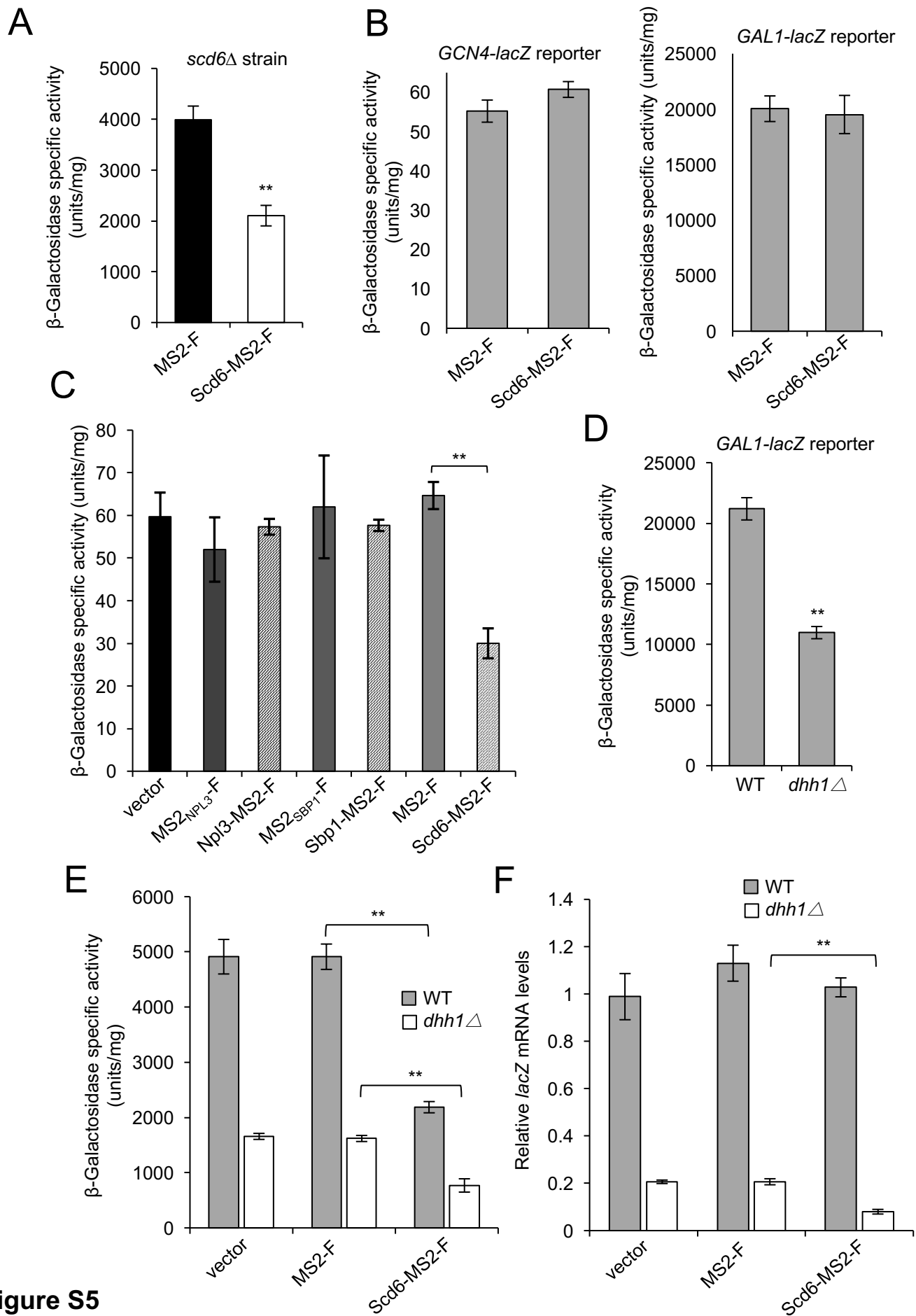

**Figure S5**

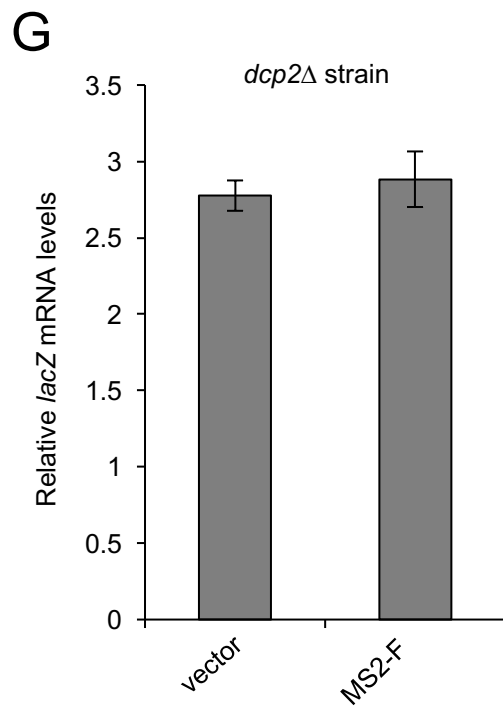

**Figure S5 continued**

Supplement: S5 Fig — (A) Repression of the lacZ reporter by tethered Scd6-MS2-F is independent of native Scd6. Transformants of scd6Δ strain 5544 expressing the MS2-F or Scd6-MS2-F fusions from Fig 1 and containing the lacZ reporter on pQZ131 were analyzed for β-galactosidase as in Fig 5B. (B) Expressing Scd6-MS2-F does not affect expression of heterologous GCN4-lacZ or GAL1-lacZ reporters lacking MS2 CP binding sites. β-galactosidase activities were determined in WCEs from WT (BY4741) cells harboring plasmids containing a GCN4-lacZ reporter (p180) or GAL1-lacZ reporter (pCGS286) and expressing either MS2-F (pQZ130) or Scd6-MS2-F (pQZ127), cultured in synthetic complete medium without leucine or uracil (SC-L-U) containing 2% dextrose as carbon source, for p180, or 2% galactose/2% raffinose for pCGS286. (C) Tethering Npl3-MS2-F or Sbp1-MS2-F does not affect expression of the MS2 CP lacZ reporter. WCEs from WT cells (BY4741) containing either empty vector or the indicated MS2 fusion protein, and pQZ131, were analyzed for β-galactosidase expression as in Fig 5B. (D) Expression of a heterologous GAL1-lacZ reporter lacking MS2CP binding sites is reduced in dhh1Δ cells. β-galactosidase activities were measured in WCEs of isogenic WT (BY4741) or dhh1Δ (3858) strains containing a GAL1-lacZ reporter on pCGS286, cultured as in Fig 5B. (E-G) Expression of the lacZ reporter is altered in dhh1Δ and dcp2Δ cells independently of tethered Scd6-MS2-F or MS-F. Transformants of WT (BY4741) or dhh1Δ (3858) strains containing empty vector or the expression plasmids for MS2-F or Scd6-MS2-F described in Fig 1, and pQZ131, were analyzed for expression of β-galactosidase (E) and lacZ mRNA (F) as in Fig 5B and 5C. (G) Transformants of dcp2Δ strain CFY1016 containing the MS2-F expression plasmid or empty vector and pQZ131 (3858) were analyzed for expression of lacZ mRNA. Mean values (± S.E.M.s) were determined from at least three biological replicates. Determination of P-values from significance testing of differ [file pgen.1007806.s005.pdf]

**A**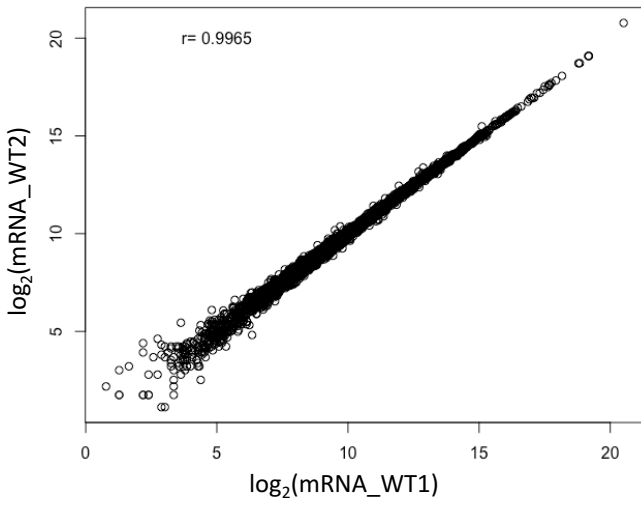**B**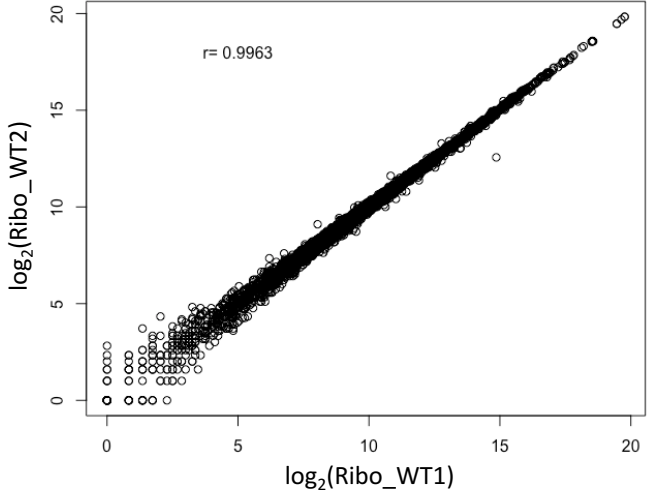**C**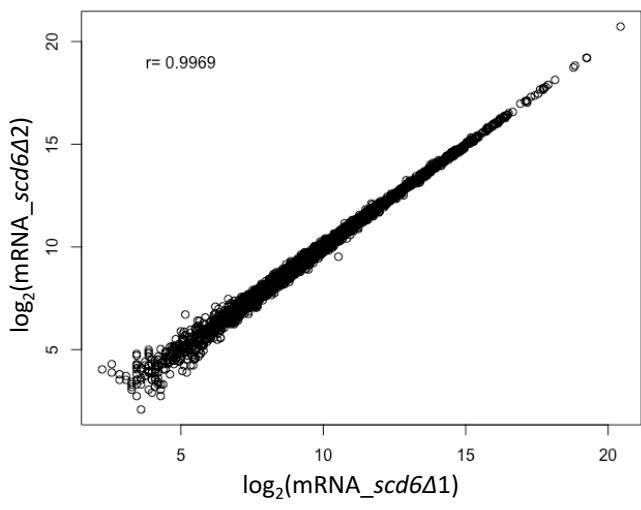**D**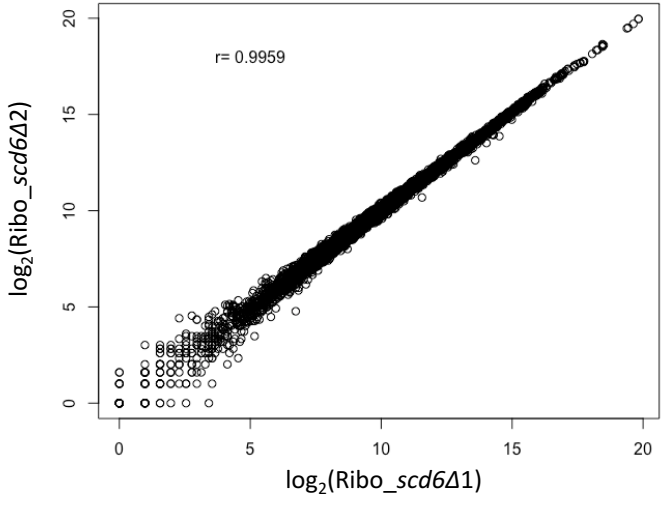**E**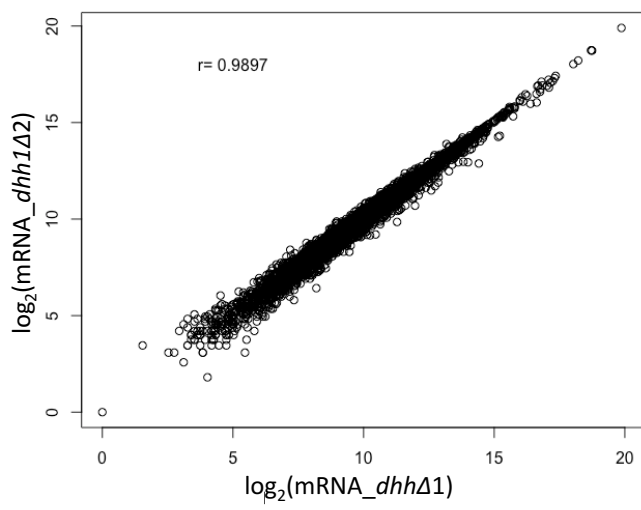**F**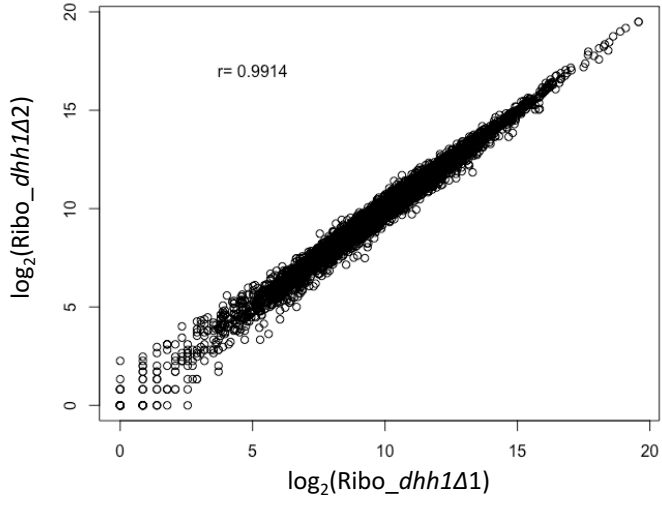**Figure S6**

G

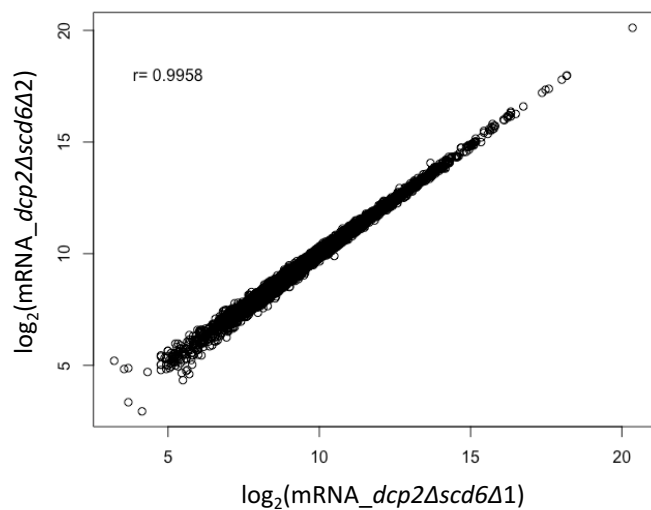

H

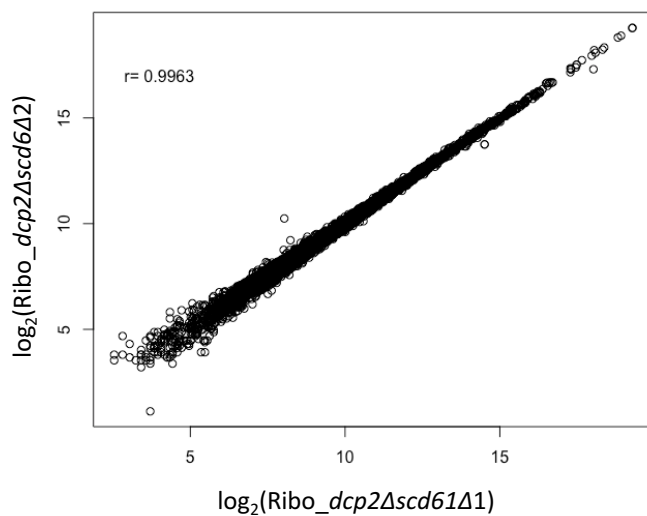

I

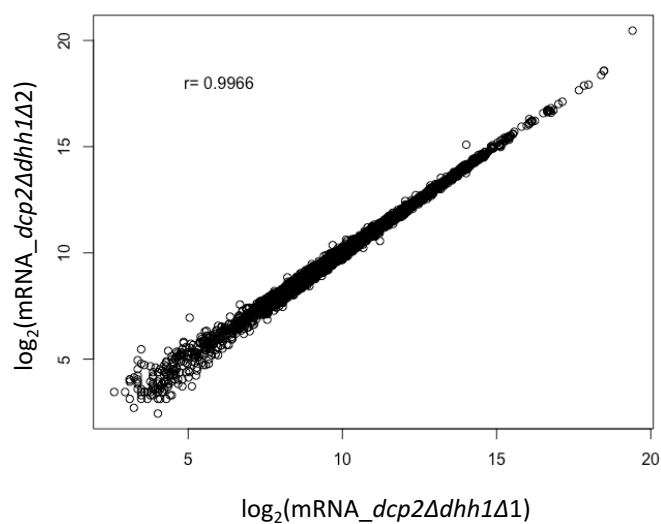

J

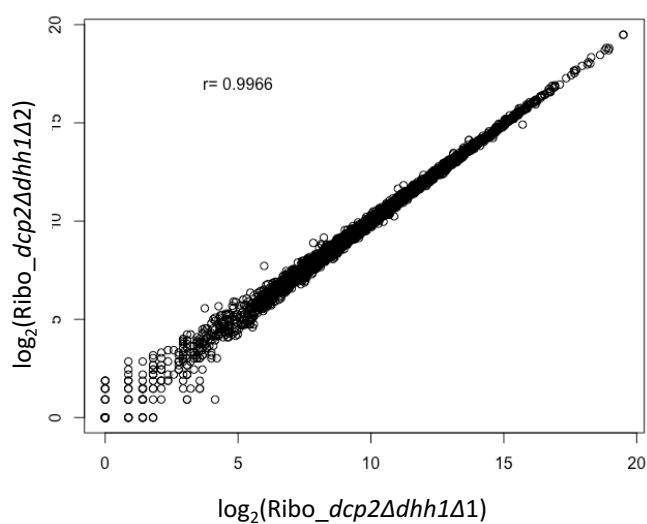

K

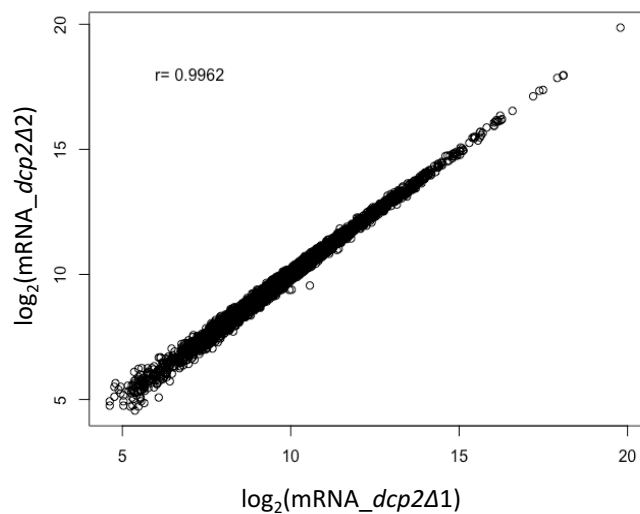

L

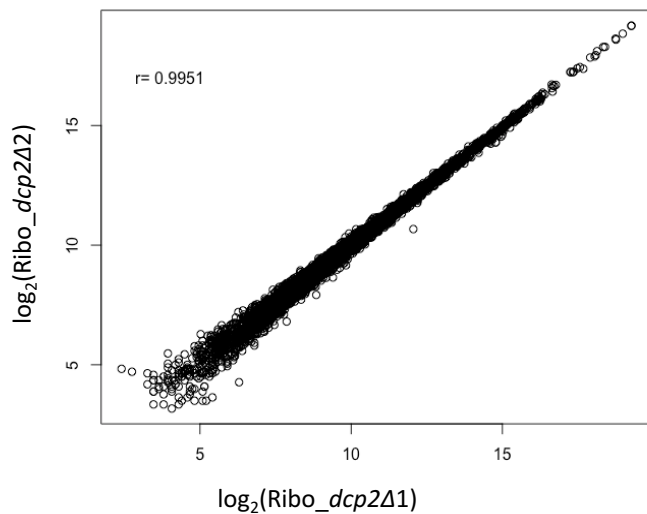

Figure S6 (cont'd)

Supplement: S6 Fig — (A-L) Scatterplots of RNA (A, C, E, G, I, K) or ribosome footprints (B, D, F, H, J, L) read densities (number of reads mapping to each gene’s CDS normalized by the CDS length) for all expressed genes for biological replicates of the following strains: (A-B) HFY114 (WT); (C-D) SYY2353 (scd6Δ); (E-F) QZY126 (dhh1Δ); (G-H) FZY843 (dcp2Δscd6Δ); (I-J) QZY128 (dcp2Δdhh1Δ); (K-L) CFY1016 (dcp2Δ). Pearson correlation coefficients (r) are indicated in each plot. (PDF) [file pgen.1007806.s006.pdf]

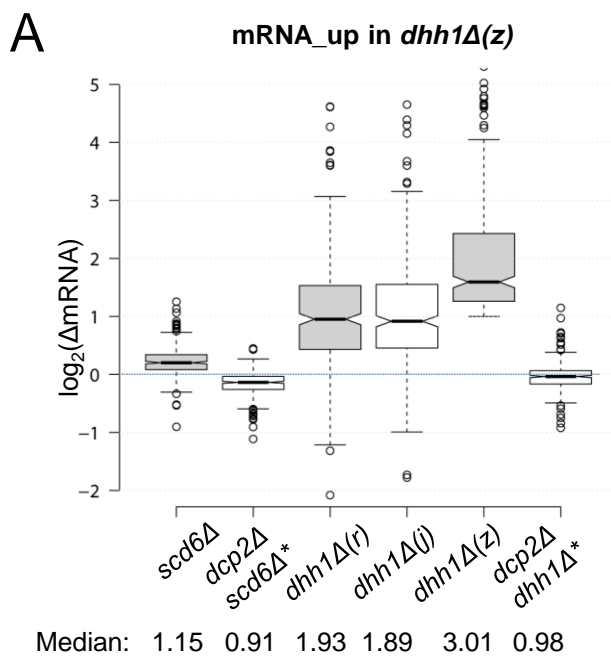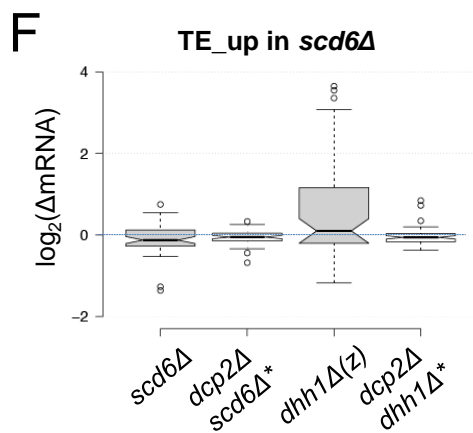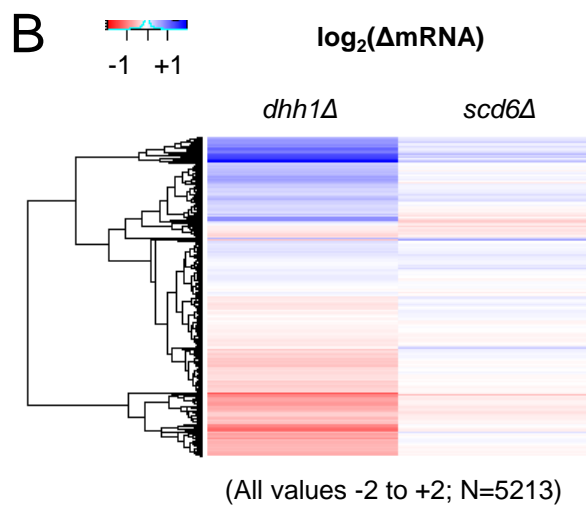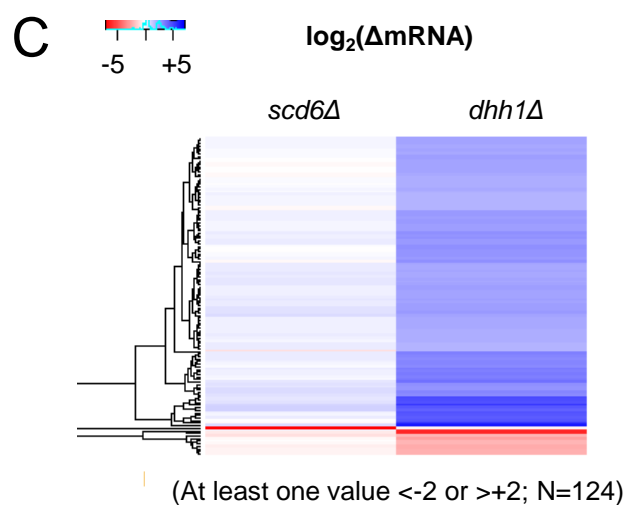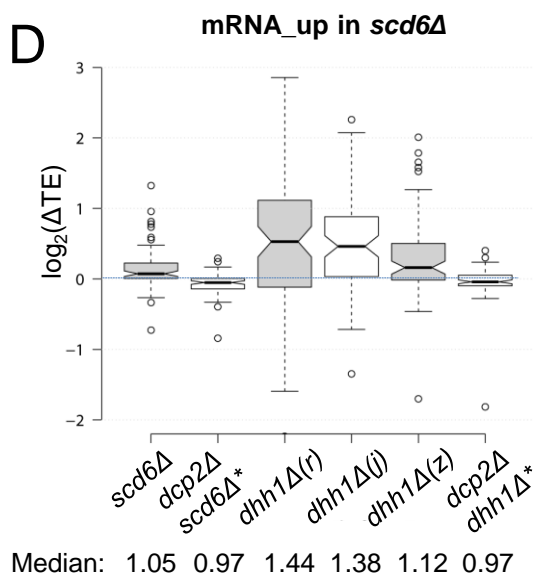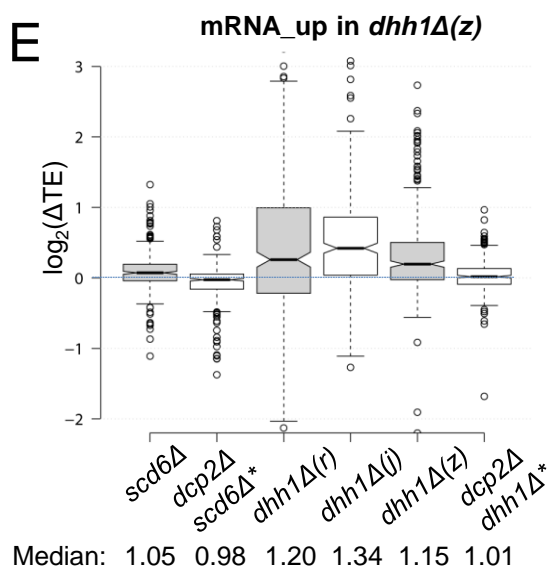

**Figure S7**

Supplement: S7 Fig — (A) Notched box-plots of log2(ΔmRNA) values in the indicated mutants for 346 mRNAs exhibiting ≥2.0-fold increased mRNA abundance in dhh1Δ(z) versus WT cells (at FDR<0.01). TE changes were calculated from the data sets described in Fig 7C. (B-C) Hierachical clustering analysis conducted as in Fig 8A and 8B RNA-Seq data from scd6Δ (SYY2353), dhh1Δ (QZY126, dhh1Δ(z)), and WT (HFY114) strains. Approximately 50 genes were removed for which no data were available in one of the strains, or where the log2(ΔmRNA) value was >4 or <-4 in one of the mutant vs. WT comparisons, after which separate clustering analysis was performed on two sets of mRNAs in which all log2(ΔmRNA) values fell between -2 or +2 (panel B, 5213 mRNAs), or in which the log2(ΔmRNA) value for one of the mutants was < -2 or > +2 (panel C, 124 mRNAs). The color key for log2(ΔmRNA) values is indicated above each analysis. (D-E) Notched box-plots of log2(ΔTE) values in the indicated mutants for the 83 mRNAs analyzed in Fig 7A exhibiting ≥1.4-fold increased mRNA abundance in scd6Δ versus WT cells (D); and for the same 346 mRNAs analyzed in (A), exhibiting ≥2.0-fold increased mRNA abundance in dhh1Δ(z) versus WT cells (E). TE changes were calculated from the data sets described in Fig 7C. (In panels A, C, E & F, the double mutants carry an (*) to indicate that their values have been compared to the dcp2Δ single mutant rather than to WT.) (F) Notched box-plots of log2(ΔmRNA) values in the indicated mutants for 53 mRNAs exhibiting ≥1.33-fold increased TE in scd6Δ versus WT cells described in Fig 7E. The RNA changes were calculated from the indicated data sets described in (A). For panels A, D, E, & F, the changes in mRNA abundance or TE for the relevant group of mRNAs found in each of the indicated mutants were plotted irrespective of whether the changes exhibit statistical significance in that mutant, to allow a coherent comparison of the behavior of the complete cohort of mRNAs across the entire panel of mutants. [file pgen.1007806.s007.pdf]

**A**

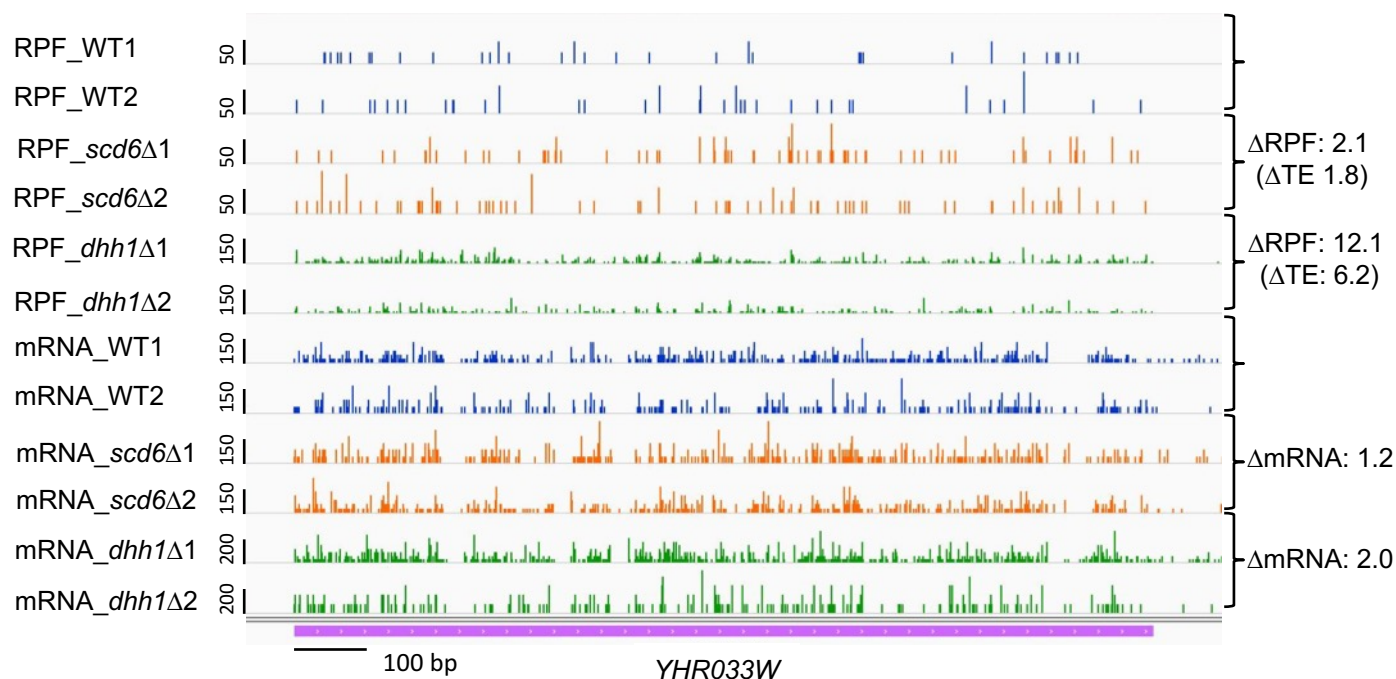

# B

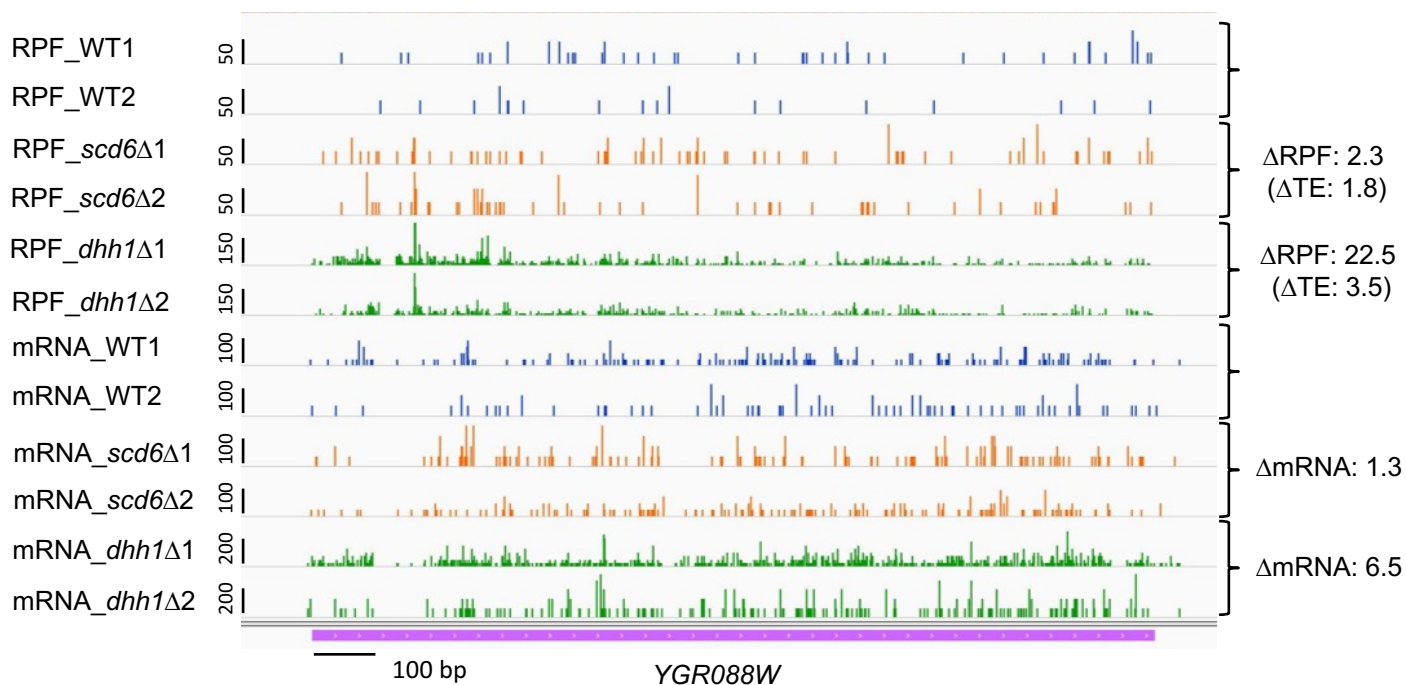

### Figure S8

C

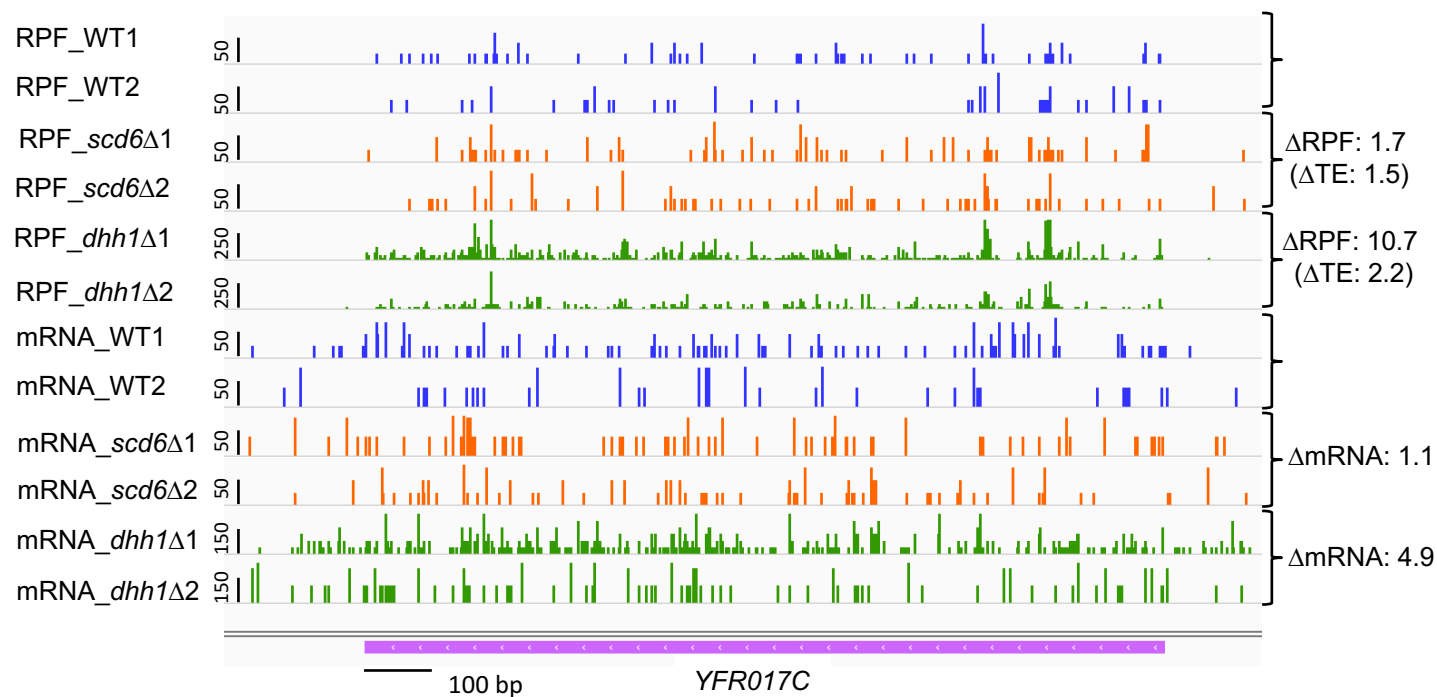

D

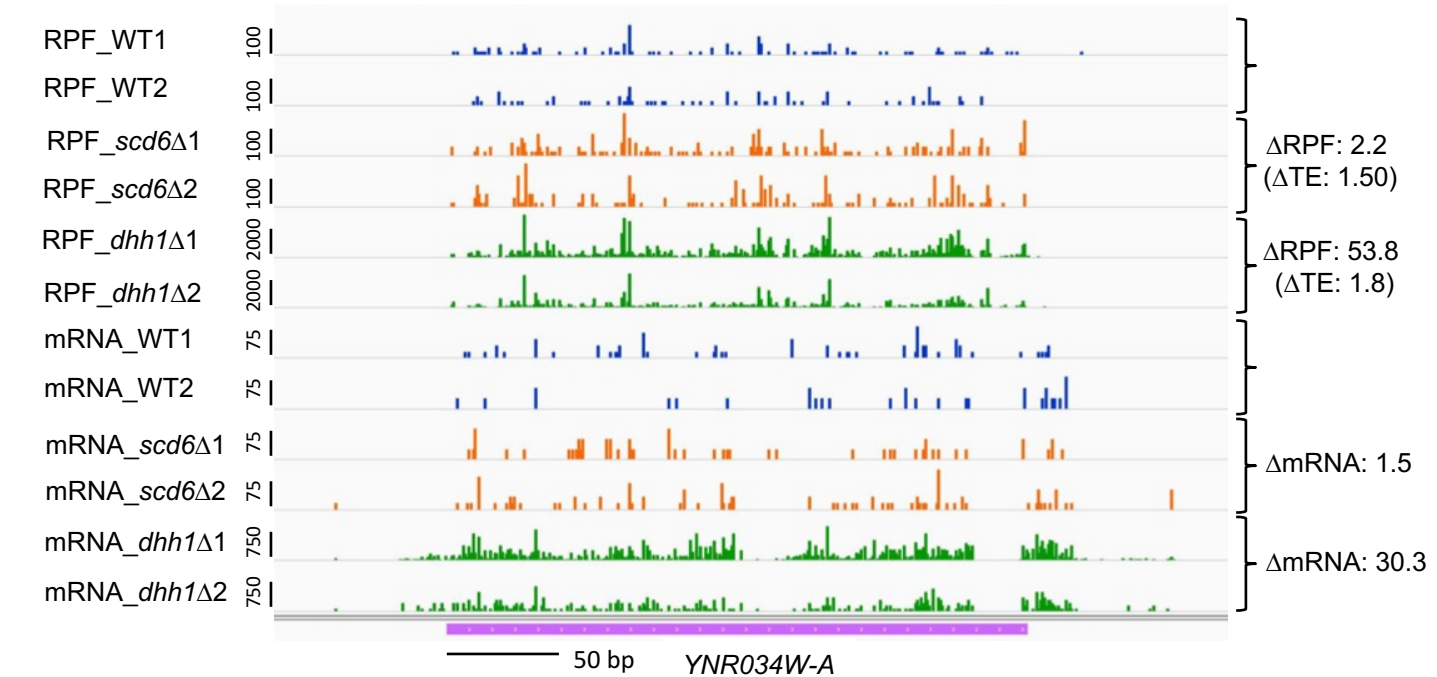

Figure S8 (cont'd)

Supplement: S8 Fig — (A-D) Integrated Genomics Viewer (Broad Institute) displays of ribosome-protected fragments (RPFs) and mRNA reads across the indicated genes from two biological replicates each for WT, scd6Δ and dhh1Δ strains, shown in units of rpkm. Position of the CDS (magenta) is shown at the bottom with the scale in bp; scales of rpkm for each track are on the left, and calculated ΔRPF, ΔmRNA and ΔTE values between each mutant and WT are on the right. (PDF) [file pgen.1007806.s008.pdf]

**A**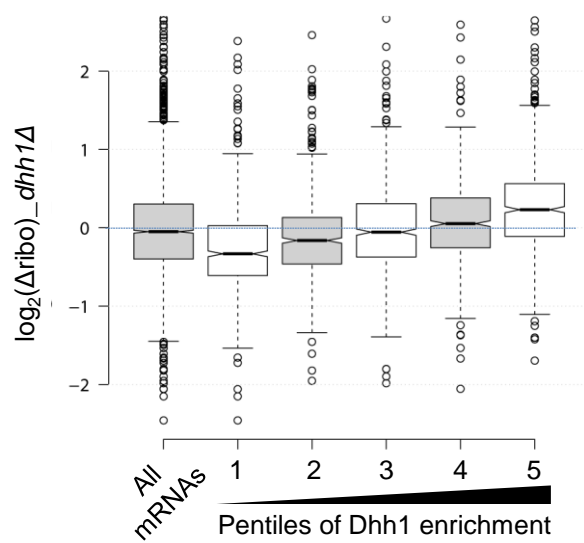**B**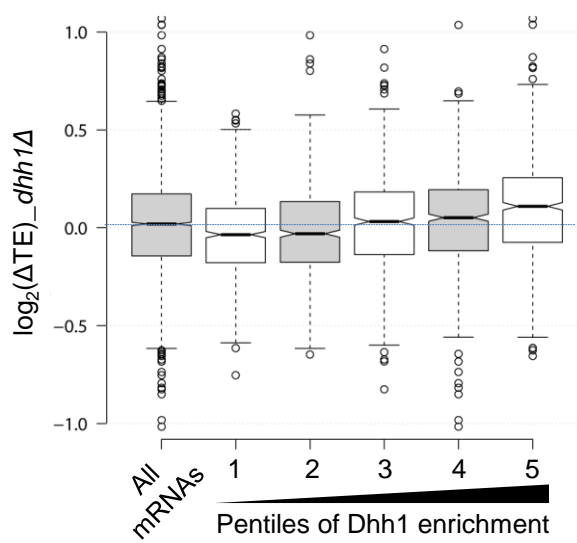**C**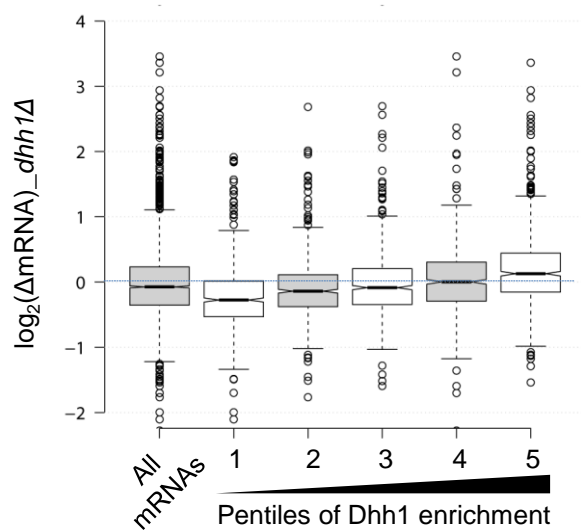**Figure S10**

Supplement: S10 Fig — (A-C) Boxplot analysis of changes in ribosome occupancy (A), TE (B) or mRNA abundance (C) versus Dhh1 RIP-seq enrichment values from Miller et al (2018). The latter were equally divided into five pentiles of 739 genes from lowest to highest enrichment values and plotted against the log2(Δribo) values (A), log2(ΔTE) values (B), or log2(ΔmRNA) values determined by ribosome profiling analysis of dhh1Δ strain (QZY126, dhh1Δ(z)) and WT strain HFY114. The Pearson correlation coefficients for the relationship between log2(Δribo) values (panel A), log2(ΔTE) values (panel B), or log2(ΔmRNA) values (panel C) and Dhh1 enrichment for all mRNAs are 0.31 (P = 3 X 10−81), 0.18 (P = 1 X 10−29), and 0.27 (P = 6 X 10−62), respectively. (PDF) [file pgen.1007806.s010.pdf]

A

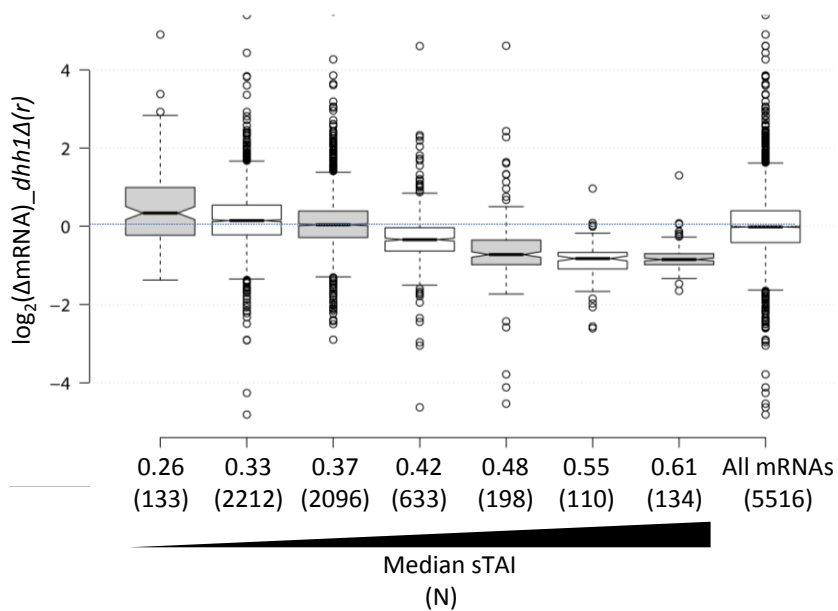

B

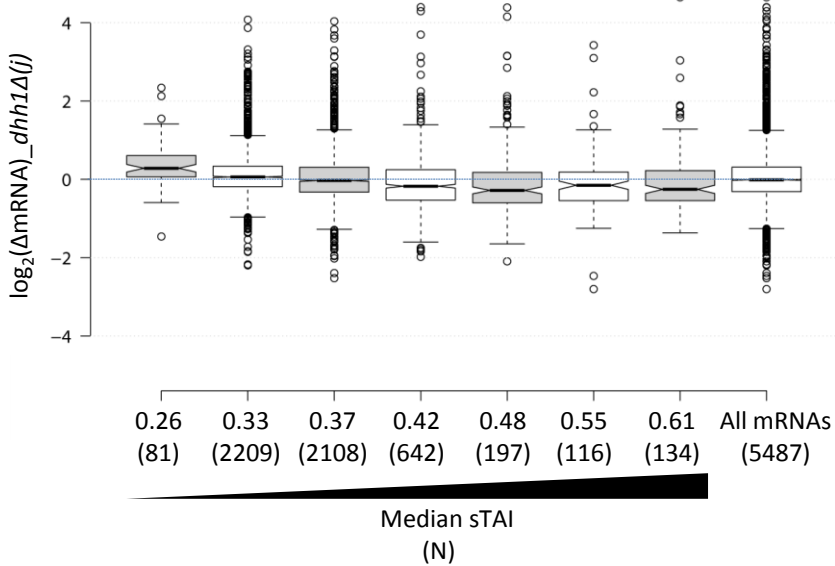

C

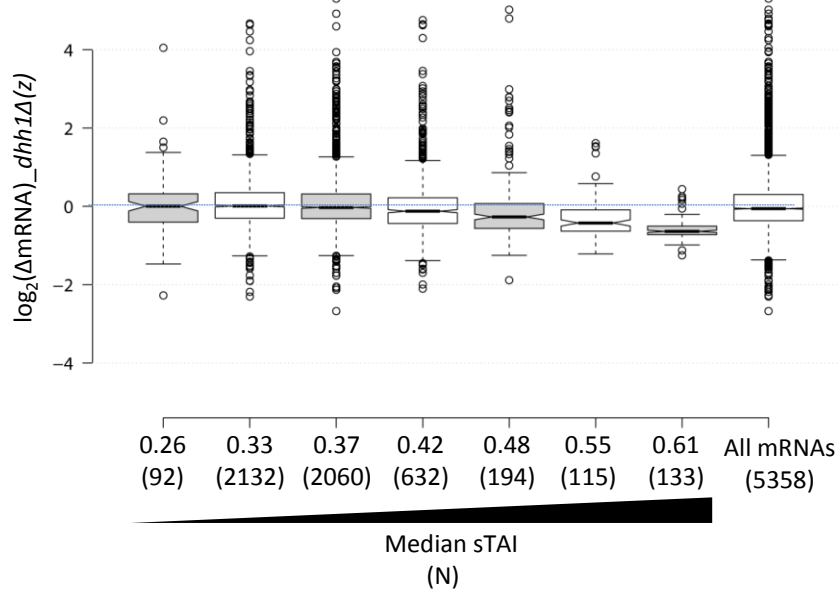

Figure S11

Supplement: S11 Fig — (A-C). Yeast mRNAs were binned by sTAI values, a measure of overall codon optimality [10] and the log2(ΔmRNA) values measured by RNA-Seq in three independent analyses of dhh1Δ vs. WT strains (described in S7A Fig) were displayed in a box-plot for each bin. In all cases, the bin containing the lowest sTAI optimality scores (median of ~0.25) shows greater increases in mRNA expression in the dhh1Δ mutant vs WT compared to the bin containing the highest sTAI optimality scores (median of ~0.61), as observed previously [10]. However, the magnitude of this difference is relatively less for the dhh1Δ datasets from Jungfleisch et al. [37] (B) and the current study (C) compared to that of Radhakrishnan et al. [10] (A). (PDF) [file pgen.1007806.s011.pdf]

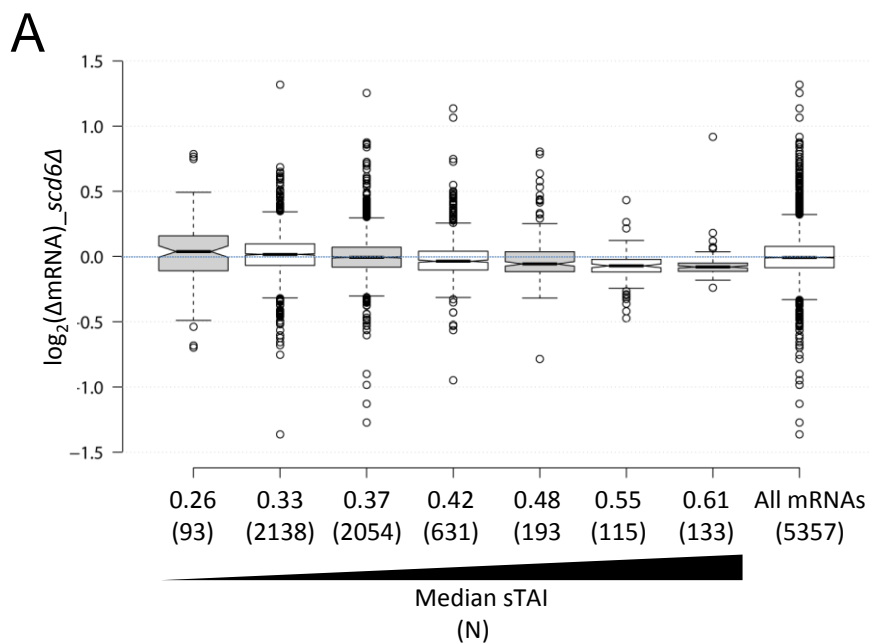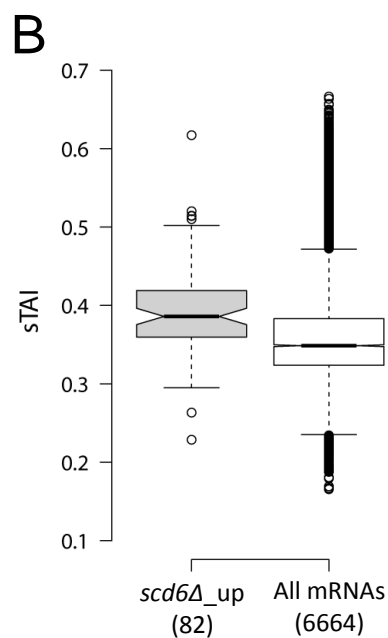

**Figure S12**

Supplement: S12 Fig — (A) Yeast mRNAs were binned by sTAI values as in S11 Fig and the log2(ΔmRNA) values measured by RNA-Seq analysis of scd6Δ (SYY2353) vs. WT (HFY114) cells. The bin containing the lowest sTAI optimality scores (median of ~0.25) shows greater increases in mRNA expression in the scd6Δ mutant vs WT compared to the bin containing the highest sTAI optimality scores (median of ~0.61), as observed previously for a dhh1Δ mutant [10]. (B) The sTAI values are significantly higher for the group of 83 mRNAs found by RNA-Seq to exhibit elevated abundance in scd6Δ strains (SYY2352 and SYY2353) vs. WT strain (HFY114), compared to all mRNAs. (PDF) [file pgen.1007806.s012.pdf]

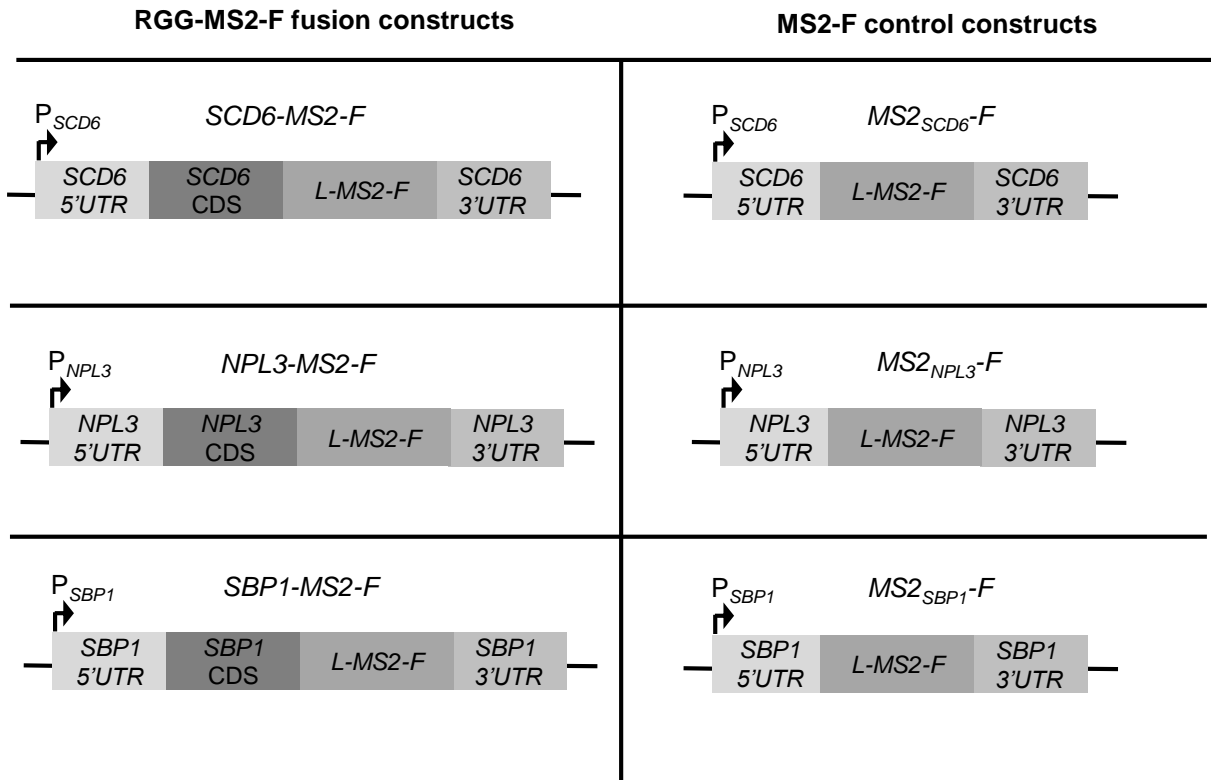

**Figure S13**

Supplement: S13 Fig — Scd6-MS2-F, Npl3-MS2-F, and Spb1-MS-F fusion proteins were expressed under the control of their native promoters and 5’UTR and 3’UTR sequences, with the complete CDS of each protein fused in-frame at the C-terminus to a 5-amino acid linker, followed by the coding sequences for MS2 CP and three FLAG epitopes. The MS2-FLAG control constructs are identical except that they lack the respective Scd6/Npl3/Spb1 CDSs. The fusion protein expression constructs are contained on the following plasmids: ΡSCD6-SCD6-MS2-F (pQZ127), ΡNPL3-NPL3-MS2-F (pQZ125), and ΡSBP1-SBP1-MS2-F (pQZ126). The corresponding MS2-F control constructs are as follows: ΡSCD6-MS2-F (pQZ130), ΡNPL3-MS2-F (pQZ128), and ΡSBP1-MS2-F (pQZ129). (PDF) [file pgen.1007806.s013.pdf]
