# Supplementary material for: Conserved mRNA-granule component Scd6 targets Dhh1 to repress translation initiation and activates Dcp2-mediated mRNA decay in vivo
Source: PLoS Genet. 2018 Dec 7;14(12):e1007806. doi: 10.1371/journal.pgen.1007806 (PMC6307823; doi:10.1371/journal.pgen.1007806)
Supplement: S9 Fig — GO term analysis conducted using web tool Funspec at http://funspec.med.utoronto.ca/ applying Bonferroni correction. The functional categories showing enrichment derive from the MIPS database. k/n/f: number of genes in MIPS category/number of genes in up-regulated list/total number of genes in MIPS category. (A) 346 mRNAs exhibiting ≥2.0-fold increased mRNA abundance in dhh1Δ(z) versus WT cells at FDR<0.01; analyzed in S7A Fig. (B) 83 mRNAs exhibiting ≥1.4-fold increased mRNA abundance in scd6Δ versus WT cells at FDR<0.01; analyzed in Fig 7A. (PDF) [file pgen.1007806.s009.pdf]

A

Category	P-value	k/n/f
metabolism of energy reserves (e.g. glycogen, trehalose) [02.19]	1.73E-09	17/346/56
stress response [32.01]	3.64E-09	29/346/162
sugar, glucoside, polyol and carboxylate catabolism [01.05.02.07]	2.18E-08	19/346/81
C-compound and carbohydrate metabolism [01.05]	4.15E-08	33/346/223
sugar, glucoside, polyol and carboxylate anabolism [01.05.02.04]	9.35E-07	11/346/35

B

Category	P-value	k/n/f
metabolism of energy reserves (e.g. glycogen, trehalose) [02.19]	3.39E-11	11/83/56
sugar, glucoside, polyol and carboxylate catabolism [01.05.02.07]	3.18E-08	10/83/81
C-compound and carbohydrate metabolism [01.05]	1.13E-05	12/83/223

Figure S9
