# Supplementary material for: Conserved mRNA-granule component Scd6 targets Dhh1 to repress translation initiation and activates Dcp2-mediated mRNA decay in vivo
Source: PLoS Genet. 2018 Dec 7;14(12):e1007806. doi: 10.1371/journal.pgen.1007806 (PMC6307823; doi:10.1371/journal.pgen.1007806)
Supplement: S1 Text — Description of procedures employed to analyze the source data found in supporting data files S1-S10 to (i) calculate mean values from replicate determinations of reporter protein or reporter mRNA expression, reporter TE, or changes in these parameters between mutant and WT cells, and conduct statistical analysis of observed differences in the corresponding means, for results in Figs 1, 2, 4, 5, 6, S2, S3 and S5; (ii) conduct statistical analysis of differences in observed mean values of reporter mRNA levels in different polysome gradient fractions for results in Fig 3; (iii) calculate reporter mRNA mean half-lives and conduct statistical analysis of differences in the corresponding mean values for results in S1 Fig. (PDF) [file pgen.1007806.s014.pdf]

S1 Text. Analysis and Explanation of Supporting Data Files

Fig. 1 analysis. Replicate data in supporting file S1 Data were analyzed as follows to obtain results presented in the indicated figure panels. (**B & C**) *GFP* protein expression: *GFP* protein (GFP) and the protein loading control (LC, Prt1) signals on immunoblots were quantified by densitometry and the GFP/LC ratio was calculated for each biological replicate (BR) of Scd6-MS2-F or MS2-F expressing transformants. The mean GFP/LC ratio, and both the standard deviation and S.E.M., were calculated from between 3 and 6 biological replicates of Scd6-MS2-F or MS2-F expressing transformants. An unpaired Student's t-test was performed comparing the mean GFP/LC ratios between the Scd6-MS2-F and MS2-F transformants and the magnitude of the P-values are summarized as: **, P <0.01; *, P <0.05; n.s., non-specific. (**D**) *GFP* mRNA expression: The 2^{-Ct} values from 3 technical replicates (TR) were averaged for *GFP* mRNA, and for *ACT1* mRNA determined from the same RNA sample, and the ratio of *GFP*/*ACT1* 2^{-Ct} values was calculated from the ratio of the resulting mean 2^{-Ct} values. The *GFP*/*ACT1* ratios thus determined from five or more biological replicates were averaged for Scd6-MS2-F or MS2-F expressing transformants, and the mean values and both the standard deviation and S.E.M. were calculated. An unpaired Student's t-test was performed comparing the mean *GFP*/*ACT1* 2^{-Ct} values between the Scd6-MS2-F and MS2-F transformants and magnitudes of the P-values are summarized as: **, P <0.01; *, P <0.05. **(F)** Changes in *GFP* protein or mRNA expression, or in TE values, on tethering Scd6-MS2 vs. MS2 alone: The change in *GFP* protein (Δ*GFP* Protein) was calculated as the ratio of the mean values from panel C for Scd6-MS2-F vs. MS2-F. The propagated S.E.M. for the resulting ratio of means was calculated using the formula: $(X/Y)*(\sqrt{[(SE\_x/x)^2+(SE\_y/y)^2]})$, where X, SE_x, and x are the mean, standard error of the mean, and highest values for Scd6-MS2-F, respectively; and Y, SE_y, and y are the corresponding values for MS2-F. The S.E.M. for Δ*GFP* mRNA was calculated in the same way. Changes in TE of *GFP* mRNA on tethering Scd6-MS2 vs. MS2 alone were calculated as follows, noting that aliquots of cell cultures used for *GFP* protein and *GFP* mRNA were taken from the same biological replicate culture. For each pair of biological replicates expressing Scd6-MS2-F or MS2-F only, the

change in *GFP* protein (Δ*GFP* Protein) and change in *GFP* mRNA (Δ*GFP* mRNA) were calculated as the Scd6-MS2-F/MS2-F ratios from the corresponding *GFP* protein and *GFP* mRNA values (normalized to Prt1 or *ACT1* mRNA as described above), for that pair of transformants. The ratio of the resulting Δ*GFP* Protein to Δ*GFP* mRNA values was calculated to determine the change in TE (ΔTE) on tethering Scd6-MS2-F vs. MS2-F only for that pair of transformants; and the mean ΔTE and S.E.M. was calculated by averaging the ΔTE values for the six different pairs of biological replicates of Scd6-MS2-F and MS2-F transformants.

Fig. 2 analysis. Replicate data in supporting file S2 Data were analyzed as follows to obtain results presented in the indicated figure panels. (**B-C**) *GFP* protein (B) or *GFP* mRNA (C) expression data in each WT and mutant strain were analyzed exactly as described for Fig. 1D and C, respectively, for the WT strain. (**D**) Changes in *GFP* protein or mRNA expression on tethering Scd6-MS2 vs. MS2 alone in each strain were calculated as described above for Fig. 1F for the WT strain. An unpaired Student's t-test was performed comparing the mean Δ*GFP* protein or Δ*GFP* mRNA values between different strains using the propagated S.E.M. values (calculated using the formula in Fig. 1F) and the number (N) of BRs examined in each strain in comparing Scd6-MS2-F to MS2-F transformants. Magnitudes of P-values are summarized as: **, P <0.01; *, P <0.05; n.s., not significant. (**E**) Changes in TE of *GFP* mRNA on tethering Scd6-MS2 vs. MS2 alone in each strain were calculated as described in Fig. 1F for the WT strain. An unpaired Student's t-test was performed comparing the mean ΔTE values between pairs of Scd6-MS2-F and MS2-F transformants determined in each mutant vs. WT, and magnitudes of P-values are summarized as: **, P <0.01; *, P <0.05; n.s., not significant.

Fig. 3 analysis. Replicate data in supporting file S3 Data were analyzed as follows to obtain results presented in the indicated figure panels. **(B-C)** For each of three biological replicate transformants (BR1-

BR3) expressing Scd6-MS2-F or MS2-F alone, 2^{-Ct} values were determined in triplicate (technical replicates TR1-TR3) for *GFP* or *ACT1* mRNA from the RNA isolated from each gradient fraction and averaged. The average 2^{-Ct} value for each fraction was plotted as a proportion of the sum of the average 2^{-Ct} values for all gradient fractions and plotted in Fig. 3 for *GFP1* (panel B) or *ACT1* (panel C). An unpaired Student's t-test was performed comparing the mean proportions of *GFP* mRNA in each fraction for Scd6-MS2-F versus MS2-F transformants, and magnitudes of P-values are summarized as: **, P <0.01; *, P <0.05.

Fig. 4 analysis. Replicate data in supporting file S4 Data were analyzed as follows to obtain results presented in the indicated figure panels. (**A-B, D**) *GFP* protein (A) or *GFP* mRNA (B,D) expression data in WT and *ccr4Δ* (A-B) or *caf1Δ* (D) strains were analyzed exactly as described for Fig. 1C and D, respectively, for the WT strain. (**C-D**) Changes in *GFP* protein or mRNA expression on tethering Scd6-MS2 vs. MS2 alone in each mutant or WT strain (C,D) were analyzed as described for Fig. 2D. Changes in TE of *GFP* mRNA on tethering Scd6-MS2 vs. MS2 alone in each mutant or WT strain (C) were analyzed as described for Fig. 2E.

Fig. 5 analysis. Replicate data in supporting file S5 Data were analyzed as follows to obtain results presented in the indicated figure panels (**B**) Units of β-galactosidase activity measured for three or more biological replicates (BR) of Scd6-MS2-F or MS2-F expressing transformants of each WT or mutant strain were averaged; and an unpaired Student's t-test was performed comparing the mean activities between the Scd6-MS2-F and MS2-F transformants in that strain, with the magnitude of P-values summarized as: **, P <0.01; *, P <0.05; n.s., non-specific. **(C)** *lacZ* mRNA expression data in each strain were analyzed exactly as described for *GFP* mRNA in Fig. 1D for the WT strain. (**D**) Changes in β-galactosidase activity or *lacZ* mRNA expression on tethering Scd6-MS2 vs. MS2 alone in each strain

were analyzed as described for Fig. 2D. (**E**) Changes in TE of *lacZ* mRNA on tethering Scd6-MS2 vs. MS2 alone in each strain were analyzed as described for Fig. 2E.

Fig. 6 analysis. Replicate data in supporting file S6 Data were analyzed as follows to obtain results presented in the indicated figure panels. **(D-G)** *GFP* protein (D & F), *GFP* mRNA (E), and β-galactosidase (G) expression data in WT transformants expressing WT or mutant derivatives of Scd6-MS2-F, or MS2-F alone, were analyzed as described in Fig. 1C, Fig. 1D, and Fig. 5B, respectively.

Fig. S1 analysis. Replicate data in supporting file S7 Data were analyzed as described in S7 Data to obtain results presented in panels B-C.

Fig. S2 analysis. Replicate data in supporting file S8 Data were analyzed as follows to obtain results presented in the indicated figure panels. **(B & D)** *GFP* protein expression data in WT transformants expressing Npl3-MS2-F or MS2-F alone, both expressed from the *NPL3* promoter (B); or Sbp1-MS2-F or MS2-F alone, both expressed from the *SBP1* promoter (D), were analyzed as described in Fig. 1C.

Fig. S3 analysis. Replicate data in supporting file S9 Data were analyzed as follows to obtain results presented in the indicated figure panel. **(B)** *GFP* protein expression data in WT or *dcp2Δ* transformants expressing MS2-F, or containing empty vector, were analyzed as described in Fig. 1C.

Fig. S5 analysis. Replicate data in supporting file S10 Data were analyzed as follows to obtain results presented in the indicated figure panels. **(A-G)** β-galactosidase (A-E) expression data were analyzed as described for Fig. 5B. *lacZ* mRNA (F-G) expression data were analyzed as described for Fig. 1D.
