# Supplementary material for: Correction: Conserved mRNA-granule component Scd6 targets Dhh1 to repress translation initiation and activates Dcp2-mediated mRNA decay in vivo
Source: PLoS Genet. 2019 Jul 23;15(7):e1008299. doi: 10.1371/journal.pgen.1008299 (PMC6650028; doi:10.1371/journal.pgen.1008299)
Supplement: S1 Appendix — Fully annotated DNA sequences of the complete DNA fragments encoding the fusion proteins present in plasmids described in the correct version of Fig 6A, and the multiple sequence alignment delimiting the Scd6 LSm and RGG domains. (PDF) [file pgen.1008299.s001.pdf]

SCD6-MS2

Target sequence description: 474 bp upstream + 1047 scd6 ORF no stop + 480 bp restriction site/linker/ MS2 (no ATG no stop)/3xFLAG/stop + 1232 bp scd6 3'UTR = 3233 bp

Protein sequence: full-length protein M1 to F349

DNA Sequence

TGCTCGTAACAATCTTGGCCTAGCTTCCAGGGTGTCTGTACTTTTACGCAGCATCATATTTTCTCTATTTTT
TGCACATCTTCTTGCTCTTCTTATATTTACCAATTATCTGAAATAGTCACTCGGACCGAGATGCAAAAATC
GGATTTAAAACTCGATCCGAGGAGAACACCAAATGACACAACTTCTATCAATGCAATTGCCCTCTAAGTA
TCATAGATGCCGCTATTTTAAATGACAGTGCAATCGATTTCCATATTAAATGGATAAAGGTGTTTATTATG
AAGATACCCATTACTGACGTATATGTAATGATTCCTCAGTCCAGTACCATAGTCCCGTCACCTTACACGGT
GGAAAAATTAAACTGTACCCGCGATTGTAAAGAATAACGTGGAGGCAGTCAGAAAGAACAATAAACGA
ATAGTGAGGATGTCGTAGAAGAAGCATTCATCGAAAAACAGCAGCAAGGCAATGTCGCAGTACATCGG
TAAAACTATTTCTTTAATCTCTGTGACTGACAACAGATATGTGGGGCTGTTAGAAGATATTGACTCTGAA
AAGGGTACCGTGACTTTGAAAGAAGTTCGCTGTTTTGGTACAGAAGGTCGCAAGAACTGGGGTCCTGA
AGAAATTTATCCGAATCCTACGGTATACAATTCTGTAAAGTTCAACGGCAGTGAAGTCAAGGATTTAAGC
ATTTTAGATGCTAACATCAATGACATACAGCCGGTTGTTCCTCAAATGATGCCACCCGCTTCACAATTCCC
TCCTCAACAAGCTCAATCTCCACCCCAGGCTCAAGCTCAAGCACACGTGCAAACAAACCCCCAAGTTCCA
AAGCCCGAATCCAATGTGCCAGCAGCTGTCGCTGGATATGGTGTTTACACCCCAACTTCGACAGAAACC
GCTACTGCTAGTATGAATGATAAGAGCACTCCTCAAGACACCAATGTAAACTCGCAAAGTAGGGAAAGA
GGTAAAAATGGTGAAAATGAGCCAAAATATCAAAGAAACAAGAATAGATCAAGTAATCGCCCTCCTCAA
TCCAACCGCAATTTCAAAGTCGATATTCCGAATGAAGATTTTGACTTTCAATCAAATAATGCAAAATTCAC
GAAAGGTGATTCCACTGATGTGGAAAAAGAAAAAGAATTAGAATCAGCTGTTCACAAGCAGGATGAAT
CTGATGAGCAGTTTTATAATAAAAAATCGTCTTTTTTCGACACCATCTCCACTTCTACTGAAACTAATACC
AATATGAGATGGCAAGAAGAAAAAATGTTGAACGTTGACACCTTTGGACAAGCTTCTGCCAGACCAAGA
TTTCACTCTAGAGGCCTCGGTCGTGGGCGTGGAAATTATAGGGGAAACAGAGGAAACAGAGGAAGAG
GCGGCCAACGTGGAAACTACCAAAACAGAAATAACTACCAAAATGATAGTGGCGCCTATCAGAACCAA
AACGACTCGTACAGCAGACCAGCCAACCAGTTTTCGCAACCTCCTTCCAACGTTGAATTTCTCGAGGGTG
GTGGTGGTGGTTCTGCTTCTAACTTTACTCAGTTCGTTCTCGTCGACAATGGCGGAACTGGCGACGTGAC
TGTCGCCCCAAGCAACTTCGCTAACGGGGTCGCTGAATGGATCAGCTCTAACTCGCGATCACAGGCTTA
CAAAGTAACCTGTAGCGTTCGTCAGAGCTCTGCGCAGAATCGCAAATACACCATCAAAGTCGAGGTGCC
TAAAGTGGCAACCCAGACTGTTGGTGGTGAAGAGCTTCCTGTAGCCGGATGGAGATCTTACTTAAATAT
GGAACTAACCATTCCAATTTTCGCCACGAATTCCGACTGCGAGCTTATTGTTAAGGCAATGCAAGGTCTC
CTAAAAGATGGAAACCCGATTCCCTCGGCCATCGCAGCAAACTCCGGCATCTACGACTACAAAGACCAT
GACGGTGATTATAAAGATCATGACATCGACTACAAGGATGACGATGACAAGTAAAATGATGTTTCTATG
TAAATTAAGTATATCATTCAGTATGTACATACAAAAATCAAATTAATAGTATTATAGTCCGCGCCTCTGGA
TAGTATTATTTTAGTGCGTATCGCAAAATATGAATAGCACAGAGGTTCATTATTATGTTCTGAGTATGAG
GACGAGGTAATACATACCTTAAAATCTTACATCATGTGGATAGCATTTATGGCTTCTTCCATCCGGCCGT
ACGCTTCTGTTCCTGTCCCTAGCCATAGCTTTTCTCATATCAAAAGCATCTTTATTGTCATCTACTTTTTTCA
GGGTATCTGGATACCCATCTTCAGCTGAGTTATAATATCCCACCACTCTCCTGTAGACACTGTATCCCAAT
TTTTCGTAAAGTTTGATGGCCAATTGGTTATTGCACTTGACGAAAAGATCGATAAAGTTGACTTCATGGG

GCATGACATCGGTCATGGTCTCCAAAGTATTACATAACTTCGAAGCTAGCGATATTCGGCGGAATCTTGG
AGCTACTGTCACTGCAGTTATATGAGTGTGCCACTCCGTGGTCTTGCCTTCTGTTTTTGCCATCATGTAAC
CAGAGATATTATGCTTAAATGTAGGATCAACAGTCATTTCTGAACTTTTGAAAAAAAGGTCTGGCCATAT
TATCATGTATTCAAAATAAAACTCTAAAGGGAAGTTTTCTGTTAAGATATCAAGGTTGACATTGTTAGTTT
TGAATAAATCGACAGGTTCAAAAGGCTGAATTGTTGTCATTTCTCGAATCCTCCTGTCTCTTTCCTTGAAT
ATTCTCAATGTTCCACTATCTTTCACATAAGCAATATTGTGAACTATATATACTTCATTAGATATTGCTTCA
ACATTAATATTAATAAAAAGGACATGCATAAAGTAACATCCGAACATCTCCAAACACCCATACATATTTA
TATTATCGACGAGGGAATTTTTTTCTTCCCAGGCCCACCAACGTGCGCCCACCGCTATGTTTTTTGCGCCT
AGGTAGGTGGGCTAGCTTCTATGTATCAAAGCTCCTTCTGCGCAGCCGAGGTTTTGCTAAGCACTACCGC
ATTGGAATGACTGTGTTTGTAGGAGCTCCTTGTCTCCACGCTTTCGCAATTTTCAAGATCGAGATACTATT
AGTGATGGTAAGGTTATGAAGCTTACATGTTTAATATTGTAGATTCCAGAAGCAGGTAAGAACAACAAA
CTACAAGAAAAGGGTA

Legend

1. TGCTCG ……… AAGGCA = SCD6 native promoter (474 bp)

2. ATGTCGCAG ……. GTTGAATTT = Scd6 cds minus stop codon (1047); start codon highlighted yellow

3. GGTGGTGGTGGTGGTTCT = linker

4. GCTTCTAACTTTACTCAGTTCGTTCTCGTCGACAATGGCGGAACTGGCGACGTGACTGTCGCCCC AAGCAACTTCGCTAACGGGGTCGCTGAATGGATCAGCTCTAACTCGCGATCACAGGCTTACAAA GTAACCTGTAGCGTTCGTCAGAGCTCTGCGCAGAATCGCAAATACACCATCAAAGTCGAGGTGC CTAAAGTGGCAACCCAGACTGTTGGTGGTGAAGAGCTTCCTGTAGCCGGATGGAGATCTTACTT AAATATGGAACTAACCATTCCAATTTTCGCCACGAATTCCGACTGCGAGCTTATTGTTAAGGCAA TGCAAGGTCTCCTAAAAGATGGAAACCCGATTCCCTCGGCCATCGCAGCAAACTCCGGCATCTA C = MS2 minus start minus stop (387 bp)

5. GACTACAAAGACCATGACGGTGATTATAAAGATCATGACATCGACTACAAGGATGACGATGAC AAGTAA = 3xFlag tag + Stop codon

6. AATGAT ….. AAGGGTA = Scd6 3’ UTR (1232 bp)

Notes

Lsm domain Q3-D78

CAGTACATCGGTAAAACTATTTCTTTAATCTCTGTGACTGACAACAGATATGTGGGGCTGTTAGAAGATA
TTGACTCTGAAAAGGGTACCGTGACTTTGAAAGAAGTTCGCTGTTTTGGTACAGAAGGTCGCAAGAACT
GGGGTCCTGAAGAAATTTATCCGAATCCTACGGTATACAATTCTGTAAAGTTCAACGGCAGTGAAGTCA
AGGATTTAAGCATTTTAGAT

RGG boxes S287-N318

TCTAGAGGCCTCGGTCGTGGGCGTGGAAATTATAGGGGAAACAGAGGAAACAGAGGAAGAGGCGGCC
AACGTGGAAACTACCAAAACAGAAATAAC

ΔLsm-SCD6-MS2

Description: YCplac111 - 474 bp upstream – SCD6 cds (minus 228 bp near the N-terminus) - linker – MS2CP (minus ATG minus stop) – 3xFlag – stop (TAA) – 3'UTR Scd6 (1232 bp) – Ycplac111

Protein sequence alteration: deletion from Q3 to D78 (76 aa, corresponding to Lsm domain (see Scd6 multiple sequence alignment).

DNA Sequence

GCATGCCTGCAGGTCGACTCTAGTTGCTCGTAACAATCTTGGCCTAGCTTCCAGGGTGTCTGTAC
TTTTACGCAGCATCATATTTTCTCTATTTTTTGCACATCTTCTTGCTCTTCTTATATTTACCAATTAT
CTGAAATAGTCACTCGGACCGAGATGCAAAAATCGGATTTAAAACTCGATCCGAGGAGAACAC
CAAATGACACAACTTCTATCAATGCAATTGCCCTCTAAGTATCATAGATGCCGCTATTTTAAATG
ACAGTGCAATCGATTTCCATATTAAATGGATAAAGGTGTTTATTATGAAGATACCCATTACTGAC
GTATATGTAATGATTCCTCAGTCCAGTACCATAGTCCCGTCACCTTACACGGTGGAAAAATTAAA
CTGTACCCGCGATTGTAAAGAATAACGTGGAGGCAGTCAGAAAGAACAATAAACGAATAGTGA
GGATGTCGTAGAAGAAGCATTCATCGAAAAACAGCAGCAAGGCAATGTCGGCTAACATCAATG
ACATACAGCCGGTTGTTCCTCAAATGATGCCACCCGCTTCACAATTCCCTCCTCAACAAGCTCAA
TCTCCACCCCAGGCTCAAGCTCAAGCACACGTGCAAACAAACCCCCAAGTTCCAAAGCCCGAAT
CCAATGTGCCAGCAGCTGTCGCTGGATATGGTGTTTACACCCCAACTTCGACAGAAACCGCTAC
TGCTAGTATGAATGATAAGAGCACTCCTCAAGACACCAATGTAAACTCGCAAAGTAGGGAAAG
AGGTAAAAATGGTGAAAATGAGCCAAAATATCAAAGAAACAAGAATAGATCAAGTAATCGCCC
TCCTCAATCCAACCGCAATTTCAAAGTCGATATTCCGAATGAAGATTTTGACTTTCAATCAAATA
ATGCAAAATTCACGAAAGGTGATTCCACTGATGTGGAAAAAGAAAAAGAATTAGAATCAGCTG
TTCACAAGCAGGATGAATCTGATGAGCAGTTTTATAATAAAAAATCGTCTTTTTTCGACACCATC
TCCACTTCTACTGAAACTAATACCAATATGAGATGGCAAGAAGAAAAAATGTTGAACGTTGACA
CCTTTGGACAAGCTTCTGCCAGACCAAGATTTCACTCTAGAGGCCTCGGTCGTGGGCGTGGAAA
TTATAGGGGAAACAGAGGAAACAGAGGAAGAGGCGGCCAACGTGGAAACTACCAAAACAGAA
ATAACTACCAAAATGATAGTGGCGCCTATCAGAACCAAAACGACTCGTACAGCAGACCAGCCAA
CCAGTTTTCGCAACCTCCTTCCAACGTTGAATTTCTCGAGGGTGGTGGTGGTGGTTCTGCTTCTA
ACTTTACTCAGTTCGTTCTCGTCGACAATGGCGGAACTGGCGACGTGACTGTCGCCCCAAGCAA
CTTCGCTAACGGGGTCGCTGAATGGATCAGCTCTAACTCGCGATCACAGGCTTACAAAGTAACC
TGTAGCGTTCGTCAGAGCTCTGCGCAGAATCGCAAATACACCATCAAAGTCGAGGTGCCTAAAG
TGGCAACCCAGACTGTTGGTGGTGAAGAGCTTCCTGTAGCCGGATGGAGATCTTACTTAAATAT
GGAACTAACCATTCCAATTTTCGCCACGAATTCCGACTGCGAGCTTATTGTTAAGGCAATGCAA
GGTCTCCTAAAAGATGGAAACCCGATTCCCTCGGCCATCGCAGCAAACTCCGGCATCTACGACT
ACAAAGACCATGACGGTGATTATAAAGATCATGACATCGACTACAAGGATGACGATGACAAGT
AAAATGATGTTTCTATGTAAATTAAGTATATCATTCAGTATGTACATACAAAAATCAAATTAATA
GTATTATAGTCCGCGCCTCTGGATAGTATTATTTTAGTGCGTATCGCAAAATATGAATAGCACAG
AGGTTCATTATTATGTTCTGAGTATGAGGACGAGGTAATACATACCTTAAAATCTTACATCATGT
GGATAGCATTTATGGCTTCTTCCATCCGGCCGTACGCTTCTGTTCCTGTCCCTAGCCATAGCTTTT
CTCATATCAAAAGCATCTTTATTGTCATCTACTTTTTTCAGGGTATCTGGATACCCATCTTCAGCT
GAGTTATAATATCCCACCACTCTCCTGTAGACACTGTATCCCAATTTTTCGTAAAGTTTGATGGCC

AATTGGTTATTGCACTTGACGAAAAGATCGATAAAGTTGACTTCATGGGGCATGACATCGGTCA
TGGTCTCCAAAGTATTACATAACTTCGAAGCTAGCGATATTCGGCGGAATCTTGGAGCTACTGT
CACTGCAGTTATATGAGTGTGCCACTCCGTGGTCTTGCCTTCTGTTTTTGCCATCATGTAACCAG
AGATATTATGCTTAAATGTAGGATCAACAGTCATTTCTGAACTTTTGAAAAAAAGGTCTGGCCAT
ATTATCATGTATTCAAAATAAAACTCTAAAGGGAAGTTTTCTGTTAAGATATCAAGGTTGACATT
GTTAGTTTTGAATAAATCGACAGGTTCAAAAGGCTGAATTGTTGTCATTTCTCGAATCCTCCTGT
CTCTTTCCTTGAATATTCTCAATGTTCCACTATCTTTCACATAAGCAATATTGTGAACTATATATAC
TTCATTAGATATTGCTTCAACATTAATATTAATAAAAAGGACATGCATAAAGTAACATCCGAACA
TCTCCAAACACCCATACATATTTATATTATCGACGAGGGAATTTTTTTCTTCCCAGGCCCACCAAC
GTGCGCCCACCGCTATGTTTTTTGCGCCTAGGTAGGTGGGCTAGCTTCTATGTATCAAAGCTCCT
TCTGCGCAGCCGAGGTTTTGCTAAGCACTACCGCATTGGAATGACTGTGTTTGTAGGAGCTCCT
TGTCTCCACGCTTTCGCAATTTTCAAGATCGAGATACTATTAGTGATGGTAAGGTTATGAAGCTT
ACATGTTTAATATTGTAGATTCCAGAAGCAGGTAAGAACAACAAACTACAAGAAAAGGGTA
CCCGGGTACCGAGCTCGAATTCACTGGCCGTCGTTTTACAACGTCGTGACTGGGAAAACCCTGG

Legend

1. GCATGC = SphI restriction site
2. CTGCAGGTCGACTCTAGT = vector DNA
3. TGCTCG ……… AAGGCA = SCD6 native promoter (474 bp)
4. ATGTCGGCTA ……. GAATTT = Scd6 cds with 228 bp Lsm deletion minus stop codon (819 bp); start codon highlighted yellow
5. GGTGGTGGTGGTGGTTCT = linker
6. GCTTCTAACTTTACTCAGTTCGTTCTCGTCGACAATGGCGGAACTGGCGACGTGACTGT
CGCCCCAAGCAACTTCGCTAACGGGGTCGCTGAATGGATCAGCTCTAACTCGCGATCAC
AGGCTTACAAAGTAACCTGTAGCGTTCGTCAGAGCTCTGCGCAGAATCGCAAATACACC
ATCAAAGTCGAGGTGCCTAAAGTGGCAACCCAGACTGTTGGTGGTGAAGAGCTTCCTG
TAGCCGGATGGAGATCTTACTTAAATATGGAACTAACCATTCCAATTTTCGCCACGAAT
TCCGACTGCGAGCTTATTGTTAAGGCAATGCAAGGTCTCCTAAAAGATGGAAACCCGA
TTCCCTCGGCCATCGCAGCAAACTCCGGCATCTAC = MS2 (387 bp)
7. GACTACAAAGACCATGACGGTGATTATAAAGATCATGACATCGACTACAAGGATGACG
ATGACAAGTAA = 3xFlag tag + Stop codon
8. AATGAT ….. AAGGGTA = Scd6 3’ UTR (1232 bp)
9. CCCGGG = SmaI restriction site
10. TACCGAGCTCGAATTCACTGGCCGTCGTTTTACAACGTCGTGACTGGGAAAACCCTGGC
GTGAA = vector DNA

Deleted fragment corresponding to Q3-D78 (in original WT sequence; missing from construct above)
CAGTACATCGGTAAAACTATTTCTTTAATCTCTGTGACTGACAACAGATATGTGGGGCTGTTAGA
AGATATTGACTCTGAAAAGGGTACCGTGACTTTGAAAGAAGTTCGCTGTTTTGGTACAGAAGGT
CGCAAGAACTGGGGTCCTGAAGAAATTTATCCGAATCCTACGGTATACAATTCTGTAAAGTTCA
ACGGCAGTGAAGTCAAGGATTTAAGCATTTTAGAT

Packing List

GenScript
The Biology CRO

GenScript USA Inc.
860 Centennial Ave, Piscataway, NJ 08854, USA
Tel:732-885-9188 Fax:732-210-0262
E-mail: order@genscript.com

Ship To: Quira Zeidan
National Institutes of Health (NIH) - MD
Bldg 6, BETHMC, 6 Center Drive
Bethesda, MD 20892, US
3014356962

Bill To: Quira Zeidan
National Institutes of Health (NIH) - MD
2115 E Jefferson St., MSC 8500 Suite 4B 432
Bethesda, MD 20892, US
3014356962

Customer No.	PO#	Order#	Order Date	Ship Via	Shipping Date	Terms	Total Weight	# Of Packages
**204455**	4420227	U7551BL150	2016-12-16	FedEx Domestic Standard Overnight		Net 30	0.5 (lb)	1/1

Item	Catalog number	Order Qty	Ship Qty	Description
**U7551BL150_1**	SC1023	1	1	DeltaLsm-Scd6-MS2: Target Insert Name: DeltaLsm-Scd6-MS2, Template information: Insert name: Scd6-MS2, Cloning site: SphI-SmaI, Vector name: YCpLac111, Vector size(bp): 6112, Resistance: Ampicillin, Copy number: Low, Target vector: Same as template,<br>Plasmid preparation: Standard delivery: 4 µg (Free of charge)

ALL REAGENTS ARE FOR RESEARCH USE ONLY AND ARE NOT INTENDED FOR DIAGNOSTIC OR THERAPEUTIC USE. GENSCRIPT MAKES NO CLAIMS TO THE PRODUCT'S ABILITY TO FUNCTION IN THE SPECIFIC APPLICATION OF THE CUSTOMER.

This is the partial shipment of your #U7551BL150

```
                                          10         20         30         40         50         60         70         80         90         100        110
                                          CACAGGAaACAGCTATGACCATGATTACGCCAAGCTTGCATGCCTGCAGGTCGACTCTAGTTGCTCGTAACAATCTTGGCCTAGCTTCCAGGGTGTCTGTACTTTTACGCAGCATCATA
A01-X82565-U7551BL150-1-M13R-88.ab1(1>817)  →  CACAGGAaACAGCTATGACCATGATTACGCCAAGCTTGCATGCCTGCAGGTCGACTCTAGTTGCTCGTAACAATCTTGGCCTAGCTTCCAGGGTGTCTGTACTTTTACGCAGCATCATA
U7551BL150-1.seq(1>3036)                    →                                        GCATGCCTGCAGGTCGACTCTAGTTGCTCGTAACAATCTTGGCCTAGCTTCCAGGGTGTCTGTACTTTTACGCAGCATCATA
                                                                                     SphI

                                          120        130        140        150        160        170        180        190        200        210        220        230
                                          TTTTCTCTATTTTTTGCACATCTTCTTGCTCTTCTTATATTTACCAATTATCTGAAATAGTCACTCGGACCGAGATGCAAAAATCGGATTTAAAACTCGATCCGAGGAGAACACCAAAT
A01-X82565-U7551BL150-1-M13R-88.ab1(1>817)  →  TTTTCTCTATTTTTTGCACATCTTCTTGCTCTTCTTATATTTACCAATTATCTGAAATAGTCACTCGGACCGAGATGCAAAAATCGGATTTAAAACTCGATCCGAGGAGAACACCAAAT
U7551BL150-1.seq(1>3036)                    →  TTTTCTCTATTTTTTGCACATCTTCTTGCTCTTCTTATATTTACCAATTATCTGAAATAGTCACTCGGACCGAGATGCAAAAATCGGATTTAAAACTCGATCCGAGGAGAACACCAAAT

                                          240        250        260        270        280        290        300        310        320        330        340        350
                                          GACACAACTTCTATCAATGCAATTGCCCTCTAAGTATCATAGATGCCGCTATTTTAAATGACAGTGCAATCGATTTCCATATTAAATGGATAAAGGTGTTTATTATGAAGATACCCATT
A01-X82565-U7551BL150-1-M13R-88.ab1(1>817)  →  GACACAACTTCTATCAATGCAATTGCCCTCTAAGTATCATAGATGCCGCTATTTTAAATGACAGTGCAATCGATTTCCATATTAAATGGATAAAGGTGTTTATTATGAAGATACCCATT
U7551BL150-1.seq(1>3036)                    →  GACACAACTTCTATCAATGCAATTGCCCTCTAAGTATCATAGATGCCGCTATTTTAAATGACAGTGCAATCGATTTCCATATTAAATGGATAAAGGTGTTTATTATGAAGATACCCATT

                                          360        370        380        390        400        410        420        430        440        450        460        470
                                          ACTGACGTATATGTAATGATTCCTCAGTCCAGTACCATAGTCCCGTCACCTTACACGGTGGAAAAATTAAACTGTACCCGCGATTGTAAAGAATAACGTGGAGGCAGTCAGAAAGAACA
A01-X82565-U7551BL150-1-M13R-88.ab1(1>817)  →  ACTGACGTATATGTAATGATTCCTCAGTCCAGTACCATAGTCCCGTCACCTTACACGGTGGAAAAATTAAACTGTACCCGCGATTGTAAAGAATAACGTGGAGGCAGTCAGAAAGAACA
U7551BL150-1.seq(1>3036)                    →  ACTGACGTATATGTAATGATTCCTCAGTCCAGTACCATAGTCCCGTCACCTTACACGGTGGAAAAATTAAACTGTACCCGCGATTGTAAAGAATAACGTGGAGGCAGTCAGAAAGAACA

                                          480        490        500        510        520        530        540        550        560        570        580        590
                                          ATAAACGAATAGTGAGGATGTCGTAGAAGAAGCATTCATCGAAAAACAGCAGCAAGGCAATGTCGGCTAACATCAATGACATACAGCCGGTTGTTCCTCAAATGATGCCACCCGCTTCA
A01-X82565-U7551BL150-1-M13R-88.ab1(1>817)  →  ATAAACGAATAGTGAGGATGTCGTAGAAGAAGCATTCATCGAAAAACAGCAGCAAGGCAATGTCGGCTAACATCAATGACATACAGCCGGTTGTTCCTCAAATGATGCCACCCGCTTCA
U7551BL150-1.seq(1>3036)                    →  ATAAACGAATAGTGAGGATGTCGTAGAAGAAGCATTCATCGAAAAACAGCAGCAAGGCAATGTCGGCTAACATCAATGACATACAGCCGGTTGTTCCTCAAATGATGCCACCCGCTTCA
                                                                                                         start   SCD6 cds deleted Lsm domain

                                          600        610        620        630        640        650        660        670        680        690        700        710
                                          CAATTCCCTCCTCAACAAGCTCAATCTCCACCCCAGGCTCAAGCTCAAGCACACGTGCAAACAAACCCCCAAGTTCCAAAGCCCGAATCCAATGTGCCAGCAGCTGTCGCTGGATATGG
A01-X82565-U7551BL150-1-M13R-88.ab1(1>817)  →  CAATTCCCTCCTCAACAAGCTCAATCTCCACCCCAGGCTCAAGCTCAAGCACACGTGCAAACAAACCCCCAAGTTCCAAAGCCCGAATCCAATGTGCCAGCAGCTGTCGCTGGATATGG
U7551BL150-1.seq(1>3036)                    →  CAATTCCCTCCTCAACAAGCTCAATCTCCACCCCAGGCTCAAGCTCAAGCACACGTGCAAACAAACCCCCAAGTTCCAAAGCCCGAATCCAATGTGCCAGCAGCTGTCGCTGGATATGG
B09-X82565-U7551BL150-1-1-1-SEQ1.ab1(1>783) →                                 CCCAGGCTCAAGCTCAAGCACACGTGCAAACAAACCCCCAAGTTCCAAAGCCCGAATCCAATGTGCCAGCAGCTGTCGCTGGATATGG

                                          720        730        740        750        760        770        780        790        800        810        820        830
                                          TGTTTACACCCCAACTTCGACAGAAACCGCTACTGCTAGTATGAATGATAAGAGCACTCCTCAAGACACCAATGTAAACTCGCAAAGTAGGGAAAGAGGTAAAAATGGTGAAAATGAGC
A01-X82565-U7551BL150-1-M13R-88.ab1(1>817)  →  TGTTTACACCCCAACTTCGACAGAAACCGCTACTGCTAGTATGAATGATAAGAGCACTCCTCAAGACACCAATGTAAACTCGCAAAGTAGGGAAAGAGGTAAA
U7551BL150-1.seq(1>3036)                    →  TGTTTACACCCCAACTTCGACAGAAACCGCTACTGCTAGTATGAATGATAAGAGCACTCCTCAAGACACCAATGTAAACTCGCAAAGTAGGGAAAGAGGTAAAAATGGTGAAAATGAGC
B09-X82565-U7551BL150-1-1-1-SEQ1.ab1(1>783) →  TGTTTACACCCCAACTTCGACAGAAACCGCTACTGCTAGTATGAATGATAAGAGCACTCCTCAAGACACCAATGTAAACTCGCAAAGTAGGGAAAGAGGTAAAAATGGTGAAAATGAGC

                                          840        850        860        870        880        890        900        910        920        930        940        950
                                          CAAAATATCAAAGAAACAAGAATAGATCAAGTAATCGCCCTCCTCAATCCAACCGCAATTTCAAAGTCGATATTCCGAATGAAGATTTTGACTTTCAATCAAATAATGCAAAATTCACG
U7551BL150-1.seq(1>3036)                    →  CAAAATATCAAAGAAACAAGAATAGATCAAGTAATCGCCCTCCTCAATCCAACCGCAATTTCAAAGTCGATATTCCGAATGAAGATTTTGACTTTCAATCAAATAATGCAAAATTCACG
B09-X82565-U7551BL150-1-1-1-SEQ1.ab1(1>783) →  CAAAATATCAAAGAAACAAGAATAGATCAAGTAATCGCCCTCCTCAATCCAACCGCAATTTCAAAGTCGATATTCCGAATGAAGATTTTGACTTTCAATCAAATAATGCAAAATTCACG

                                          960        970        980        990        1000       1010       1020       1030       1040       1050       1060       1070
                                          AAAGGTGATTCCACTGATGTGGAAAAAGAAAAAGAATTAGAATCAGCTGTTCACAAGCAGGATGAATCTGATGAGCAGTTTTATAATAAAAAATCGTCTTTTTTCGACACCATCTCCAC
U7551BL150-1.seq(1>3036)                    →  AAAGGTGATTCCACTGATGTGGAAAAAGAAAAAGAATTAGAATCAGCTGTTCACAAGCAGGATGAATCTGATGAGCAGTTTTATAATAAAAAATCGTCTTTTTTCGACACCATCTCCAC
B09-X82565-U7551BL150-1-1-1-SEQ1.ab1(1>783) →  AAAGGTGATTCCACTGATGTGGAAAAAGAAAAAGAATTAGAATCAGCTGTTCACAAGCAGGATGAATCTGATGAGCAGTTTTATAATAAAAAATCGTCTTTTTTCGACACCATCTCCAC
```

1080 1090 1100 1110 1120 1130 1140 1150 1160 1170 1180 1190

TTCTACTGAAACTAATACCAATATGAGATGGCAAGAAGAAAAAATGTTGAACGTTGACACCTTTGGACAAGCTTCTGCCAGACCAAGATTTCACTCTAGAGGCCTCGGTCGTGGGCGTG

U7551BL150-1.seq(1>3036) → TTCTACTGAAACTAATACCAATATGAGATGGCAAGAAGAAAAAATGTTGAACGTTGACACCTTTGGACAAGCTTCTGCCAGACCAAGATTTCACTCTAGAGGCCTCGGTCGTGGGCGTG
B09-X82565-U7551BL150-1-1-1-SEQ1.ab1(1>783) → TTCTACTGAAACTAATACCAATATGAGATGGCAAGAAGAAAAAATGTTGAACGTTGACACCTTTGGACAAGCTTCTGCCAGACCAAGATTTCACTCTAGAGGCCTCGGTCGTGGGCGTG

1200 1210 1220 1230 1240 1250 1260 1270 1280 1290 1300

GAAATTATAGGGGAAACAGAGGAAACAGAGGAAGAGGCGGCCAACGTGGAAACTACCAAAACAGAAATAACTACCAAAATGATAGTGGCGCCTATCAGAACCAAAACGACTCGTACAGC

U7551BL150-1.seq(1>3036) → GAAATTATAGGGGAAACAGAGGAAACAGAGGAAGAGGCGGCCAACGTGGAAACTACCAAAACAGAAATAACTACCAAAATGATAGTGGCGCCTATCAGAACCAAAACGACTCGTACAGC
B09-X82565-U7551BL150-1-1-1-SEQ1.ab1(1>783) → GAAATTATAGGGGAAACAGAGGAAACAGAGGAAGAGGCGGCCAACGTGGAAACTACCAAAACAGAAATAACTACCAAAATGATAGTGGCGCCTATCAGAACCAAAACGACTCGTACAGC
B10-X82565-U7551BL150-1-1-1-SEQ2.ab1(1>824) → CGTGGAaACTACCAAAACAGAAATAACTACCAAAATGATAGTGGCGCCTATCAGAACCAAAACGACTCGTACAGC

1310 1320 1330 1340 1350 1360 1370 1380 1390 1400 1410 1420

AGACCAGCCAACCAGTTTTCGCAACCTCCTTCCAACGTTGAATTTCTCGAGGGTGGTGGTGGTGGTTCTGCTTCTAACTTTACTCAGTTCGTTCTCGTCGACAATGGCGGAACTGGCGA

U7551BL150-1.seq(1>3036) → AGACCAGCCAACCAGTTTTCGCAACCTCCTTCCAACGTTGAATTTCTCGAGGGTGGTGGTGGTGGTTCTGCTTCTAACTTTACTCAGTTCGTTCTCGTCGACAATGGCGGAACTGGCGA
B09-X82565-U7551BL150-1-1-1-SEQ1.ab1(1>783) → AGACCAGCCAACCAGTTTTCGCAACCTCCTTCCAACGTTGAATTTCTCGAGGGTGGTGGTGGTGGTTCTGCTTCTAACTTTACTCAGTTCGTTCTCGTCG
B10-X82565-U7551BL150-1-1-1-SEQ2.ab1(1>824) → AGACCAGCCAACCAGTTTTCGCAACCTCCTTCCAACGTTGAATTTCTCGAGGGTGGTGGTGGTGGTTCTGCTTCTAACTTTACTCAGTTCGTTCTCGTCGACAATGGCGGAACTGGCGA

Linker MS2

1430 1440 1450 1460 1470 1480 1490 1500 1510 1520 1530 1540

CGTGACTGTCGCCCCAAGCAACTTCGCTAACGGGGTCGCTGAATGGATCAGCTCTAACTCGCGATCACAGGCTTACAAAGTAACCTGTAGCGTTCGTCAGAGCTCTGCGCAGAATCGCA

U7551BL150-1.seq(1>3036) → CGTGACTGTCGCCCCAAGCAACTTCGCTAACGGGGTCGCTGAATGGATCAGCTCTAACTCGCGATCACAGGCTTACAAAGTAACCTGTAGCGTTCGTCAGAGCTCTGCGCAGAATCGCA
B10-X82565-U7551BL150-1-1-1-SEQ2.ab1(1>824) → CGTGACTGTCGCCCCAAGCAACTTCGCTAACGGGGTCGCTGAATGGATCAGCTCTAACTCGCGATCACAGGCTTACAAAGTAACCTGTAGCGTTCGTCAGAGCTCTGCGCAGAATCGCA

1550 1560 1570 1580 1590 1600 1610 1620 1630 1640 1650 1660

AATACACCATCAAAGTCGAGGTGCCTAAAGTGGCAACCCAGACTGTTGGTGGTGAAGAGCTTCCTGTAGCCGGATGGAGATCTTACTTAAATATGGAACTAACCATTCCAATTTTCGCC

U7551BL150-1.seq(1>3036) → AATACACCATCAAAGTCGAGGTGCCTAAAGTGGCAACCCAGACTGTTGGTGGTGAAGAGCTTCCTGTAGCCGGATGGAGATCTTACTTAAATATGGAACTAACCATTCCAATTTTCGCC
B10-X82565-U7551BL150-1-1-1-SEQ2.ab1(1>824) → AATACACCATCAAAGTCGAGGTGCCTAAAGTGGCAACCCAGACTGTTGGTGGTGAAGAGCTTCCTGTAGCCGGATGGAGATCTTACTTAAATATGGAACTAACCATTCCAATTTTCGCC

1670 1680 1690 1700 1710 1720 1730 1740 1750 1760 1770 1780

ACGAATTCCGACTGCGAGCTTATTGTTAAGGCAATGCAAGGTCTCCTAAAAGATGGAAACCCGATTCCCTCGGCCATCGCAGCAAACTCCGGCATCTACGACTACAAAGACCATGACGG

U7551BL150-1.seq(1>3036) → ACGAATTCCGACTGCGAGCTTATTGTTAAGGCAATGCAAGGTCTCCTAAAAGATGGAAACCCGATTCCCTCGGCCATCGCAGCAAACTCCGGCATCTACGACTACAAAGACCATGACGG
B10-X82565-U7551BL150-1-1-1-SEQ2.ab1(1>824) → ACGAATTCCGACTGCGAGCTTATTGTTAAGGCAATGCAAGGTCTCCTAAAAGATGGAAACCCGATTCCCTCGGCCATCGCAGCAAACTCCGGCATCTACGACTACAAAGACCATGACGG

1790 1800 1810 1820 1830 1840 1850 1860 1870 1880 1890 1900

TGATTATAAAGATCATGACATCGACTACAAGGATGACGATGACAAGTAAAATGATGTTTCTATGTAAATTAAGTATATCATTCAGTATGTACATACAAAAATCAAATTAATAGTATTAT

U7551BL150-1.seq(1>3036) → TGATTATAAAGATCATGACATCGACTACAAGGATGACGATGACAAGTAAAATGATGTTTCTATGTAAATTAAGTATATCATTCAGTATGTACATACAAAAATCAAATTAATAGTATTAT
B10-X82565-U7551BL150-1-1-1-SEQ2.ab1(1>824) → TGATTATAAAGATCATGACATCGACTACAAGGATGACGATGACAAGTAAAATGATGTTTCTATGTAAATTAAGTATATCATTCAGTATGTACATACAAAAATCAAATTAATAGTATTAT
B11-X82565-U7551BL150-1-1-1-SEQ3.ab1(1>802) → ATCATTCAGTATGTACATACAAAAATCAAATTAATAGTATTAT

STOP SCD6 3'UTR

1910 1920 1930 1940 1950 1960 1970 1980 1990 2000 2010 2020

AGTCCGCGCCTCTGGATAGTATTATTTTAGTGCGTATCGCAAAATATGAATAGCACAGAGGTTCATTATTATGTTCTGAGTATGAGGACGAGGTAATACATACCTTAAAATCTTACATC

U7551BL150-1.seq(1>3036) → AGTCCGCGCCTCTGGATAGTATTATTTTAGTGCGTATCGCAAAATATGAATAGCACAGAGGTTCATTATTATGTTCTGAGTATGAGGACGAGGTAATACATACCTTAAAATCTTACATC
B10-X82565-U7551BL150-1-1-1-SEQ2.ab1(1>824) → AGTCCGCGCCTCTGGATAGTATTATTTTAGTGCGTATCGCAAAATATGAATAGCACAGAGGTTCATTATTATGTTCTGAGTATGAGGACGAGGTAATACATACCTTAAAATCTTACATC
B11-X82565-U7551BL150-1-1-1-SEQ3.ab1(1>802) → AGTCCGCGCCTCTGGATAGTATTATTTTAGTGCGTATCGCAAAATATGAATAGCACAGAGGTTCATTATTATGTTCTGAGTATGAGGACGAGGTAATACATACCTTAAAATCTTACATC

```
                                                   2030      2040      2050      2060      2070      2080      2090      2100      2110      2120      2130      2140
                                                   ATGTGGATAGCATTTATGGCTTCTTCCATCCGGCCGTACGCTTCTGTTCCTGTCCCTAGCCATAGCTTTTCTCATATCAAAAGCATCTTTATTGTCATCTACTTTTTTCAGGGTATCTG
U7551BL150-1.seq(1>3036)                       →   ATGTGGATAGCATTTATGGCTTCTTCCATCCGGCCGTACGCTTCTGTTCCTGTCCCTAGCCATAGCTTTTCTCATATCAAAAGCATCTTTATTGTCATCTACTTTTTTCAGGGTATCTG
B10-X82565-U7551BL150-1-1-1-SEQ2.ab1(1>824)    →   ATGTGGATAGCATTTATGGCTTCTTCCATCCGGCC
B11-X82565-U7551BL150-1-1-1-SEQ3.ab1(1>802)    →   ATGTGGATAGCATTTATGGCTTCTTCCATCCGGCCGTACGCTTCTGTTCCTGTCCCTAGCCATAGCTTTTCTCATATCAAAAGCATCTTTATTGTCATCTACTTTTTTCAGGGTATCTG

                                                   2150      2160      2170      2180      2190      2200      2210      2220      2230      2240      2250      2260
                                                   GATACCCATCTTCAGCTGAGTTATAATATCCCACCACTCTCCTGTAGACACTGTATCCCAATTTTTCGTAAAGTTTGATGGCCAATTGGTTATTGCACTTGACGAAAAGATCGATAAAG
U7551BL150-1.seq(1>3036)                       →   GATACCCATCTTCAGCTGAGTTATAATATCCCACCACTCTCCTGTAGACACTGTATCCCAATTTTTCGTAAAGTTTGATGGCCAATTGGTTATTGCACTTGACGAAAAGATCGATAAAG
B11-X82565-U7551BL150-1-1-1-SEQ3.ab1(1>802)    →   GATACCCATCTTCAGCTGAGTTATAATATCCCACCACTCTCCTGTAGACACTGTATCCCAATTTTTCGTAAAGTTTGATGGCCAATTGGTTATTGCACTTGACGAAAAGATCGATAAAG

                                                   2270      2280      2290      2300      2310      2320      2330      2340      2350      2360      2370      2380
                                                   TTGACTTCATGGGGCATGACATCGGTCATGGTCTCCAAAGTATTACATAACTTCGAAGCTAGCGATATTCGGCGGAATCTTGGAGCTACTGTCACTGCAGTTATATGAGTGTGCCACTC
U7551BL150-1.seq(1>3036)                       →   TTGACTTCATGGGGCATGACATCGGTCATGGTCTCCAAAGTATTACATAACTTCGAAGCTAGCGATATTCGGCGGAATCTTGGAGCTACTGTCACTGCAGTTATATGAGTGTGCCACTC
B11-X82565-U7551BL150-1-1-1-SEQ3.ab1(1>802)    →   TTGACTTCATGGGGCATGACATCGGTCATGGTCTCCAAAGTATTACATAACTTCGAAGCTAGCGATATTCGGCGGAATCTTGGAGCTACTGTCACTGCAGTTATATGAGTGTGCCACTC
B08-X82565-U7551BL150-1-M13F-77.ab1(1>790)     ←                                                                         TAGCGATATTCGGCGGAATCTTGGAGCTACTGTCACTGCAGTTATATGAGTGTGCCACTC

                                                   2390      2400      2410      2420      2430      2440      2450      2460      2470      2480      2490
                                                   CGTGGTCTTGCCTTCTGTTTTTGCCATCATGTAACCAGAGATATTATGCTTAAATGTAGGATCAACAGTCATTTCTGAACTTTTGAAAAAAAGGTCTGGCCATATTATCATGTATTCAA
U7551BL150-1.seq(1>3036)                       →   CGTGGTCTTGCCTTCTGTTTTTGCCATCATGTAACCAGAGATATTATGCTTAAATGTAGGATCAACAGTCATTTCTGAACTTTTGAAAAAAAGGTCTGGCCATATTATCATGTATTCAA
B11-X82565-U7551BL150-1-1-1-SEQ3.ab1(1>802)    →   CGTGGTCTTGCCTTCTGTTTTTGCCATCATGTAACCAGAGATATTATGCTTAAATGTAGGATCAACAGTCATTTCTGAACTTTTGAAAAAAAGGTCTGGCCATATTATCATGTATTCAA
B08-X82565-U7551BL150-1-M13F-77.ab1(1>790)     ←   CGTGGTCTTGCCTTCTGTTTTTGCCATCATGTAACCAGAGATATTATGCTTAAATGTAGGATCAACAGTCATTTCTGAACTTTTGAAAAAAAGGTCTGGCCATATTATCATGTATTCAA

                                                 2500      2510      2520      2530      2540      2550      2560      2570      2580      2590      2600      2610
                                                   AATAAAACTCTAAAGGGAAGTTTTCTGTTAAGATATCAAGGTTGACATTGTTAGTTTTGAATAAATCGACAGGTTCAAAAGGCTGAATTGTTGTCATTTCTCGAATCCTCCTGTCTCTT
U7551BL150-1.seq(1>3036)                       →   AATAAAACTCTAAAGGGAAGTTTTCTGTTAAGATATCAAGGTTGACATTGTTAGTTTTGAATAAATCGACAGGTTCAAAAGGCTGAATTGTTGTCATTTCTCGAATCCTCCTGTCTCTT
B11-X82565-U7551BL150-1-1-1-SEQ3.ab1(1>802)    →   AATAAAACTCTAAAGGGAAGTTTTCTGTTAAGATATCAAGGTTGACATTGTTAGTTTTGAATAAATCGACAGGTTCAAAAGGCTGAATTGTTGTCATTTCTCGAATCCTCCTGTCTCTT
B08-X82565-U7551BL150-1-M13F-77.ab1(1>790)     ←   AATAAAACTCTAAAGGGAAGTTTTCTGTTAAGATATCAAGGTTGACATTGTTAGTTTTGAATAAATCGACAGGTTCAAAAGGCTGAATTGTTGTCATTTCTCGAATCCTCCTGTCTCTT

                                                  2620      2630      2640      2650      2660      2670      2680      2690      2700      2710      2720      2730
                                                   TCCTTGAATATTCTCAATGTTCCACTATCTTTCACATAAGCAATATTGTGAACTATATATACTTCATTAGATATTGCTTCAACATTAATATTAATAAAAAGGACATGCATAAAGTAACA
U7551BL150-1.seq(1>3036)                       →   TCCTTGAATATTCTCAATGTTCCACTATCTTTCACATAAGCAATATTGTGAACTATATATACTTCATTAGATATTGCTTCAACATTAATATTAATAAAAAGGACATGCATAAAGTAACA
B11-X82565-U7551BL150-1-1-1-SEQ3.ab1(1>802)    →   TCCTTGAATATTCTCAATGTTCCACTATCTTTCACATAAGCAATA
B08-X82565-U7551BL150-1-M13F-77.ab1(1>790)     ←   TCCTTGAATATTCTCAATGTTCCACTATCTTTCACATAAGCAATATTGTGAACTATATATACTTCATTAGATATTGCTTCAACATTAATATTAATAAAAAGGACATGCATAAAGTAACA

                                                   2740      2750      2760      2770      2780      2790      2800      2810      2820      2830      2840      2850
                                                   TCCGAACATCTCCAAACACCCATACATATTTATATTATCGACGAGGGAATTTTTTTCTTCCCAGGCCCACCAACGTGCGCCCACCGCTATGTTTTTTGCGCCTAGGTAGGTGGGCTAGC
U7551BL150-1.seq(1>3036)                       →   TCCGAACATCTCCAAACACCCATACATATTTATATTATCGACGAGGGAATTTTTTTCTTCCCAGGCCCACCAACGTGCGCCCACCGCTATGTTTTTTGCGCCTAGGTAGGTGGGCTAGC
B08-X82565-U7551BL150-1-M13F-77.ab1(1>790)     ←   TCCGAACATCTCCAAACACCCATACATATTTATATTATCGACGAGGGAATTTTTTTCTTCCCAGGCCCACCAACGTGCGCCCACCGCTATGTTTTTTGCGCCTAGGTAGGTGGGCTAGC

                                                   2860      2870      2880      2890      2900      2910      2920      2930      2940      2950      2960      2970
                                                   TTCTATGTATCAAAGCTCCTTCTGCGCAGCCGAGGTTTTGCTAAGCACTACCGCATTGGAATGACTGTGTTTGTAGGAGCTCCTTGTCTCCACGCTTTCGCAATTTTCAAGATCGAGAT
U7551BL150-1.seq(1>3036)                       →   TTCTATGTATCAAAGCTCCTTCTGCGCAGCCGAGGTTTTGCTAAGCACTACCGCATTGGAATGACTGTGTTTGTAGGAGCTCCTTGTCTCCACGCTTTCGCAATTTTCAAGATCGAGAT
B08-X82565-U7551BL150-1-M13F-77.ab1(1>790)     ←   TTCTATGTATCAAAGCTCCTTCTGCGCAGCCGAGGTTTTGCTAAGCACTACCGCATTGGAATGACTGTGTTTGTAGGAGCTCCTTGTCTCCACGCTTTCGCAATTTTCAAGATCGAGAT
```

```
                                                       2980      2990      3000      3010      3020      3030      3040      3050      3060      3070      3080      3090
                                                       GCTATTAGTGATGGTAAGGTTATGAAGCTTACATGTTTAATATTGTAGATTCCAGAAGCAGGTAAGAACAACAAACTACAAGAAAAGGGTACCCGGGTACCGAGCTCGAATTCACTGGC
U7551BL150-1.seq(1>3036)                          →    GCTATTAGTGATGGTAAGGTTATGAAGCTTACATGTTTAATATTGTAGATTCCAGAAGCAGGTAAGAACAACAAACTACAAGAAAAGGGTACCCGGG
B08-X82565-U7551BL150-1-M13F-77.ab1(1>790)        ←    GCTATTAGTGATGGTAAGGTTATGAAGCTTACATGTTTAATATTGTAGATTCCAGAAGCAGGTAAGAACAACAAACTACAAGAAAAGGGTACCCGGGTACCGAGCTCGAATTCACTGGC
                                                                                                                                        SmaI

                                                         3100      3110
                                                       CGTCGTTtTACAACGT
B08-X82565-U7551BL150-1-M13F-77.ab1(1>790)        ←    CGTCGTTtTACAACGT
```

Certificate of Analysis

Project ID: U7551BL150-1

Construct Information:

Gene Name: DeltaLsm-Scd6-MS2

Clone ID: X82565

Gene Length: 3035 bp

Cloning Vector: YCpLac111

Cloning Strategy: SphI / SmaI

QC Items	Specifications	Results	
**Sequencing Alignment**	Sequencing results are consistent with the targeted insert sequence.	Pass	Consistent
**Vector Sequence**	The flanking sequences of the cloning site are correct.	Pass	Correct<br>Shown in the SQD file
**Restriction Digests**	The size of inserted fragment is correct and free of unexpected bands suggesting contamination.	Pass	Correct<br>Shown in attachment 1
**DNA Quality**	Miniprep: 4 µg<br>OD260/280=1.8~2.0<br>Free of contamination	Pass	⩾ 4 µg<br>OD260/280=1.81<br>Pure
**Quality grade**	Research Grade	Pass	Research Grade
**Appearance**	Clear and free of foreign particles.	Pass	Clear<br>Free of foreign particles
**Additional Test**		N/A	

NOTE

Shipping at	Plasmid Storing at	Bacstab Storing at	Glycerol Stock Storing at
Room Temperature	-20℃	4℃	-20℃/-80℃

Morgan

Certified by: **Date: 02/08/2017**

Thank you for your patronage to our Gene services! To maintain this working relationship, we shall be grateful if you can add our webpage URL into your lab website. As a token of appreciation, you will be rewarded by 1,000 EZcoupon™ points. For more information, please contact us by e-mail at web@genscript.com.

For research use only

860 Centennial Ave., Piscataway, NJ 08854, USA
Toll-Free: 1-877-436-7274 Tel: 1-732-885-9188 Fax: 1-732-210-0262 Email: order@genscript.com Web: www.genscript.com

Attachment 1

Plasmid Construct Map

The gene was cloned in YCpLac111 by SphI / SmaI.

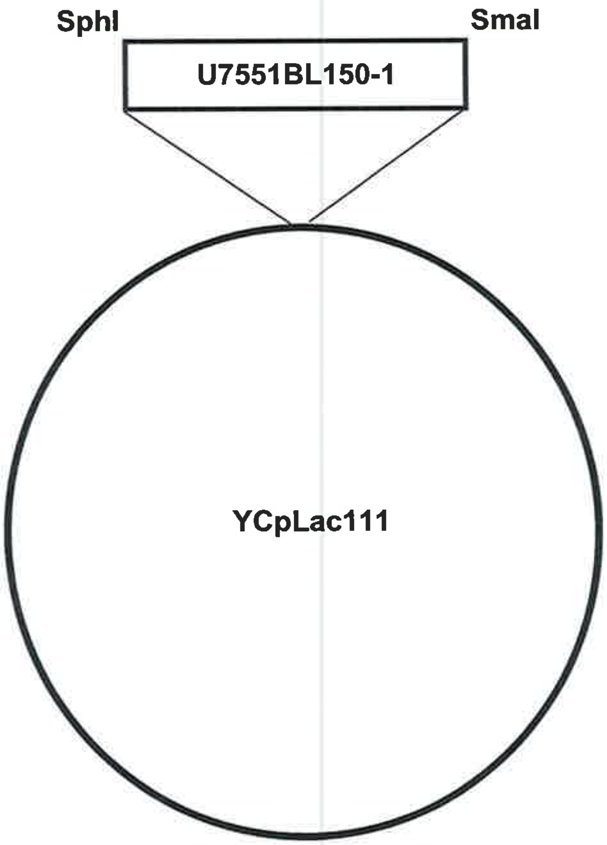

Enzyme Digestion

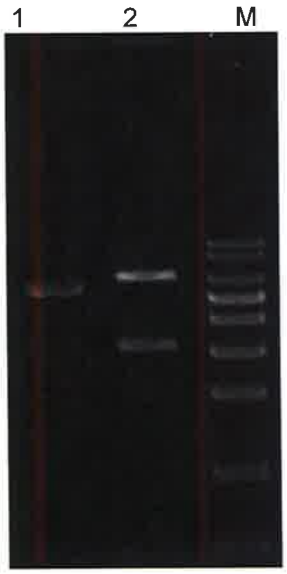

Lane M: KB Ladder
Lane 1:U7551BL150-1 plasmid
Lane 2:U7551BL150-1 plasmid digested by SphI and SmaI

Digestion Conditions:
About 300ng plasmid digested
Digestion in water-bath,37℃ for 40 minutes
1% Agarose Gel

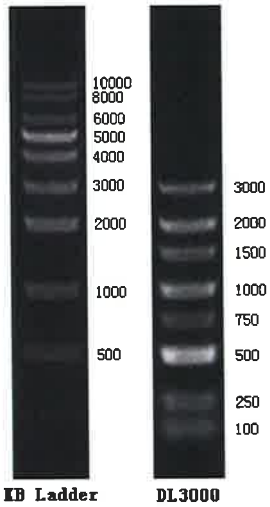

For research use only

ΔRGG-SCD6-MS2

Description: YCplac111 - 474 bp upstream – cds (minus 96 bp near the C-terminus) - linker – MS2CP (minus ATG minus stop) – 3xFlag – stop (TAA) – 3'UTR Scd6 (1232 bp) – Ycplac111

Protein sequence alteration: deletion from S287 to N318 (32 aa) corresponding to RGG/X motifs (see Scd6 multiple sequence alignment).

DNA Sequence

GCATGCCTGCAGGTCGACTCTAGTTGCTCGTAACAATCTTGGCCTAGCTTCCAGGGTGTCTGTAC
TTTTACGCAGCATCATATTTTCTCTATTTTTTGCACATCTTCTTGCTCTTCTTATATTTACCAATTAT
CTGAAATAGTCACTCGGACCGAGATGCAAAAATCGGATTTAAAACTCGATCCGAGGAGAACAC
CAAATGACACAACTTCTATCAATGCAATTGCCCTCTAAGTATCATAGATGCCGCTATTTTAAATG
ACAGTGCAATCGATTTCCATATTAAATGGATAAAGGTGTTTATTATGAAGATACCCATTACTGAC
GTATATGTAATGATTCCTCAGTCCAGTACCATAGTCCCGTCACCTTACACGGTGGAAAAATTAAA
CTGTACCCGCGATTGTAAAGAATAACGTGGAGGCAGTCAGAAAGAACAATAAACGAATAGTGA
GGATGTCGTAGAAGAAGCATTCATCGAAAAACAGCAGCAAGGCAATGTCGCAGTACATCGGTA
AAACTATTTCTTTAATCTCTGTGACTGACAACAGATATGTGGGGCTGTTAGAAGATATTGACTCT
GAAAAGGGTACCGTGACTTTGAAAGAAGTTCGCTGTTTTGGTACAGAAGGTCGCAAGAACTGG
GGTCCTGAAGAAATTTATCCGAATCCTACGGTATACAATTCTGTAAAGTTCAACGGCAGTGAAG
TCAAGGATTTAAGCATTTTAGATGCTAACATCAATGACATACAGCCGGTTGTTCCTCAAATGATG
CCACCCGCTTCACAATTCCCTCCTCAACAAGCTCAATCTCCACCCCAGGCTCAAGCTCAAGCACA
CGTGCAAACAAACCCCCAAGTTCCAAAGCCCGAATCCAATGTGCCAGCAGCTGTCGCTGGATAT
GGTGTTTACACCCCAACTTCGACAGAAACCGCTACTGCTAGTATGAATGATAAGAGCACTCCTC
AAGACACCAATGTAAACTCGCAAAGTAGGGAAAGAGGTAAAAATGGTGAAAATGAGCCAAAA
TATCAAAGAAACAAGAATAGATCAAGTAATCGCCCTCCTCAATCCAACCGCAATTTCAAAGTCG
ATATTCCGAATGAAGATTTTGACTTTCAATCAAATAATGCAAAATTCACGAAAGGTGATTCCACT
GATGTGGAAAAAGAAAAAGAATTAGAATCAGCTGTTCACAAGCAGGATGAATCTGATGAGCAG
TTTTATAATAAAAAATCGTCTTTTTTCGACACCATCTCCACTTCTACTGAAACTAATACCAATATG
AGATGGCAAGAAGAAAAAATGTTGAACGTTGACACCTTTGGACAAGCTTCTGCCAGACCAAGA
TTTCACTACCAAAATGATAGTGGCGCCTATCAGAACCAAAACGACTCGTACAGCAGACCAGCCA
ACCAGTTTTCGCAACCTCCTTCCAACGTTGAATTTCTCGAGGGTGGTGGTGGTGGTTCTGCTTCT
AACTTTACTCAGTTCGTTCTCGTCGACAATGGCGGAACTGGCGACGTGACTGTCGCCCCAAGCA
ACTTCGCTAACGGGGTCGCTGAATGGATCAGCTCTAACTCGCGATCACAGGCTTACAAAGTAAC
CTGTAGCGTTCGTCAGAGCTCTGCGCAGAATCGCAAATACACCATCAAAGTCGAGGTGCCTAAA
GTGGCAACCCAGACTGTTGGTGGTGAAGAGCTTCCTGTAGCCGGATGGAGATCTTACTTAAATA
TGGAACTAACCATTCCAATTTTCGCCACGAATTCCGACTGCGAGCTTATTGTTAAGGCAATGCAA
GGTCTCCTAAAAGATGGAAACCCGATTCCCTCGGCCATCGCAGCAAACTCCGGCATCTACGACT
ACAAAGACCATGACGGTGATTATAAAGATCATGACATCGACTACAAGGATGACGATGACAAGT
AAAATGATGTTTCTATGTAAATTAAGTATATCATTCAGTATGTACATACAAAAATCAAATTAATA
GTATTATAGTCCGCGCCTCTGGATAGTATTATTTTAGTGCGTATCGCAAAATATGAATAGCACAG
AGGTTCATTATTATGTTCTGAGTATGAGGACGAGGTAATACATACCTTAAAATCTTACATCATGT
GGATAGCATTTATGGCTTCTTCCATCCGGCCGTACGCTTCTGTTCCTGTCCCTAGCCATAGCTTTT

CTCATATCAAAAGCATCTTTATTGTCATCTACTTTTTTCAGGGTATCTGGATACCCATCTTCAGCT
GAGTTATAATATCCCACCACTCTCCTGTAGACACTGTATCCCAATTTTTCGTAAAGTTTGATGGCC
AATTGGTTATTGCACTTGACGAAAAGATCGATAAAGTTGACTTCATGGGGCATGACATCGGTCA
TGGTCTCCAAAGTATTACATAACTTCGAAGCTAGCGATATTCGGCGGAATCTTGGAGCTACTGT
CACTGCAGTTATATGAGTGTGCCACTCCGTGGTCTTGCCTTCTGTTTTTGCCATCATGTAACCAG
AGATATTATGCTTAAATGTAGGATCAACAGTCATTTCTGAACTTTTGAAAAAAAGGTCTGGCCAT
ATTATCATGTATTCAAAATAAAACTCTAAAGGGAAGTTTTCTGTTAAGATATCAAGGTTGACATT
GTTAGTTTTGAATAAATCGACAGGTTCAAAAGGCTGAATTGTTGTCATTTCTCGAATCCTCCTGT
CTCTTTCCTTGAATATTCTCAATGTTCCACTATCTTTCACATAAGCAATATTGTGAACTATATATAC
TTCATTAGATATTGCTTCAACATTAATATTAATAAAAAGGACATGCATAAAGTAACATCCGAACA
TCTCCAAACACCCATACATATTTATATTATCGACGAGGGAATTTTTTTCTTCCCAGGCCCACCAAC
GTGCGCCCACCGCTATGTTTTTTGCGCCTAGGTAGGTGGGCTAGCTTCTATGTATCAAAGCTCCT
TCTGCGCAGCCGAGGTTTTGCTAAGCACTACCGCATTGGAATGACTGTGTTTGTAGGAGCTCCT
TGTCTCCACGCTTTCGCAATTTTCAAGATCGAGATACTATTAGTGATGGTAAGGTTATGAAGCTT
ACATGTTTAATATTGTAGATTCCAGAAGCAGGTAAGAACAACAAACTACAAGAAAAGGGTACCC
GGGTACCGAGCTCGAATTCACTGGCCGTCGTTTTACAACGTCGTGACTGGGAAAACCCTGGCGT
GAA

Legend

1. GCATGC = SphI restriction site
2. CTGCAGGTCGACTCTAGT = vector DNA
3. TGCTCG ……… AAGGCA = SCD6 native promoter (474 bp)
4. ATGTCGCAG ……. GAATTTCTCGAG = Scd6 cds with 96 bp RGG deletion minus stop codon (957 bp); start codon highlighted yellow
5. GGTGGTGGTGGTGGTTCT = linker
6. GCTTCTAACTTTACTCAGTTCGTTCTCGTCGACAATGGCGGAACTGGCGACGTGACTGT
CGCCCCAAGCAACTTCGCTAACGGGGTCGCTGAATGGATCAGCTCTAACTCGCGATCAC
AGGCTTACAAAGTAACCTGTAGCGTTCGTCAGAGCTCTGCGCAGAATCGCAAATACACC
ATCAAAGTCGAGGTGCCTAAAGTGGCAACCCAGACTGTTGGTGGTGAAGAGCTTCCTG
TAGCCGGATGGAGATCTTACTTAAATATGGAACTAACCATTCCAATTTTCGCCACGAAT
TCCGACTGCGAGCTTATTGTTAAGGCAATGCAAGGTCTCCTAAAAGATGGAAACCCGA
TTCCCTCGGCCATCGCAGCAAACTCCGGCATCTAC = MS2 (387 bp)
7. GACTACAAAGACCATGACGGTGATTATAAAGATCATGACATCGACTACAAGGATGACG
ATGACAAGTAA = 3xFlag tag + Stop codon
8. AATGAT ….. AAGGGTA = Scd6 3’ UTR (1232 bp)
9. CCCGGG = SmaI restriction site

10. TACCGAGCTCGAATTCACTGGCCGTCGTTTTACAACGTCGTGACTGGGAAAACCCTGGC GTGAA = vector DNA

Deleted fragment corresponding to S287-N318 (in original WT sequence; missing from construct above)
TCTAGAGGCCTCGGTCGTGGGCGTGGAAATTATAGGGGAAACAGAGGAAACAGAGGAAGAGG CGGCCAACGTGGAAACTACCAAAACAGAAATAAC

From ATG to beginning of RGG box = 858 bp = 286 aa

C-terminus = aa Y319 to aa E349

Packing List

GenScript
The Biology CRO

GenScript USA Inc.
860 Centennial Ave, Piscataway, NJ 08854, USA
Tel:732-885-9188 Fax:732-210-0262
E-mail: order@genscript.com

Ship To: Quira Zeidan
National Institutes of Health (NIH) - MD
Bldg 6, BETHMC, 6 Center Drive
Bethesda, MD 20892, US
3014356962

Bill To: Quira Zeidan
National Institutes of Health (NIH) - MD
2115 E Jefferson St., MSC 8500 Suite 4B 432
Bethesda, MD 20892, US
3014356962

Customer No.	PO#	Order#	Order Date	Ship Via	Shipping Date	Terms	Total Weight	# Of Packages
**204455**	4420227	U7551BL150	2016-12-16	FedEx Domestic Standard Overnight		Net 30	0.5 (lb)	1/1

Item	Catalog number	Order Qty	Ship Qty	Description
**U7551BL150_2**	SC1023	1	1	DeltaRGG-Scd6-MS2: Target Insert Name: DeltaRGG-Scd6-MS2, Template informatio n: Insert name: Scd6-MS2, Cloning site: SphI-SmaI, Vector name: YCpLac111, Vector size(bp): 6112, Resistance: Ampicillin, Copy number: Low, Target vector: Same as te mplate,<br>Plasmid preparation: Standard delivery: 4 µg (Free of charge)

ALL REAGENTS ARE FOR RESEARCH USE ONLY AND ARE NOT INTENDED FOR DIAGNOSTIC OR THERAPEUTIC USE. GENSCRIPT MAKES NO CLAIMS TO THE PRODUCT'S ABILITY TO FUNCTION IN THE SPECIFIC APPLICATION OF THE CUSTOMER.

This shipment completes your order #U7551BL150

34

10 20 30 40 50 60 70 80 90 100 110 120

GGAaACAGCTATGACCATGATTACGCCAAGCTTGCATGCCTGCAGGTCGACTCTAGTTGCTCGTAACAATCTTGGCCTAGCTTCCAGGGTGTCTGTACTTTTACGCAGCATCATATTTTCTCTATTT

B07-X83417-U7551BL150-2-M13R-88.ab1(1>632) → GGAaACAGCTATGACCATGATTACGCCAAGCTTGCATGCCTGCAGGTCGACTCTAGTTGCTCGTAACAATCTTGGCCTAGCTTCCAGGGTGTCTGTACTTTTACGCAGCATCATATTTTCTCTATTT
U7551BL150-2.seq(1>3168) → GCATGCCTGCAGGTCGACTCTAGTTGCTCGTAACAATCTTGGCCTAGCTTCCAGGGTGTCTGTACTTTTACGCAGCATCATATTTTCTCTATTT

SphI SCD6 promoter

130 140 150 160 170 180 190 200 210 220 230 240 250

TTTGCACATCTTCTTGCTCTTCTTATATTTACCAATTATCTGAAATAGTCACTCGGACCGAGATGCAAAAATCGGATTTAAAACTCGATCCGAGGAGAACACCAAATGACACAACTTCTATCAATGC

B07-X83417-U7551BL150-2-M13R-88.ab1(1>632) → TTTGCACATCTTCTTGCTCTTCTTATATTTACCAATTATCTGAAATAGTCACTCGGACCGAGATGCAAAAATCGGATTTAAAACTCGATCCGAGGAGAACACCAAATGACACAACTTCTATCAATGC
U7551BL150-2.seq(1>3168) → TTTGCACATCTTCTTGCTCTTCTTATATTTACCAATTATCTGAAATAGTCACTCGGACCGAGATGCAAAAATCGGATTTAAAACTCGATCCGAGGAGAACACCAAATGACACAACTTCTATCAATGC
H08-X83417-U7551BL150-2-1-2-SEQ1R.ab1(1>737) ← TATTTACCAATTATCTGAAATAGTCACTCGGACCGAGATGCAAAAATCGGATTTAAAACTCGATCCGAGGAGAACACCAAATGACACAACTTCTATCAATGC

260 270 280 290 300 310 320 330 340 350 360 370 380

AATTGCCCTCTAAGTATCATAGATGCCGCTATTTTAAATGACAGTGCAATCGATTTCCATATTAAATGGATAAAGGTGTTTATTATGAAGATACCCATTACTGACGTATATGTAATGATTCCTCAGT

B07-X83417-U7551BL150-2-M13R-88.ab1(1>632) → AATTGCCCTCTAAGTATCATAGATGCCGCTATTTTAAATGACAGTGCAATCGATTTCCATATTAAATGGATAAAGGTGTTTATTATGAAGATACCCATTACTGACGTATATGTAATGATTCCTCAGT
U7551BL150-2.seq(1>3168) → AATTGCCCTCTAAGTATCATAGATGCCGCTATTTTAAATGACAGTGCAATCGATTTCCATATTAAATGGATAAAGGTGTTTATTATGAAGATACCCATTACTGACGTATATGTAATGATTCCTCAGT
H08-X83417-U7551BL150-2-1-2-SEQ1R.ab1(1>737) ← AATTGCCCTCTAAGTATCATAGATGCCGCTATTTTAAATGACAGTGCAATCGATTTCCATATTAAATGGATAAAGGTGTTTATTATGAAGATACCCATTACTGACGTATATGTAATGATTCCTCAGT

390 400 410 420 430 440 450 460 470 480 490 500

CCAGTACCATAGTCCCGTCACCTTACACGGTGGAAAAATTAAACTGTACCCGCGATTGTAAAGAATAACGTGGAGGCAGTCAGAAAGAACAATAAACGAATAGTGAGGATGTCGTAGAAGAAGCATT

B07-X83417-U7551BL150-2-M13R-88.ab1(1>632) → CCAGTACCATAGTCCCGTCACCTTACACGGTGGAAAAATTAAACTGTACCCGCGATTGTAAAGAATAACGTGGAGGCAGTCAGAAAGAACAATAAACGAATAGTGAGGATGTCGTAGAAGAAGCATT
U7551BL150-2.seq(1>3168) → CCAGTACCATAGTCCCGTCACCTTACACGGTGGAAAAATTAAACTGTACCCGCGATTGTAAAGAATAACGTGGAGGCAGTCAGAAAGAACAATAAACGAATAGTGAGGATGTCGTAGAAGAAGCATT
H08-X83417-U7551BL150-2-1-2-SEQ1R.ab1(1>737) ← CCAGTACCATAGTCCCGTCACCTTACACGGTGGAAAAATTAAACTGTACCCGCGATTGTAAAGAATAACGTGGAGGCAGTCAGAAAGAACAATAAACGAATAGTGAGGATGTCGTAGAAGAAGCATT

532

510 520 530 540 550 560 570 580 590 600 610 620 630

CATCGAAAAACAGCAGCAAGGCAATGTCGCAGTACATCGGTAAAACTATTTCTTTAATCTCTGTGACTGACAACAGATATGTGGGGCTGTTAGAAGATATTGACTCTGAAAAGGGTACCGTGACTTT

B07-X83417-U7551BL150-2-M13R-88.ab1(1>632) → CATCGAAAAACAGCAGCAAGGCAATGTCGCAGTACATCGGTAAAACTATTTCTTTAATCTCTGTGACTGACAACAGATATGTGGGGCTGTTAGAAGATATTGACTCTGAAAAGGGTACCGTGAC
U7551BL150-2.seq(1>3168) → CATCGAAAAACAGCAGCAAGGCAATGTCGCAGTACATCGGTAAAACTATTTCTTTAATCTCTGTGACTGACAACAGATATGTGGGGCTGTTAGAAGATATTGACTCTGAAAAGGGTACCGTGACTTT
H08-X83417-U7551BL150-2-1-2-SEQ1R.ab1(1>737) ← CATCGAAAAACAGCAGCAAGGCAATGTCGCAGTACATCGGTAAAACTATTTCTTTAATCTCTGTGACTGACAACAGATATGTGGGGCTGTTAGAAGATATTGACTCTGAAAAGGGTACCGTGACTTT

SCD6 cds

640 650 660 670 680 690 700 710 720 730 740 750 760

GAAAGAAGTTCGCTGTTTTGGTACAGAAGGTCGCAAGAACTGGGGTCCTGAAGAAATTTATCCGAATCCTACGGTATACAATTCTGTAAAGTTCAACGGCAGTGAAGTCAAGGATTTAAGCATTTTA

U7551BL150-2.seq(1>3168) → GAAAGAAGTTCGCTGTTTTGGTACAGAAGGTCGCAAGAACTGGGGTCCTGAAGAAATTTATCCGAATCCTACGGTATACAATTCTGTAAAGTTCAACGGCAGTGAAGTCAAGGATTTAAGCATTTTA
H08-X83417-U7551BL150-2-1-2-SEQ1R.ab1(1>737) ← GAAAGAAGTTCGCTGTTTTGGTACAGAAGGTCGCAAGAACTGGGGTCCTGAAGAAATTTATCCGAATCCTACGGTATACAATTCTGTAAAGTTCAACGGCAGTGAAGTCAAGGATTTAAGCATTTTA

770 780 790 800 810 820 830 840 850 860 870 880

GATGCTAACATCAATGACATACAGCCGGTTGTTCCTCAAATGATGCCACCCGCTTCACAATTCCCTCCTCAACAAGCTCAATCTCCACCCCAGGCTCAAGCTCAAGCACACGTGCAAACAAACCCCC

U7551BL150-2.seq(1>3168) → GATGCTAACATCAATGACATACAGCCGGTTGTTCCTCAAATGATGCCACCCGCTTCACAATTCCCTCCTCAACAAGCTCAATCTCCACCCCAGGCTCAAGCTCAAGCACACGTGCAAACAAACCCCC
H08-X83417-U7551BL150-2-1-2-SEQ1R.ab1(1>737) ← GATGCTAACATCAATGACATACAGCCGGTTGTTCCTCAAATGATGCCACCCGCTTCACAATTCCCTCCTCAACAAGCTCAATCTCCACCCCAGGCTCAAGCTCAAGCACACGTGCAAACAAACCCCC
H09-X83417-U7551BL150-2-1-1-SEQ1.ab1(1>823) → GCTCAAGCACACGTGCAAACAAACCCCC

890 900 910 920 930 940 950 960 970 980 990 1000 1010

AAGTTCCAAAGCCCGAATCCAATGTGCCAGCAGCTGTCGCTGGATATGGTGTTTACACCCCAACTTCGACAGAAACCGCTACTGCTAGTATGAATGATAAGAGCACTCCTCAAGACACCAATGTAAA

U7551BL150-2.seq(1>3168) → AAGTTCCAAAGCCCGAATCCAATGTGCCAGCAGCTGTCGCTGGATATGGTGTTTACACCCCAACTTCGACAGAAACCGCTACTGCTAGTATGAATGATAAGAGCACTCCTCAAGACACCAATGTAAA
H09-X83417-U7551BL150-2-1-1-SEQ1.ab1(1>823) → AAGTTCCAAAGCCCGAATCCAATGTGCCAGCAGCTGTCGCTGGATATGGTGTTTACACCCCAACTTCGACAGAAACCGCTACTGCTAGTATGAATGATAAGAGCACTCCTCAAGACACCAATGTAAA

```
                                                   1020      1030      1040      1050      1060      1070      1080      1090      1100      1110      1120      1130      1140
                                                   CTCGCAAAGTAGGGAAAGAGGTAAAAATGGTGAAAATGAGCCAAAATATCAAAGAAACAAGAATAGATCAAGTAATCGCCCTCCTCAATCCAACCGCAATTTCAAAGTCGATATTCCGAATGAAGAT
U7551BL150-2.seq(1>3168)                      →    CTCGCAAAGTAGGGAAAGAGGTAAAAATGGTGAAAATGAGCCAAAATATCAAAGAAACAAGAATAGATCAAGTAATCGCCCTCCTCAATCCAACCGCAATTTCAAAGTCGATATTCCGAATGAAGAT
H09-X83417-U7551BL150-2-1-1-SEQ1.ab1(1>823)   →    CTCGCAAAGTAGGGAAAGAGGTAAAAATGGTGAAAATGAGCCAAAATATCAAAGAAACAAGAATAGATCAAGTAATCGCCCTCCTCAATCCAACCGCAATTTCAAAGTCGATATTCCGAATGAAGAT
```

```
                                                   1150      1160      1170      1180      1190      1200      1210      1220      1230      1240      1250      1260      1270
                                                   TTTGACTTTCAATCAAATAATGCAAAATTCACGAAAGGTGATTCCACTGATGTGGAAAAAGAAAAAGAATTAGAATCAGCTGTTCACAAGCAGGATGAATCTGATGAGCAGTTTTATAATAAAAAAT
U7551BL150-2.seq(1>3168)                      →    TTTGACTTTCAATCAAATAATGCAAAATTCACGAAAGGTGATTCCACTGATGTGGAAAAAGAAAAAGAATTAGAATCAGCTGTTCACAAGCAGGATGAATCTGATGAGCAGTTTTATAATAAAAAAT
H09-X83417-U7551BL150-2-1-1-SEQ1.ab1(1>823)   →    TTTGACTTTCAATCAAATAATGCAAAATTCACGAAAGGTGATTCCACTGATGTGGAAAAAGAAAAAGAATTAGAATCAGCTGTTCACAAGCAGGATGAATCTGATGAGCAGTTTTATAATAAAAAAT
G05-X83417-U7551BL150-2-1-2-seq2r.ab1(1>812)  ←                                                                                 GCTGTTCACAAGCAGGATGAATCTGATGAGCAGTTTTATAATAAAAAAT
```

```
                                                   1280      1290      1300      1310      1320      1330      1340      1350      1360      1370      1380      1390
                                                   CGTCTTTTTTCGACACCATCTCCACTTCTACTGAAACTAATACCAATATGAGATGGCAAGAAGAAAAAAATGTTGAACGTTGACACCTTTGGACAAGCTTCTGCCAGACCAAGATTTCACTACCAAAA
U7551BL150-2.seq(1>3168)                      →    CGTCTTTTTTCGACACCATCTCCACTTCTACTGAAACTAATACCAATATGAGATGGCAAGAAGAAAAAAATGTTGAACGTTGACACCTTTGGACAAGCTTCTGCCAGACCAAGATTTCACTACCAAAA
H09-X83417-U7551BL150-2-1-1-SEQ1.ab1(1>823)   →    CGTCTTTTTTCGACACCATCTCCACTTCTACTGAAACTAATACCAATATGAGATGGCAAGAAGAAAAAAATGTTGAACGTTGACACCTTTGGACAAGCTTCTGCCAGACCAAGATTTCACTACCAAAA
G05-X83417-U7551BL150-2-1-2-seq2r.ab1(1>812)  ←    CGTCTTTTTTCGACACCATCTCCACTTCTACTGAAACTAATACCAATATGAGATGGCAAGAAGAAAAAAATGTTGAACGTTGACACCTTTGGACAAGCTTCTGCCAGACCAAGATTTCACTACCAAAA
```

1489 — after deleted fragment

```
                                                   1400      1410      1420      1430      1440      1450      1460      1470      1480      1490      1500      1510      1520
                                                   TGATAGTGGCGCCTATCAGAACCAAAACGACTCGTACAGCAGACCAGCCAACCAGTTTTCGCAACCTCCTTCCAACGTTGAATTTCTCGAGGGTGGTGGTGGTGGTTCTGCTTCTAACTTTACTCAG
U7551BL150-2.seq(1>3168)                      →    TGATAGTGGCGCCTATCAGAACCAAAACGACTCGTACAGCAGACCAGCCAACCAGTTTTCGCAACCTCCTTCCAACGTTGAATTTCTCGAGGGTGGTGGTGGTGGTTCTGCTTCTAACTTTACTCAG
H09-X83417-U7551BL150-2-1-1-SEQ1.ab1(1>823)   →    TGATAGTGGCGCCTATCAGAACCAAAACGACTCGTACAGCAGACCAGCCAACCAGTTTTCGCAACCTCCTTCCAACGTTGAATTTCTCGAGGGTGGTGGTGGTGGTTCTGCTTCTAACTTTACTCAG
G05-X83417-U7551BL150-2-1-2-seq2r.ab1(1>812)  ←    TGATAGTGGCGCCTATCAGAACCAAAACGACTCGTACAGCAGACCAGCCAACCAGTTTTCGCAACCTCCTTCCAACGTTGAATTTCTCGAGGGTGGTGGTGGTGGTTCTGCTTCTAACTTTACTCAG
```

linker | MS2

```
                                                   1530      1540      1550      1560      1570      1580      1590      1600      1610      1620      1630      1640      1650
                                                   TTCGTTCTCGTCGACAATGGCGGAACTGGCGACGTGACTGTCGCCCCAAGCAACTTCGCTAACGGGGTCGCTGAATGGATCAGCTCTAACTCGCGATCACAGGCTTACAAAGTAACCTGTAGCGTTC
U7551BL150-2.seq(1>3168)                      →    TTCGTTCTCGTCGACAATGGCGGAACTGGCGACGTGACTGTCGCCCCAAGCAACTTCGCTAACGGGGTCGCTGAATGGATCAGCTCTAACTCGCGATCACAGGCTTACAAAGTAACCTGTAGCGTTC
H09-X83417-U7551BL150-2-1-1-SEQ1.ab1(1>823)   →    TTCGTTCTCGTCGACAATGGCGGAACTGGCGACGTGACTGTCGCCCCAAGCAACTTCGCTAACGGGGTCGCTGAATGGATCAGCTCTAACTCGCGATCACAGGCTTACAAAGTAACCTGTAGCGTTC
G05-X83417-U7551BL150-2-1-2-seq2r.ab1(1>812)  ←    TTCGTTCTCGTCGACAATGGCGGAACTGGCGACGTGACTGTCGCCCCAAGCAACTTCGCTAACGGGGTCGCTGAATGGATCAGCTCTAACTCGCGATCACAGGCTTACAAAGTAACCTGTAGCGTTC
```

MS2

```
                                                   1660      1670      1680      1690      1700      1710      1720      1730      1740      1750      1760      1770
                                                   GTCAGAGCTCTGCGCAGAATCGCAAATACACCATCAAAGTCGAGGTGCCTAAAGTGGCAACCCAGACTGTTGGTGGTGAAGAGCTTCCTGTAGCCGGATGGAGATCTTACTTAAATATGGAACTAAC
U7551BL150-2.seq(1>3168)                      →    GTCAGAGCTCTGCGCAGAATCGCAAATACACCATCAAAGTCGAGGTGCCTAAAGTGGCAACCCAGACTGTTGGTGGTGAAGAGCTTCCTGTAGCCGGATGGAGATCTTACTTAAATATGGAACTAAC
H09-X83417-U7551BL150-2-1-1-SEQ1.ab1(1>823)   →    GTCAGAGCTCTGCGCAGAATCGCAAATACACCA
G05-X83417-U7551BL150-2-1-2-seq2r.ab1(1>812)  ←    GTCAGAGCTCTGCGCAGAATCGCAAATACACCATCAAAGTCGAGGTGCCTAAAGTGGCAACCCAGACTGTTGGTGGTGAAGAGCTTCCTGTAGCCGGATGGAGATCTTACTTAAATATGGAACTAAC
```

MS2

```
                                                   1780      1790      1800      1810      1820      1830      1840      1850      1860      1870      1880      1890      1900
                                                   CATTCCAATTTTCGCCACGAATTCCGACTGCGAGCTTATTGTTAAGGCAATGCAAGGTCTCCTAAAAGATGGAAACCCGATTCCCTCGGCCATCGCAGCAAACTCCGGCATCTACGACTACAAAGAC
U7551BL150-2.seq(1>3168)                      →    CATTCCAATTTTCGCCACGAATTCCGACTGCGAGCTTATTGTTAAGGCAATGCAAGGTCTCCTAAAAGATGGAAACCCGATTCCCTCGGCCATCGCAGCAAACTCCGGCATCTACGACTACAAAGAC
G05-X83417-U7551BL150-2-1-2-seq2r.ab1(1>812)  ←    CATTCCAATTTTCGCCACGAATTCCGACTGCGAGCTTATTGTTAAGGCAATGCAAGGTCTCCTAAAAGATGGAAACCCGATTCCCTCGGCCATCGCAGCAAACTCCGGCATCTACGACTACAAAGAC
```

MS2

```
                                                   1910      1920      1930      1940      1950      1960      1970      1980      1990      2000      2010      2020      2030
                                                   CATGACGGTGATTATAAAGATCATGACATCGACTACAAGGATGACGATGACAAGTAAAATGATGTTTCTATGTAAATTAAGTATATCATTCAGTATGTACATACAAAAATCAATTAATAGTATTAT
U7551BL150-2.seq(1>3168)                      →    CATGACGGTGATTATAAAGATCATGACATCGACTACAAGGATGACGATGACAAGTAAAATGATGTTTCTATGTAAATTAAGTATATCATTCAGTATGTACATACAAAAATCAATTAATAGTATTAT
G05-X83417-U7551BL150-2-1-2-seq2r.ab1(1>812)  ←    CATGACGGTGATTATAAAGATCATGACATCGACTACAAGGATGACGATGACAAGTAAAATGATGTTTCTATGTAAATTAAGTATATCATTCAGTATGTACATACAAAAATCAATTAATAGTATTAT
H06-X83417-U7551BL150-2-1-1-SEQ3.ab1(1>828)   →                                                                                                 GTACATACAAAAATCAATTAATAGTATTAT
```

STOP

```
2040 2050 2060 2070 2080 2090 2100 2110 2120 2130 2140 2150
AGTCCGCGCCTCTGGATAGTATTATTTTAGTGCGTATCGCAAAATATGAATAGCACAGAGGTTCATTATTATGTTCTGAGTATGAGGACGAGGTAATACATACCTTAAAATCTTACATCATGTGGAT
U7551BL150-2.seq(1>3168)                        →  AGTCCGCGCCTCTGGATAGTATTATTTTAGTGCGTATCGCAAAATATGAATAGCACAGAGGTTCATTATTATGTTCTGAGTATGAGGACGAGGTAATACATACCTTAAAATCTTACATCATGTGGAT
G05-X83417-U7551BL150-2-1-2-seq2r.ab1(1>812)    ←  A
H06-X83417-U7551BL150-2-1-1-SEQ3.ab1(1>828)     →  AGTCCGCGCCTCTGGATAGTATTATTTTAGTGCGTATCGCAAAATATGAATAGCACAGAGGTTCATTATTATGTTCTGAGTATGAGGACGAGGTAATACATACCTTAAAATCTTACATCATGTGGAT
```

```
2160 2170 2180 2190 2200 2210 2220 2230 2240 2250 2260 2270 2280
AGCATTTATGGCTTCTTCCATCCGGCCGTACGCTTCTGTTCCTGTCCCTAGCCATAGCTTTTCTCATATCAAAAGCATCTTTATTGTCATCTACTTTTTCAGGGTATCTGGATACCCATCTTCAGC
U7551BL150-2.seq(1>3168)                        →  AGCATTTATGGCTTCTTCCATCCGGCCGTACGCTTCTGTTCCTGTCCCTAGCCATAGCTTTTCTCATATCAAAAGCATCTTTATTGTCATCTACTTTTTCAGGGTATCTGGATACCCATCTTCAGC
H06-X83417-U7551BL150-2-1-1-SEQ3.ab1(1>828)     →  AGCATTTATGGCTTCTTCCATCCGGCCGTACGCTTCTGTTCCTGTCCCTAGCCATAGCTTTTCTCATATCAAAAGCATCTTTATTGTCATCTACTTTTTCAGGGTATCTGGATACCCATCTTCAGC
```

```
2290 2300 2310 2320 2330 2340 2350 2360 2370 2380 2390 2400 2410
TGAGTTATAATATCCCACCACTCTCCTGTAGACACTGTATCCCAATTTTTCGTAAAGTTTGATGGCCAATTGGTTATTGCACTTGACGAAAAGATCGATAAAGTTGACTTCATGGGGCATGACATCG
U7551BL150-2.seq(1>3168)                        →  TGAGTTATAATATCCCACCACTCTCCTGTAGACACTGTATCCCAATTTTTCGTAAAGTTTGATGGCCAATTGGTTATTGCACTTGACGAAAAGATCGATAAAGTTGACTTCATGGGGCATGACATCG
H06-X83417-U7551BL150-2-1-1-SEQ3.ab1(1>828)     →  TGAGTTATAATATCCCACCACTCTCCTGTAGACACTGTATCCCAATTTTTCGTAAAGTTTGATGGCCAATTGGTTATTGCACTTGACGAAAAGATCGATAAAGTTGACTTCATGGGGCATGACATCG
H07-X83417-U7551BL150-2-M13F-77.ab1(1>837)      ←  GCATGACATCG
```

```
2420 2430 2440 2450 2460 2470 2480 2490 2500 2510 2520 2530 2540
GTCATGGTCTCCAAAGTATTACATAACTTCGAAGCTAGCGATATTCGGCGGAATCTTGGAGCTACTGTCACTGCAGTTATATGAGTGTGCCACTCCGTGGTCTTGCCTTCTGTTTTTGCCATCATGT
U7551BL150-2.seq(1>3168)                        →  GTCATGGTCTCCAAAGTATTACATAACTTCGAAGCTAGCGATATTCGGCGGAATCTTGGAGCTACTGTCACTGCAGTTATATGAGTGTGCCACTCCGTGGTCTTGCCTTCTGTTTTTGCCATCATGT
H06-X83417-U7551BL150-2-1-1-SEQ3.ab1(1>828)     →  GTCATGGTCTCCAAAGTATTACATAACTTCGAAGCTAGCGATATTCGGCGGAATCTTGGAGCTACTGTCACTGCAGTTATATGAGTGTGCCACTCCGTGGTCTTGCCTTCTGTTTTTGCCATCATGT
H07-X83417-U7551BL150-2-M13F-77.ab1(1>837)      ←  GTCATGGTCTCCAAAGTATTACATAACTTCGAAGCTAGCGATATTCGGCGGAATCTTGGAGCTACTGTCACTGCAGTTATATGAGTGTGCCACTCCGTGGTCTTGCCTTCTGTTTTTGCCATCATGT
```

```
2550 2560 2570 2580 2590 2600 2610 2620 2630 2640 2650 2660
AACCAGAGATATTATGCTTAAATGTAGGATCAACAGTCATTTCTGAACTTTTGAAAAAAAGGTCTGGCCATATTATCATGTATTCAAAATAAAACTCTAAAGGGAAGTTTTCTGTTAAGATATCAAG
U7551BL150-2.seq(1>3168)                        →  AACCAGAGATATTATGCTTAAATGTAGGATCAACAGTCATTTCTGAACTTTTGAAAAAAAGGTCTGGCCATATTATCATGTATTCAAAATAAAACTCTAAAGGGAAGTTTTCTGTTAAGATATCAAG
H06-X83417-U7551BL150-2-1-1-SEQ3.ab1(1>828)     →  AACCAGAGATATTATGCTTAAATGTAGGATCAACAGTCATTTCTGAACTTTTGAAAAAAAGGTCTGGCCATATTATCATGTATTCAAAATAAAACTCTAAAGGGAAGTTTTCTGTTAAGATATCAAG
H07-X83417-U7551BL150-2-M13F-77.ab1(1>837)      ←  AACCAGAGATATTATGCTTAAATGTAGGATCAACAGTCATTTCTGAACTTTTGAAAAAAAGGTCTGGCCATATTATCATGTATTCAAAATAAAACTCTAAAGGGAAGTTTTCTGTTAAGATATCAAG
```

```
2670 2680 2690 2700 2710 2720 2730 2740 2750 2760 2770 2780 2790
GTTGACATTGTTAGTTTTGAATAAATCGACAGGTTCAAAAGGCTGAATTGTTGTCATTTCTCGAATCCTCCTGTCTCTTTCCTTGAATATTCTCAATGTTCCACTATCTTTCACATAAGCAATATTG
U7551BL150-2.seq(1>3168)                        →  GTTGACATTGTTAGTTTTGAATAAATCGACAGGTTCAAAAGGCTGAATTGTTGTCATTTCTCGAATCCTCCTGTCTCTTTCCTTGAATATTCTCAATGTTCCACTATCTTTCACATAAGCAATATTG
H06-X83417-U7551BL150-2-1-1-SEQ3.ab1(1>828)     →  GTTGACATTGTTAGTTTTGAATAAATCGACAGGTTCAAAAGGCTGAATTGTTGTCATTTCTCGAATCCTCCTGTCTCTTTCCTTGAATATTCTCAATGTTCCACTATCTTTCACATAAGCAATATTG
H07-X83417-U7551BL150-2-M13F-77.ab1(1>837)      ←  GTTGACATTGTTAGTTTTGAATAAATCGACAGGTTCAAAAGGCTGAATTGTTGTCATTTCTCGAATCCTCCTGTCTCTTTCCTTGAATATTCTCAATGTTCCACTATCTTTCACATAAGCAATATTG
```

```
2800 2810 2820 2830 2840 2850 2860 2870 2880 2890 2900 2910 2920
TGAACTATATATACTTCATTAGATATTGCTTCAACATTAATATTAATAAAAAGGACATGCATAAAGTAACATCCGAACATCTCCAAACACCCATACATATTTATATTATCGACGAGGGAATTTTTTT
U7551BL150-2.seq(1>3168)                        →  TGAACTATATATACTTCATTAGATATTGCTTCAACATTAATATTAATAAAAAGGACATGCATAAAGTAACATCCGAACATCTCCAAACACCCATACATATTTATATTATCGACGAGGGAATTTTTTT
H06-X83417-U7551BL150-2-1-1-SEQ3.ab1(1>828)     →  TGAACTATATATACTTCATTAGATATTGCTTCAAC
H07-X83417-U7551BL150-2-M13F-77.ab1(1>837)      ←  TGAACTATATATACTTCATTAGATATTGCTTCAACATTAATATTAATAAAAAGGACATGCATAAAGTAACATCCGAACATCTCCAAACACCCATACATATTTATATTATCGACGAGGGAATTTTTTT
```

```
2930 2940 2950 2960 2970 2980 2990 3000 3010 3020 3030 3040
CTTCCCAGGCCCACCAACGTGCGCCCACCGCTATGTTTTTTGCGCCTAGGTAGGTGGGCTAGCTTCTATGTATCAAAGCTCCTTCTGCGCAGCCGAGGTTTTGCTAAGCACTACCGCATTGGAATGA
U7551BL150-2.seq(1>3168)                        →  CTTCCCAGGCCCACCAACGTGCGCCCACCGCTATGTTTTTTGCGCCTAGGTAGGTGGGCTAGCTTCTATGTATCAAAGCTCCTTCTGCGCAGCCGAGGTTTTGCTAAGCACTACCGCATTGGAATGA
H07-X83417-U7551BL150-2-M13F-77.ab1(1>837)      ←  CTTCCCAGGCCCACCAACGTGCGCCCACCGCTATGTTTTTTGCGCCTAGGTAGGTGGGCTAGCTTCTATGTATCAAAGCTCCTTCTGCGCAGCCGAGGTTTTGCTAAGCACTACCGCATTGGAATGA
```

		3050 3060 3070 3080 3090 3100 3110 3120 3130 3140 3150 3160 3170
		CTGTGTTTGTAGGAGCTCCTTGTCTCCACGCTTTCGCAATTTTCAAGATCGAGATGCTATTAGTGATGGTAAGGTTATGAAGCTTACATGTTTAATATTGTAGATTCCAGAAGCAGGTAAGAACAAC
U7551BL150-2.seq(1>3168)	→	CTGTGTTTGTAGGAGCTCCTTGTCTCCACGCTTTCGCAATTTTCAAGATCGAGATGCTATTAGTGATGGTAAGGTTATGAAGCTTACATGTTTAATATTGTAGATTCCAGAAGCAGGTAAGAACAAC
H07-X83417-U7551BL150-2-M13F-77.ab1(1>837)	←	CTGTGTTTGTAGGAGCTCCTTGTCTCCACGCTTTCGCAATTTTCAAGATCGAGATGCTATTAGTGATGGTAAGGTTATGAAGCTTACATGTTTAATATTGTAGATTCCAGAAGCAGGTAAGAACAAC

		3180 3190 3200 3210 3220 3230
		AAACTACAAGAAAAGGGTACCCGGGTACCGAGCTCGAATTCACTGGCCGTCGTTtTACAACGTC
U7551BL150-2.seq(1>3168)	→	AAACTACAAGAAAAGGGTACCCGGG
H07-X83417-U7551BL150-2-M13F-77.ab1(1>837)	←	AAACTACAAGAAAAGGGTACCCGGGTACCGAGCTCGAATTCACTGGCCGTCGTTtTACAACGTC

SmaI

Certificate of Analysis

Project ID: U7551BL150-2

Construct Information:

Gene Name: DeltaRGG-Scd6-MS2

Clone ID: X83417

Gene Length: 3167 bp

Cloning Vector: YCpLac111

Cloning Strategy: SphI / SmaI

QC Items	Specifications	Results	
**Sequencing Alignment**	Sequencing results are consistent with the targeted insert sequence.	Pass	Consistent
**Vector Sequence**	The flanking sequences of the cloning site are correct.	Pass	Correct<br>Shown in the SQD file
**Restriction Digests**	The size of inserted fragment is correct and free of unexpected bands suggesting contamination.	Pass	Correct<br>Shown in attachment 1
**DNA Quality**	Miniprep: 4 μg<br>OD260/280=1.8~2.0<br>Free of contamination	Pass	≥ 4 μg<br>OD260/280=1.86<br>Pure
**Quality grade**	Research Grade	Pass	Research Grade
**Appearance**	Clear and free of foreign particles.	Pass	Clear<br>Free of foreign particles
**Additional Test**		N/A	

NOTE

Shipping at	Plasmid Storing at	Bacstab Storing at	Glycerol Stock Storing at
Room Temperature	-20℃	4℃	-20℃/-80℃

Morgan

Certified by: **Date: 02/12/2017**

Thank you for your patronage to our Gene services! To maintain this working relationship, we shall be grateful if you can add our webpage URL into your lab website. As a token of appreciation, you will be rewarded by 1,000 EZcoupon™ points. For more information, please contact us by e-mail at web@genscript.com.

For research use only

Attachment 1

Plasmid Construct Map

The gene was cloned in YCpLac111 by SphI / SmaI.

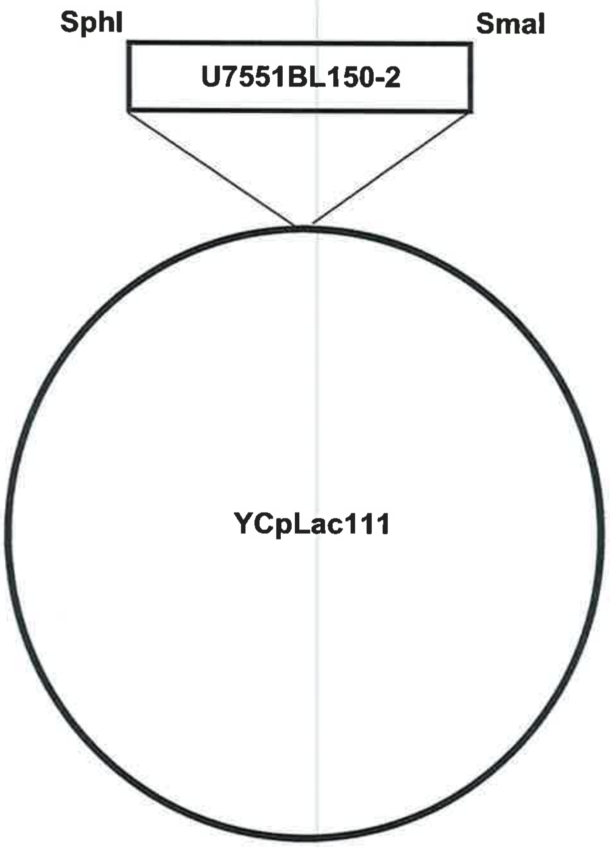

Enzyme Digestion

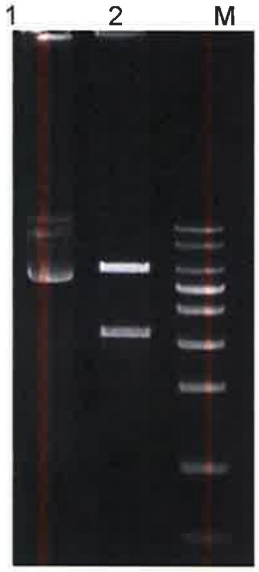

Lane M: KB Ladder
Lane 1:U7551BL150-2 plasmid
Lane 2:U7551BL150-2 plasmid digested
by SphI and SmaI

Digestion Conditions:
About 300ng plasmid digested
Digestion in water-bath,37℃ for 40 minutes
1% Agarose Gel

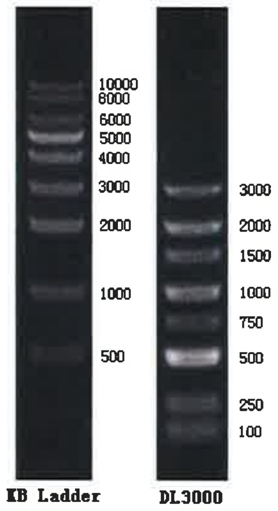

For research use only

860 Centennial Ave., Piscataway, NJ 08854, USA
Toll-Free: 1-877-436-7274 Tel: 1-732-885-9188 Fax: 1-732-210-0262 Email: order@genscript.com Web: www.genscript.com

Scd6 multiple sequence alignment by T-Coffee

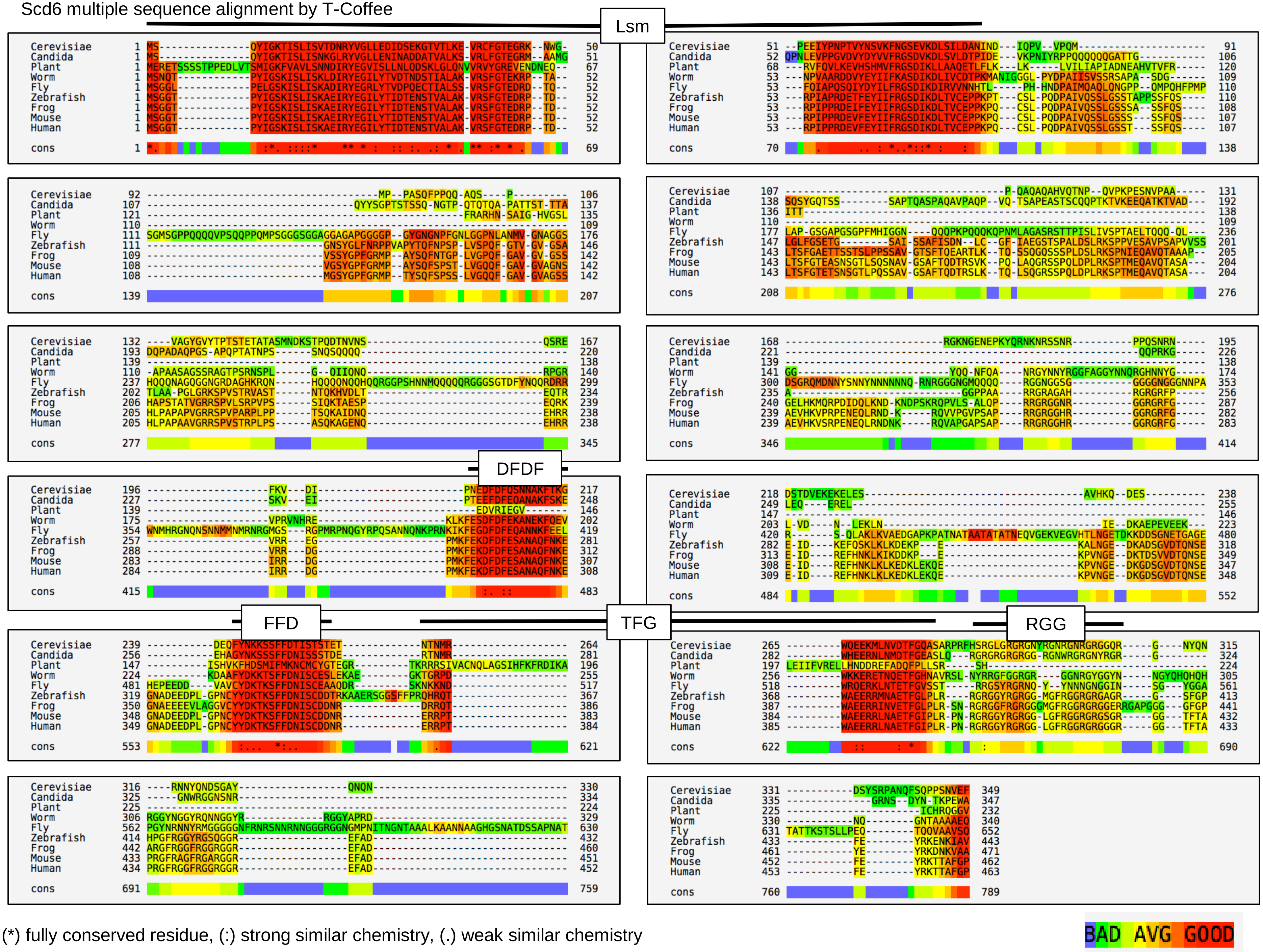

(*) fully conserved residue, (:) strong similar chemistry, (.) weak similar chemistry
